# Supplementary material for: Deconstructing the Monolith: An Educational Module for Understanding Disparities Within Asian American, Native Hawaiian, and Pacific Islander Populations
Source: MedEdPORTAL. 2025 Jan 7;21:11480. doi: 10.15766/mep_2374-8265.11480 (PMC11697750; doi:10.15766/mep_2374-8265.11480)
Supplement: Supplementary file 1 — Monolith To Mosaic Presentation.pptxFacilitator Guide.docxPresurvey.docxPostsurvey.docx [file mep_2374-8265.11480-s001.zip › D. Postsurvey.docx]

**POST - Workshop Survey**

This is a 14-question Qualtrics evaluation distributed to by participants after the workshop. This evaluation contains the same set of questions as the pre-workshop evaluation form, with additional questions eliciting participant feedback about the quality of the workshop. We recommend giving participants 5 minutes to complete this evaluation following the presentation.

Please enter your assigned identification number ____________________

Please evaluate your preparedness in completing the following three learning objectives:

|  | Not at all prepared | Slightly prepared | Moderately prepared | Very prepared |
| --- | --- | --- | --- | --- |
| DESCRIBING the historical context of the “Asian Monolith” stereotype in the United States. |  |  |  |  |
| OUTLINING 3 ways in which the “Asian Monolith” stereotype negatively impacts health outcomes for diverse Asian communities. |  |  |  |  |
| IDENTIFYING strategies to improve healthcare equity for Asian patients and communities. |  |  |  |  |

Please state the extent to which you agree with the following statements

|  | Strongly agree | Agree | Neither agree nor disagree | Disagree | Strongly disagree |
| --- | --- | --- | --- | --- | --- |
| I am confident in my ability to  take care of AANHPI patients |  |  |  |  |  |
| I am knowledgeable about the diversity of AANHPI patients |  |  |  |  |  |
| My medical school does a good job at teaching the different healthcare needs for AANHPI subpopulations |  |  |  |  |  |

**POST - Workshop Survey**

Multiple Choice

1. Which movement in healthcare research describes the push-back against averaging the data of all Asians into 1 category?
   1. Dissolution
   2. Disaggregation
   3. strict analysis
   4. ethnic coding
2. Which of the following Asian American, Native Hawaiian and Pacific Islanders (AANHPI) subgroups represented the smallest percentage of U.S. medical school applicants in 2018:
   1. Bangladeshi
   2. Japanese
   3. Cambodian
   4. Laotian
3. Which of the following AANHPI subgroups has the lowest rates of mammogram screening
   1. Indian
   2. Korean
   3. Vietnamese
   4. Laotian
4. Which of the following AANHPI subgroups has the highest smoking rates?
   1. Chinese
   2. Filipino
   3. Pakistani
   4. Native Hawaiian
5. What are "culture-bound syndromes" in the context of healthcare?
   1. Syndromes that are uncommon across all cultures
   2. Syndromes that are only acknowledged in Western medicine
   3. Syndromes that are relatively common within certain cultures but not officially acknowledged in Western medicine
   4. Syndromes that can only be treated with traditional medicines

**POST - Workshop Survey**

What did you like about this module

*Free Response*

What suggestions do you have to improve this module?

*Free Response*
