# Supplementary material for: Deconstructing the Monolith: An Educational Module for Understanding Disparities Within Asian American, Native Hawaiian, and Pacific Islander Populations
Source: MedEdPORTAL. 2025 Jan 7;21:11480. doi: 10.15766/mep_2374-8265.11480 (PMC11697750; doi:10.15766/mep_2374-8265.11480)
Supplement: Supplementary file 1 — Monolith To Mosaic Presentation.pptxFacilitator Guide.docxPresurvey.docxPostsurvey.docx [file mep_2374-8265.11480-s001.zip › A. Monolith To Mosaic Presentation.pptx]

## Slide 1
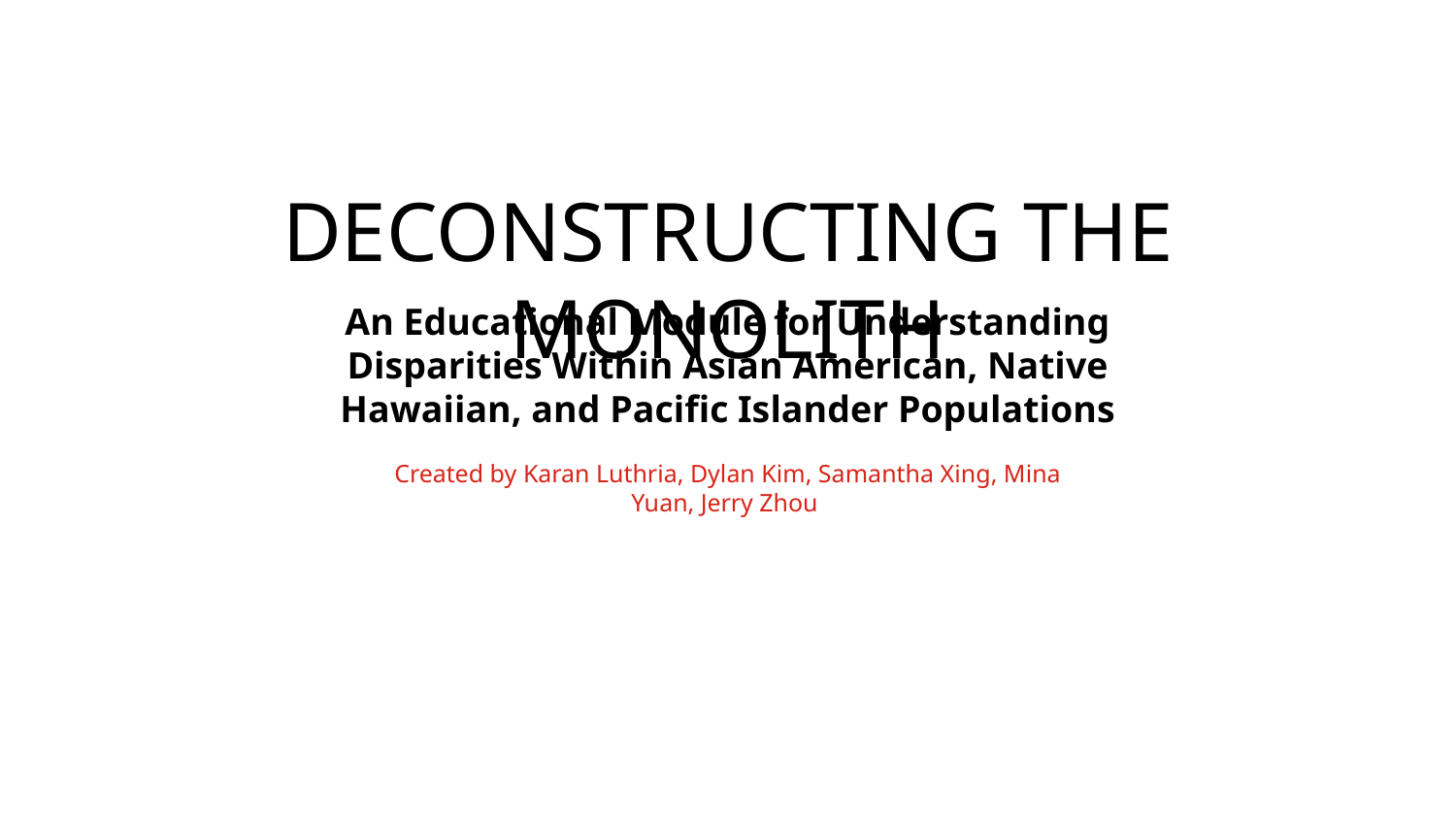

# DECONSTRUCTING THE MONOLITH
An Educational Module for Understanding Disparities Within Asian American, Native Hawaiian, and Pacific Islander Populations
Created by Karan Luthria, Dylan Kim, Samantha Xing, Mina Yuan, Jerry Zhou

## Slide 2
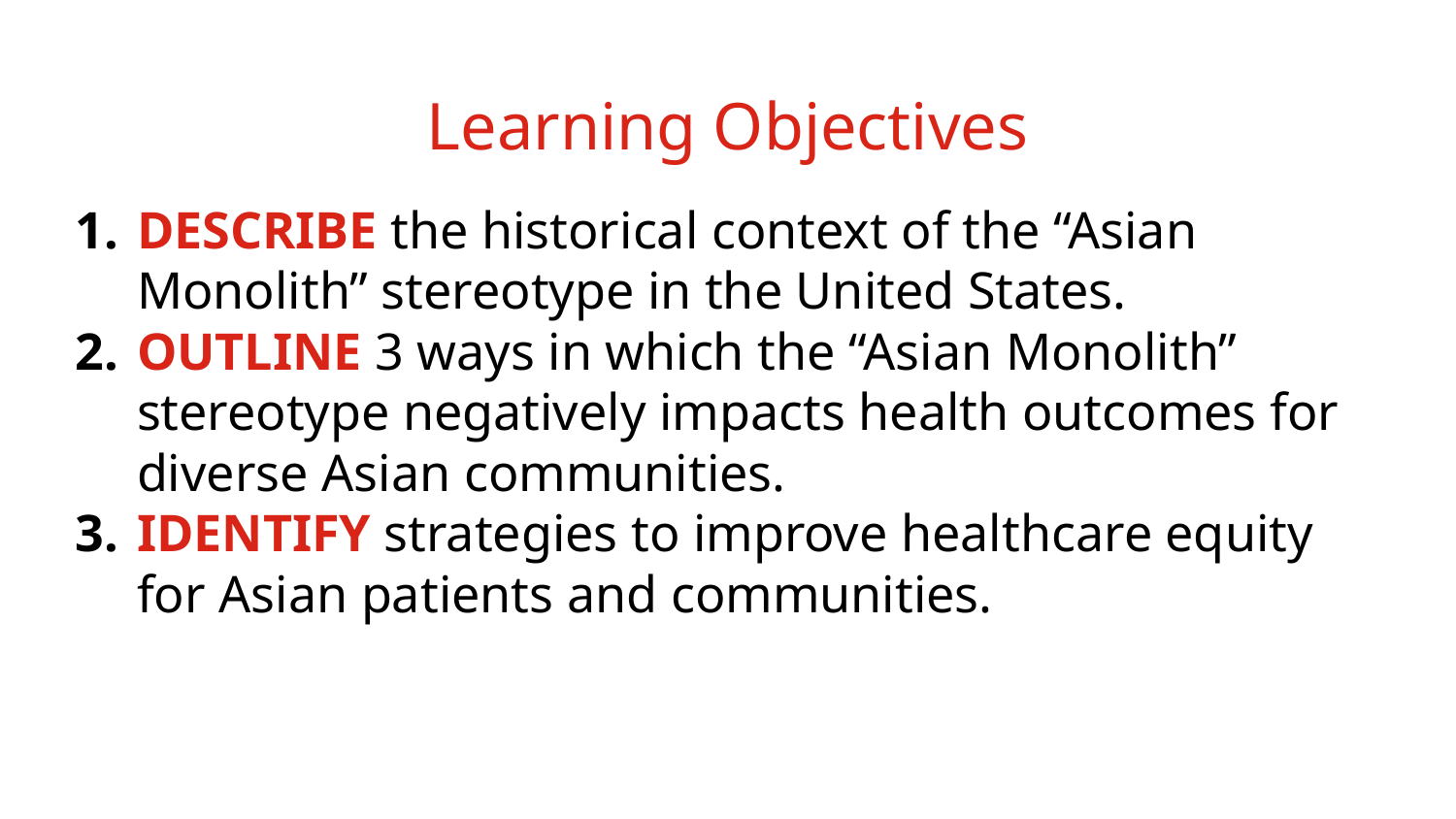

# Learning Objectives
DESCRIBE the historical context of the “Asian Monolith” stereotype in the United States.
OUTLINE 3 ways in which the “Asian Monolith” stereotype negatively impacts health outcomes for diverse Asian communities.
IDENTIFY strategies to improve healthcare equity for Asian patients and communities.

## Slide 3
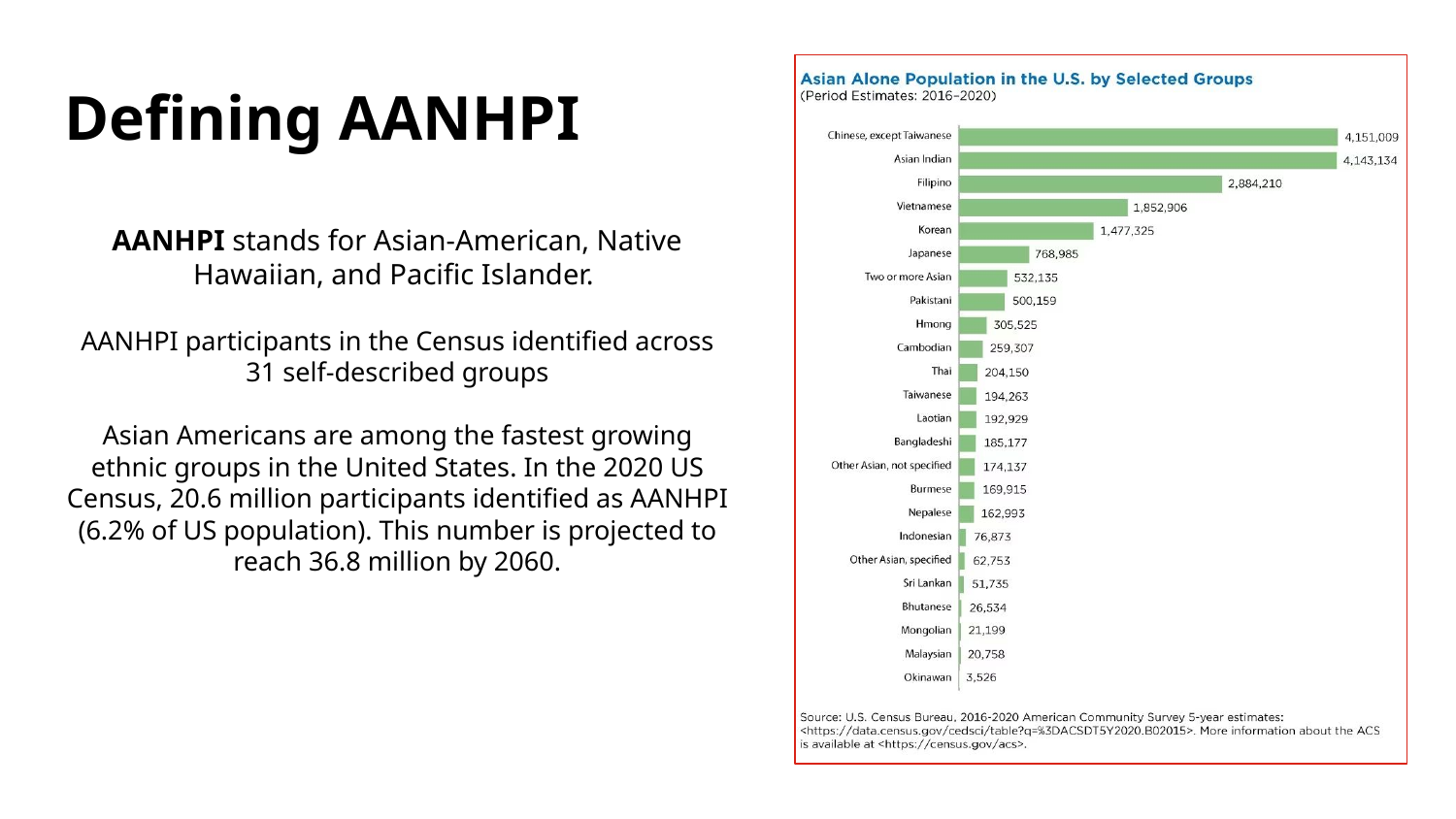

# Defining AANHPI
AANHPI stands for Asian-American, Native Hawaiian, and Pacific Islander.
AANHPI participants in the Census identified across 31 self-described groups
Asian Americans are among the fastest growing ethnic groups in the United States. In the 2020 US Census, 20.6 million participants identified as AANHPI (6.2% of US population). This number is projected to reach 36.8 million by 2060.

## Slide 4
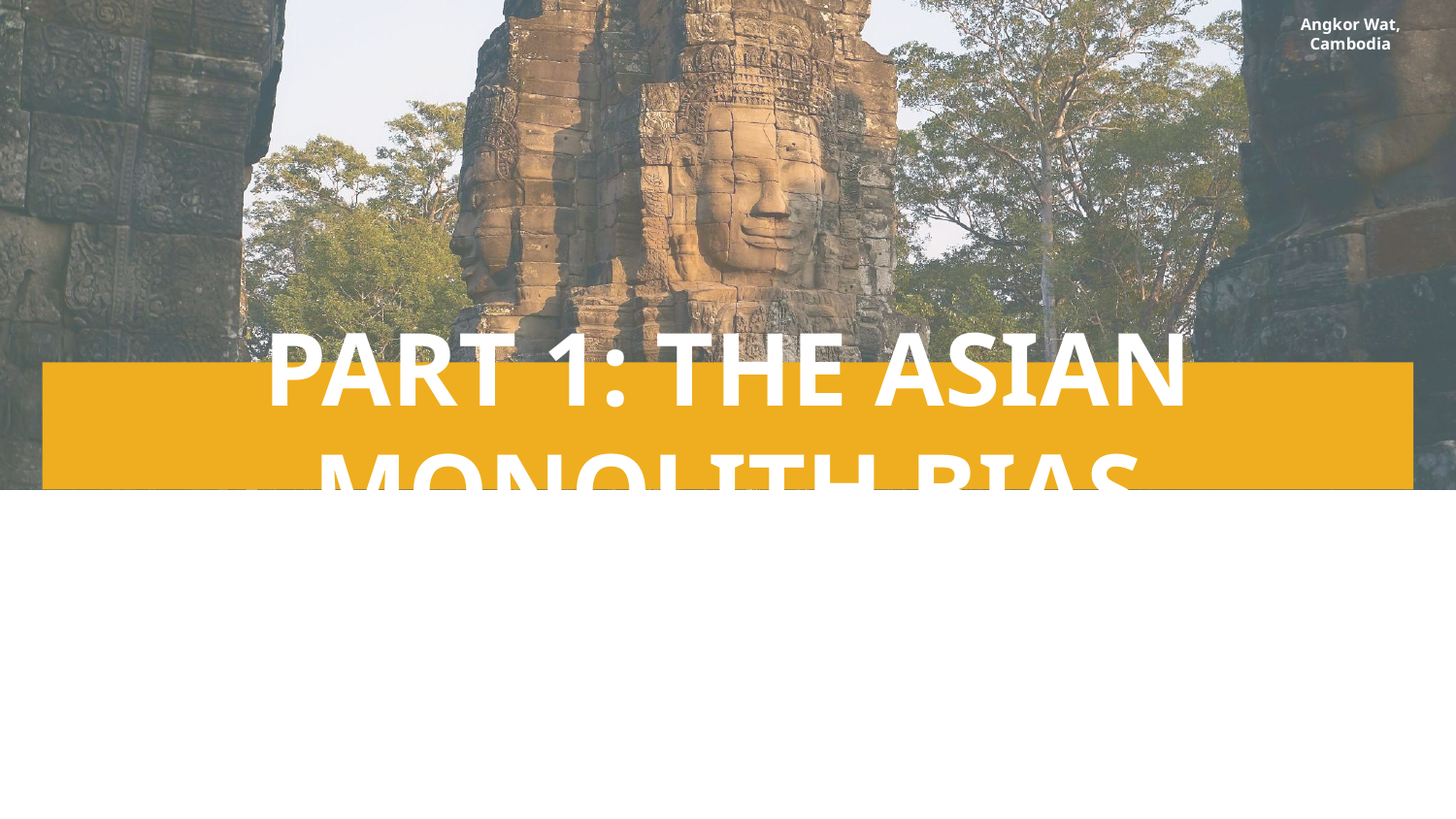

Angkor Wat, Cambodia
PART 1: THE ASIAN MONOLITH BIAS

## Slide 5
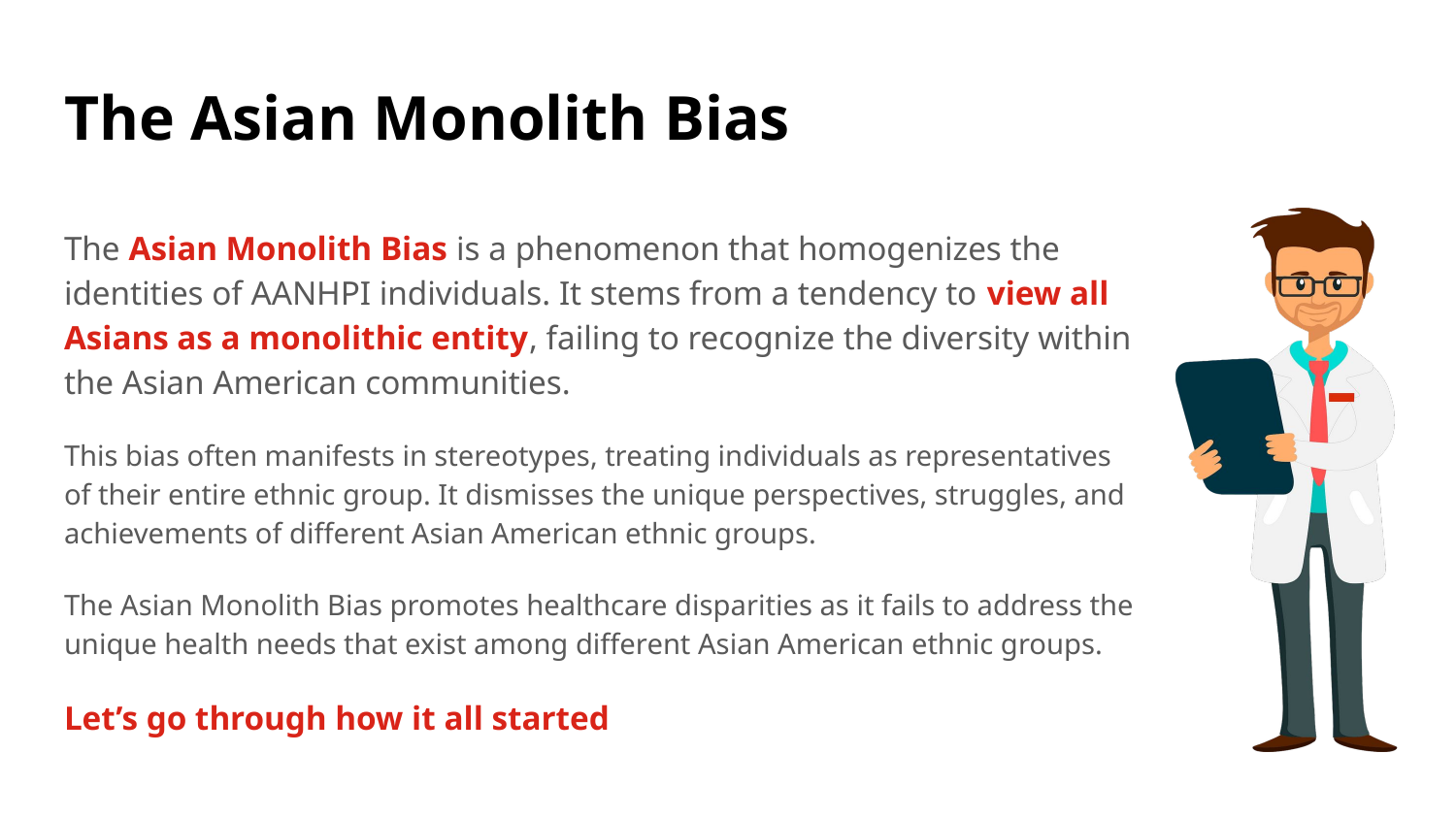

# The Asian Monolith Bias
The Asian Monolith Bias is a phenomenon that homogenizes the identities of AANHPI individuals. It stems from a tendency to view all Asians as a monolithic entity, failing to recognize the diversity within the Asian American communities.
This bias often manifests in stereotypes, treating individuals as representatives of their entire ethnic group. It dismisses the unique perspectives, struggles, and achievements of different Asian American ethnic groups.
The Asian Monolith Bias promotes healthcare disparities as it fails to address the unique health needs that exist among different Asian American ethnic groups.
Let’s go through how it all started

## Slide 6
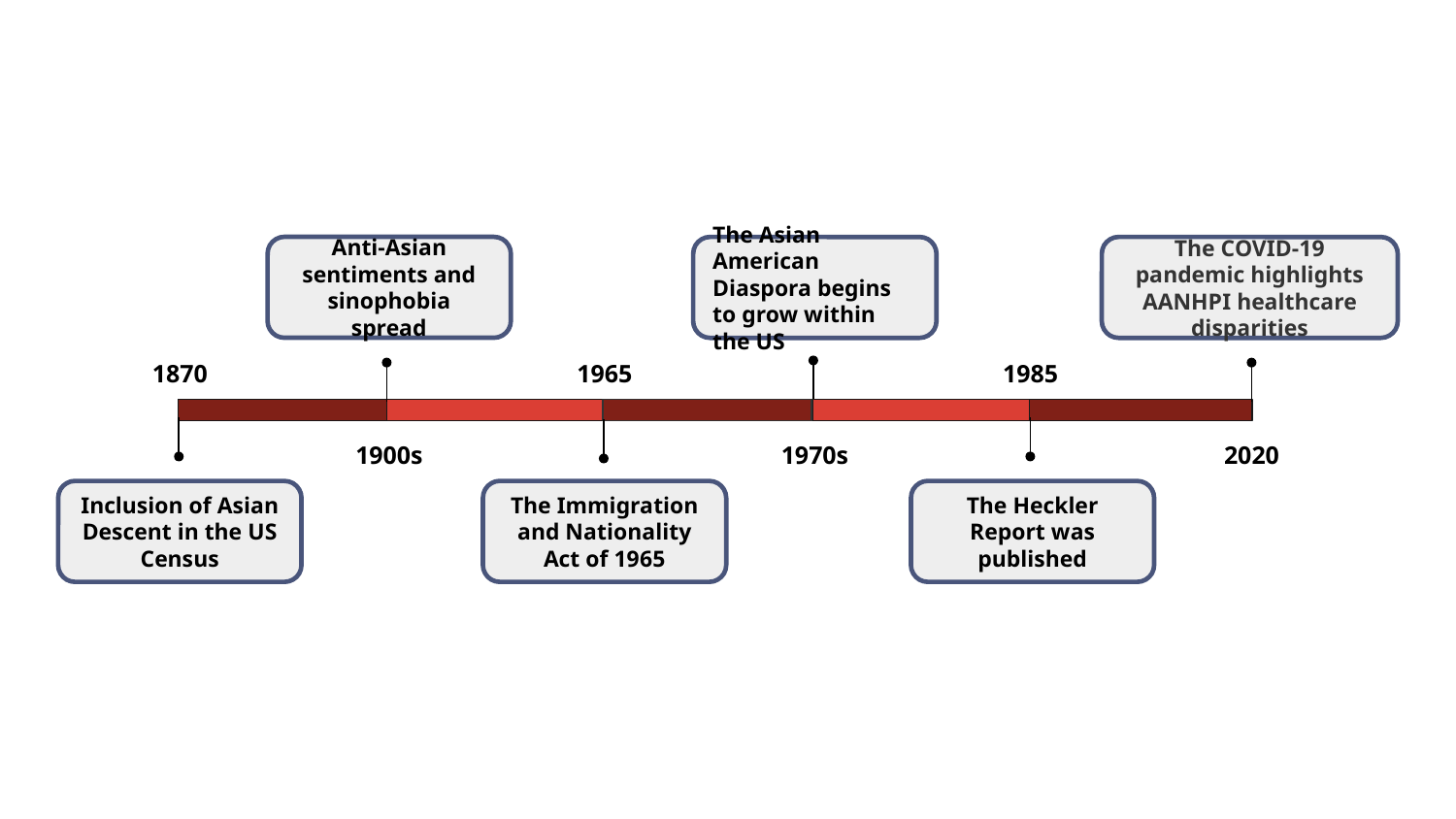

Anti-Asian sentiments and sinophobia spread
The Asian American Diaspora begins to grow within the US
The COVID-19 pandemic highlights AANHPI healthcare disparities
1870
1965
1985
2020
1900s
1970s
Inclusion of Asian Descent in the US Census
The Immigration and Nationality Act of 1965
The Heckler Report was published

## Slide 7
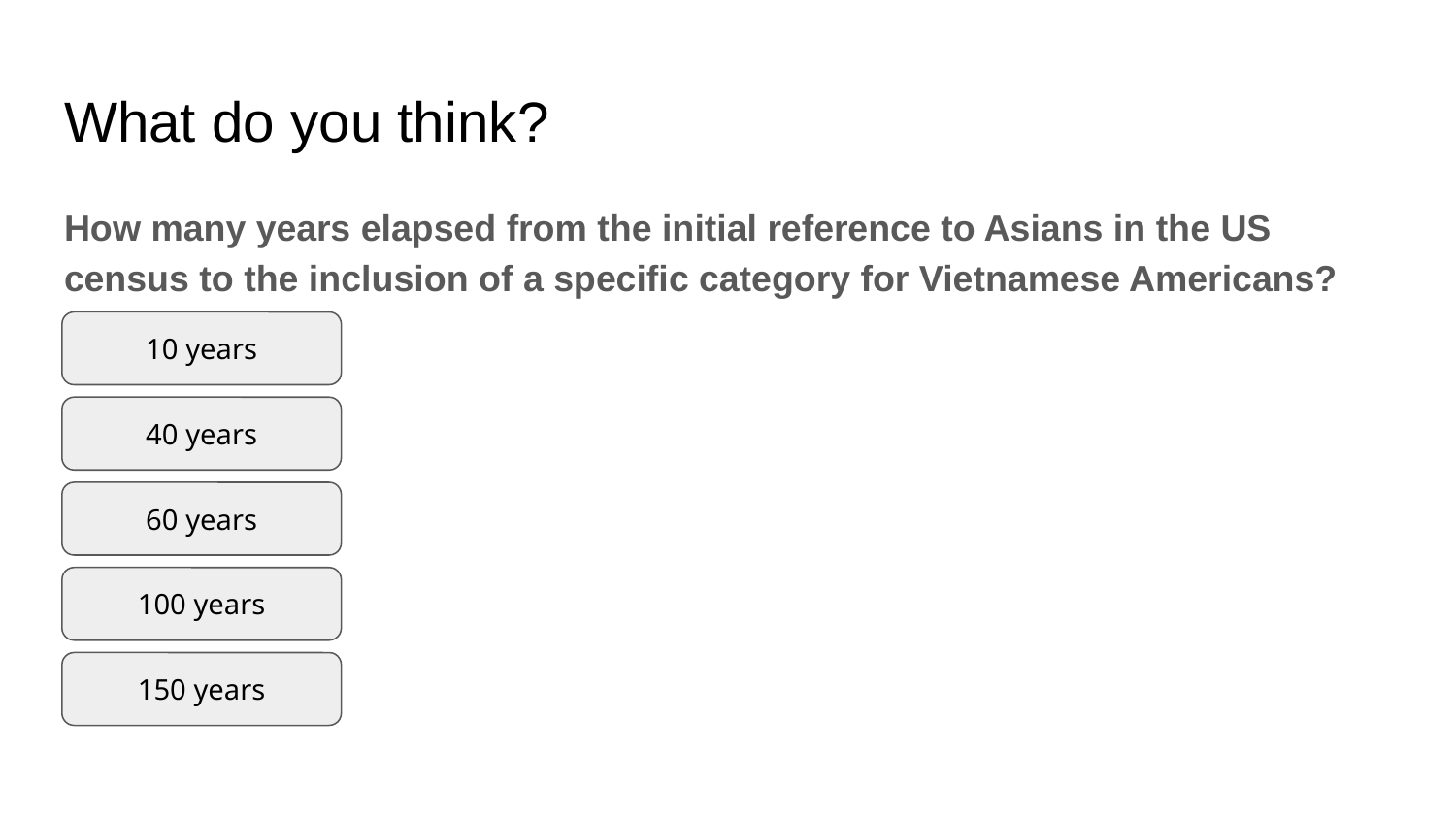

# What do you think?
How many years elapsed from the initial reference to Asians in the US census to the inclusion of a specific category for Vietnamese Americans?
10 years
40 years
60 years
100 years
150 years

## Slide 8
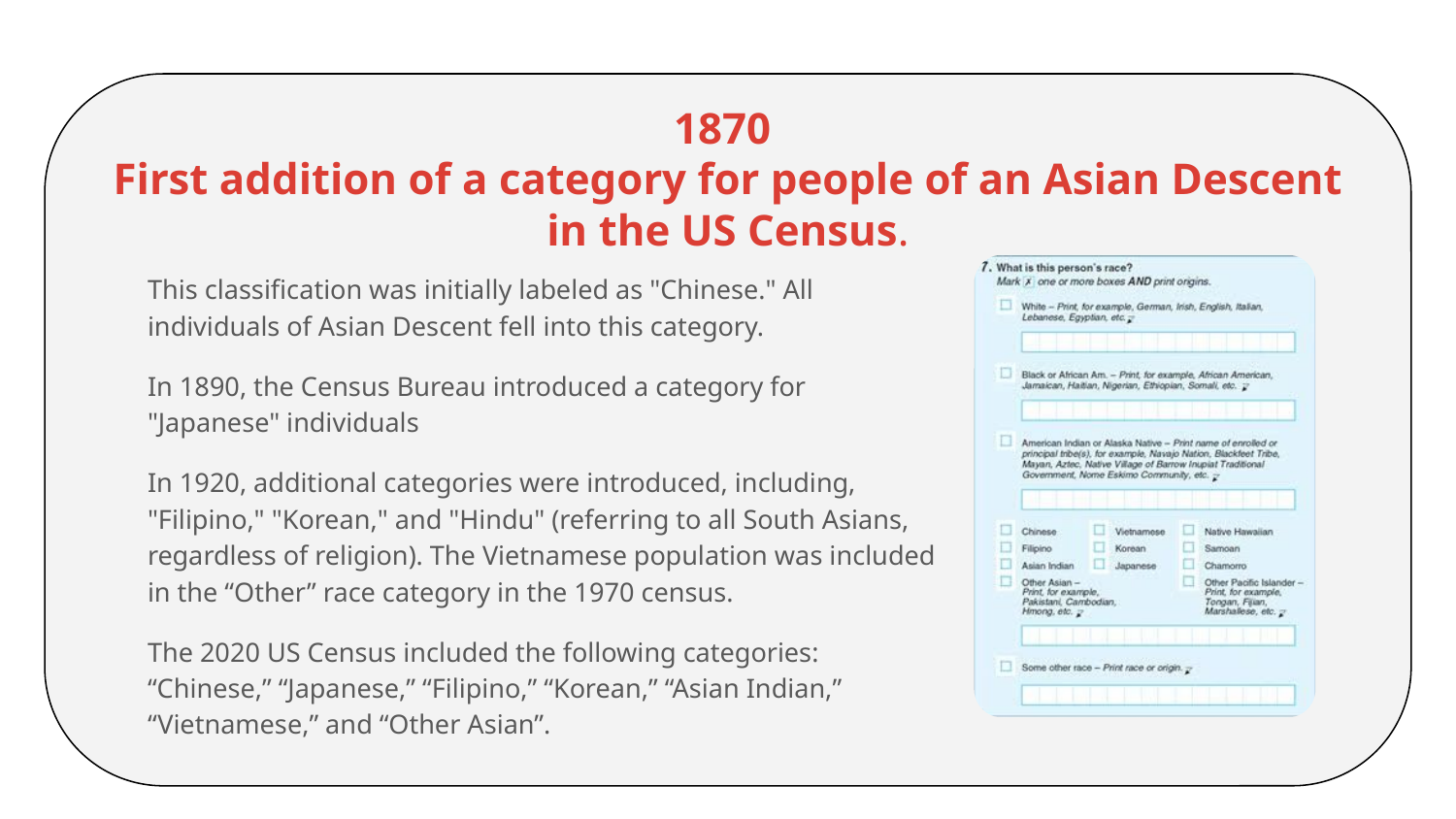

# The Asian Monolith Bias
1870
First addition of a category for people of an Asian Descent in the US Census.
1870: First additional of a category for people of an Asian Descent in the US Census.
This classification was initially labeled as "Chinese." All individuals of Asian Descent fell into this category.
In 1890, the Census Bureau introduced a category for "Japanese" individuals
In 1920, additional categories were introduced, including, "Filipino," "Korean," and "Hindu" (referring to all South Asians, regardless of religion)
The 2020 US Census included the following categories: “Chinese,” “Japanese,” “Filipino,” “Korean,” “Asian Indian,” “Vietnamese,” and “Other Asian”.
Click on each event to learn more!
This classification was initially labeled as "Chinese." All individuals of Asian Descent fell into this category.
In 1890, the Census Bureau introduced a category for "Japanese" individuals
In 1920, additional categories were introduced, including, "Filipino," "Korean," and "Hindu" (referring to all South Asians, regardless of religion). The Vietnamese population was included in the “Other” race category in the 1970 census.
The 2020 US Census included the following categories: “Chinese,” “Japanese,” “Filipino,” “Korean,” “Asian Indian,” “Vietnamese,” and “Other Asian”.
First additional of a category for people of an Asian Descent in the US Census.
1870
The Asian American Diaspora begins to grow within the US
1970s
The AANHPI Population is the US continues to rise
2000s
1965
The Immigration and Nationality Act of 1965
1985
The Heckler Report was published
2020
The COVID-19 Pandemic highlights the growing AANHPI healthcare disparities

## Slide 9
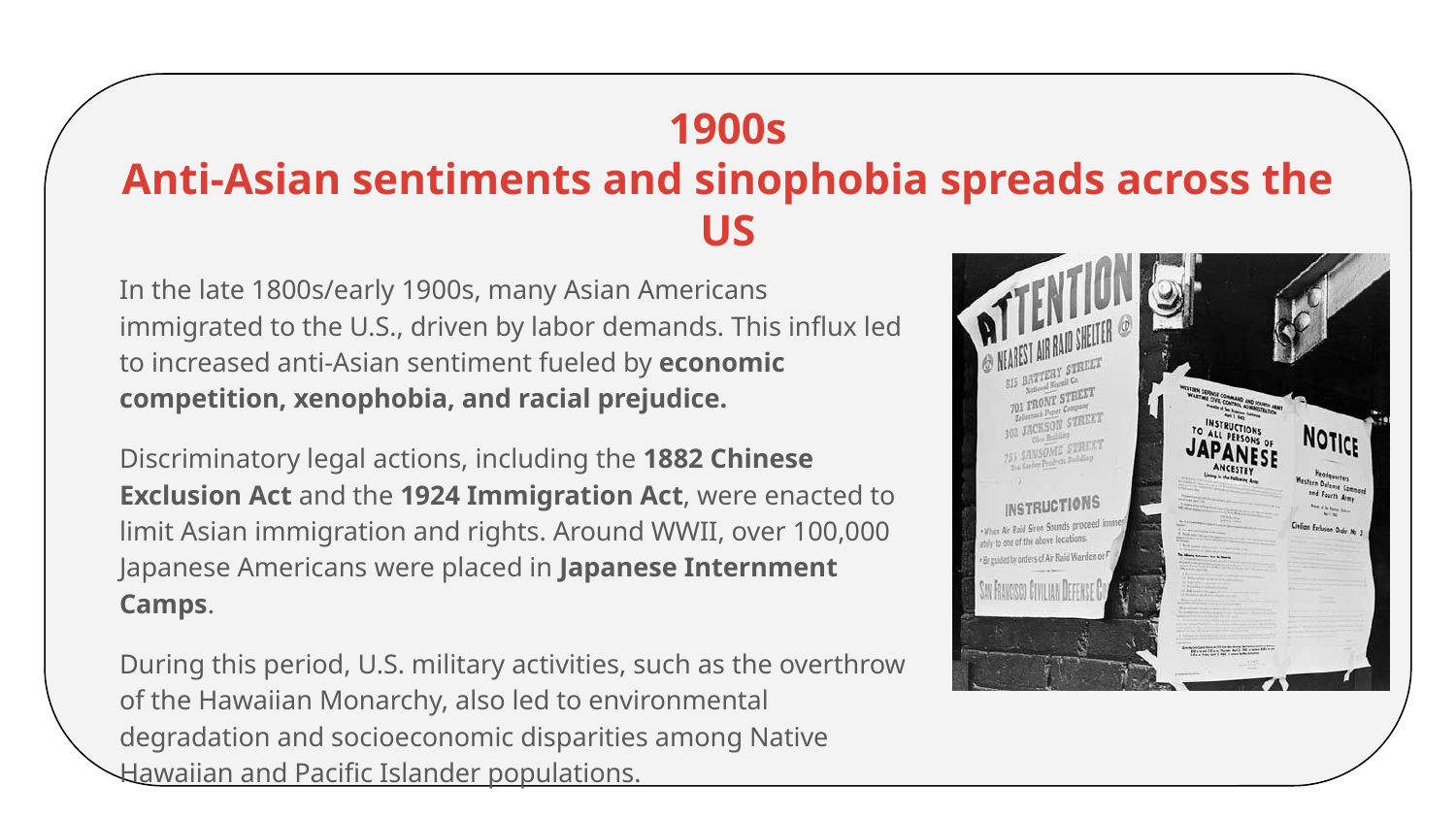

1900s
Anti-Asian sentiments and sinophobia spreads across the US
1870: First additional of a category for people of an Asian Descent in the US Census.
This classification was initially labeled as "Chinese." All individuals of Asian Descent fell into this category.
In 1890, the Census Bureau introduced a category for "Japanese" individuals
In 1920, additional categories were introduced, including, "Filipino," "Korean," and "Hindu" (referring to all South Asians, regardless of religion)
The 2020 US Census included the following categories: “Chinese,” “Japanese,” “Filipino,” “Korean,” “Asian Indian,” “Vietnamese,” and “Other Asian”.
Click on each event to learn more!
In the late 1800s/early 1900s, many Asian Americans immigrated to the U.S., driven by labor demands. This influx led to increased anti-Asian sentiment fueled by economic competition, xenophobia, and racial prejudice.
Discriminatory legal actions, including the 1882 Chinese Exclusion Act and the 1924 Immigration Act, were enacted to limit Asian immigration and rights. Around WWII, over 100,000 Japanese Americans were placed in Japanese Internment Camps.
During this period, U.S. military activities, such as the overthrow of the Hawaiian Monarchy, also led to environmental degradation and socioeconomic disparities among Native Hawaiian and Pacific Islander populations.
First additional of a category for people of an Asian Descent in the US Census.
1870
The Asian American Diaspora begins to grow within the US
1970s
The AANHPI Population is the US continues to rise
2000s
1965
The Immigration and Nationality Act of 1965
1985
The Heckler Report was published
2020
The COVID-19 Pandemic highlights the growing AANHPI healthcare disparities

## Slide 10
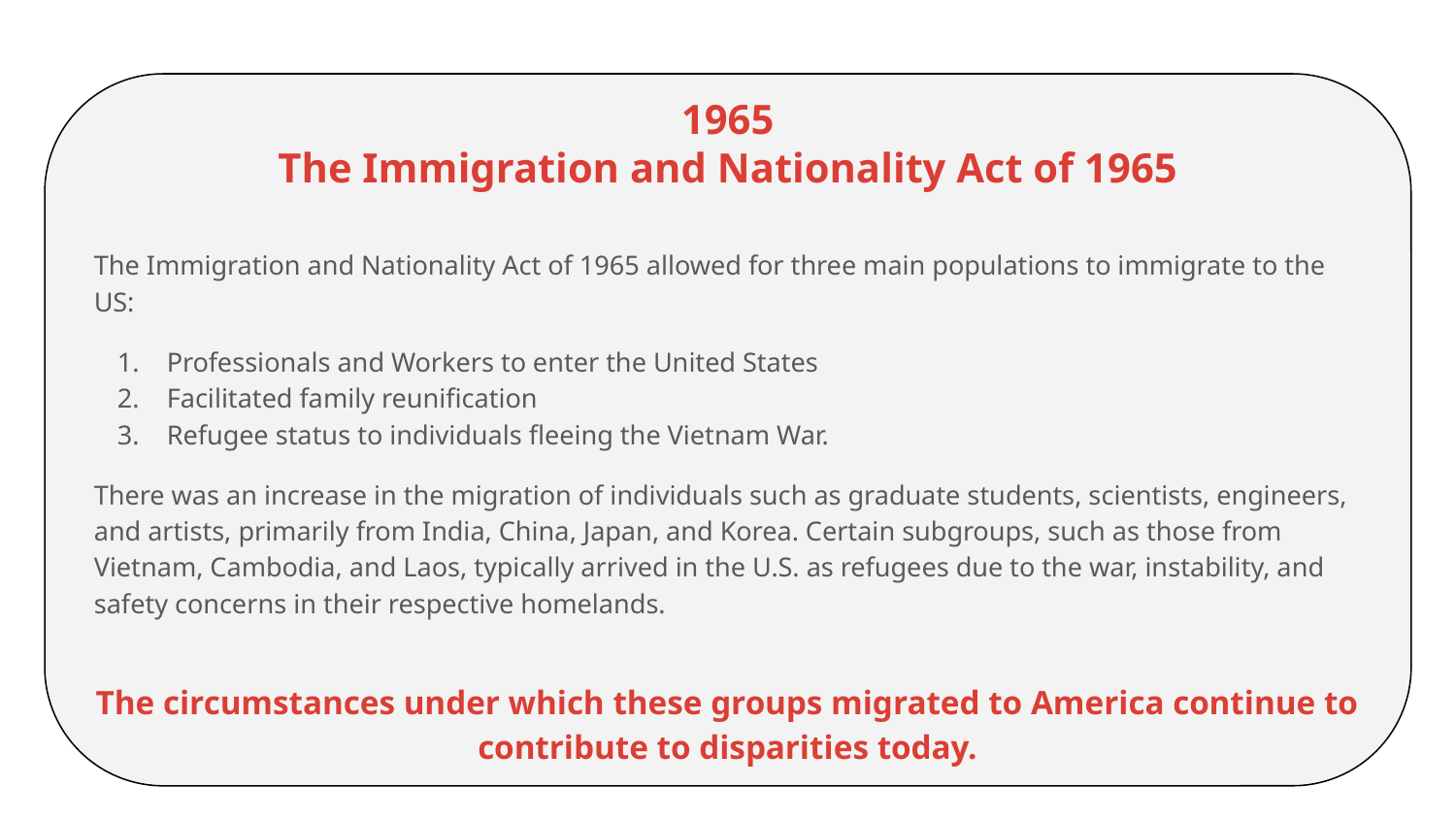

# The Asian Monolith Bias
1965
The Immigration and Nationality Act of 1965
The Immigration and Nationality Act of 1965 allowed for three main populations to immigrate to the US:
Professionals and Workers to enter the United States
Facilitated family reunification
Refugee status to individuals fleeing the Vietnam War.
There was an increase in the migration of individuals such as graduate students, scientists, engineers, and artists, primarily from India, China, Japan, and Korea. Certain subgroups, such as those from Vietnam, Cambodia, and Laos, typically arrived in the U.S. as refugees due to the war, instability, and safety concerns in their respective homelands.
The circumstances under which these groups migrated to America continue to contribute to disparities today.
Click on each event to learn more!
First additional of a category for people of an Asian Descent in the US Census.
1870
The Asian American Diaspora begins to grow within the US
1970s
The AANHPI Population is the US continues to rise
2000s
1965
The Immigration and Nationality Act of 1965
1985
The Heckler Report was published
2020
The COVID-19 Pandemic highlights the growing AANHPI healthcare disparities

## Slide 11
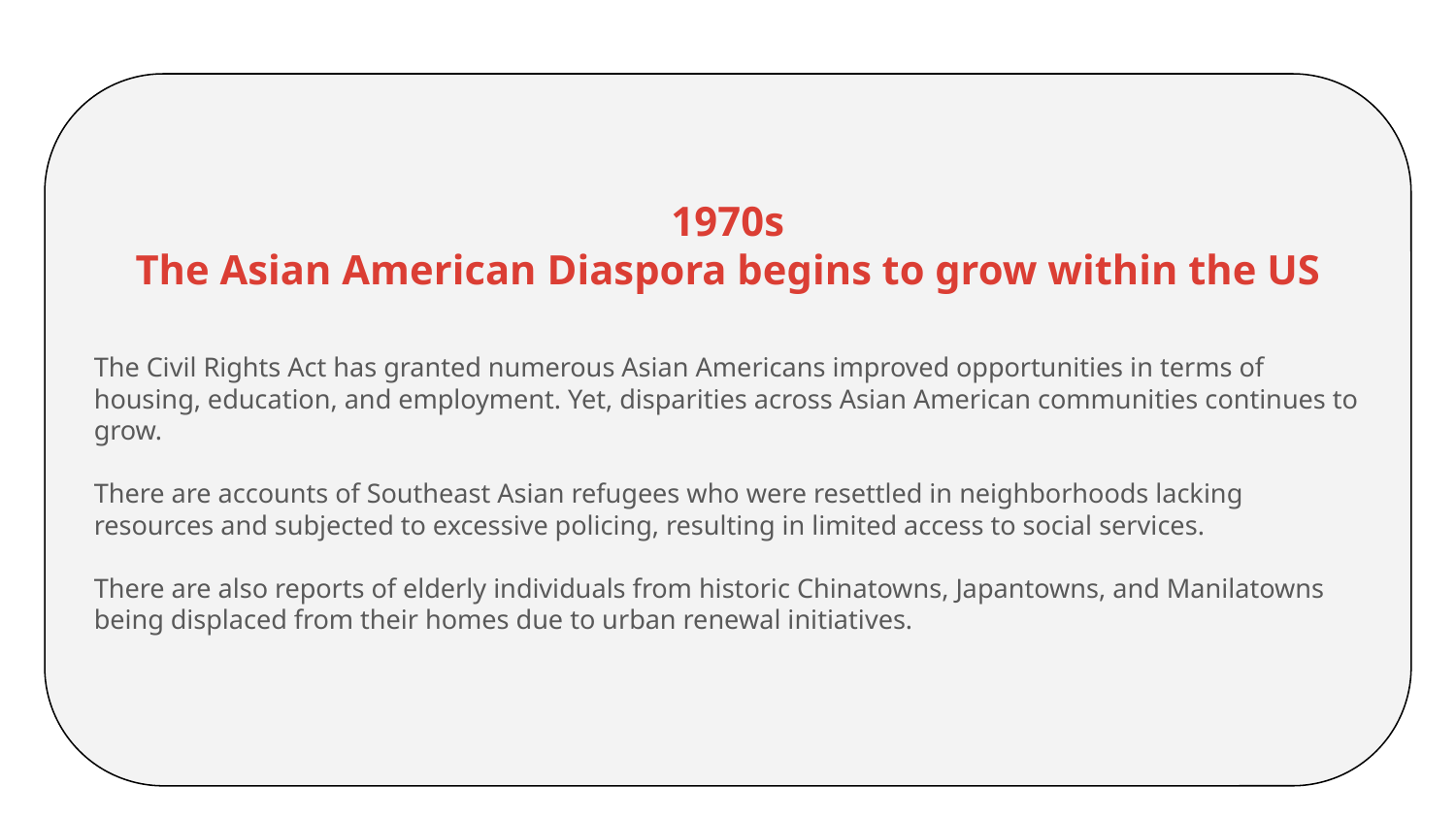

# The Asian Monolith Bias
1970s
The Asian American Diaspora begins to grow within the US
The Civil Rights Act has granted numerous Asian Americans improved opportunities in terms of housing, education, and employment. Yet, disparities across Asian American communities continues to grow.
There are accounts of Southeast Asian refugees who were resettled in neighborhoods lacking resources and subjected to excessive policing, resulting in limited access to social services.
There are also reports of elderly individuals from historic Chinatowns, Japantowns, and Manilatowns being displaced from their homes due to urban renewal initiatives.
Click on each event to learn more!
First additional of a category for people of an Asian Descent in the US Census.
1870
The Asian American Diaspora begins to grow within the US
1970s
The AANHPI Population is the US continues to rise
2000s
1965
The Immigration and Nationality Act of 1965
1985
The Heckler Report was published
2020
The COVID-19 Pandemic highlights the growing AANHPI healthcare disparities

## Slide 12
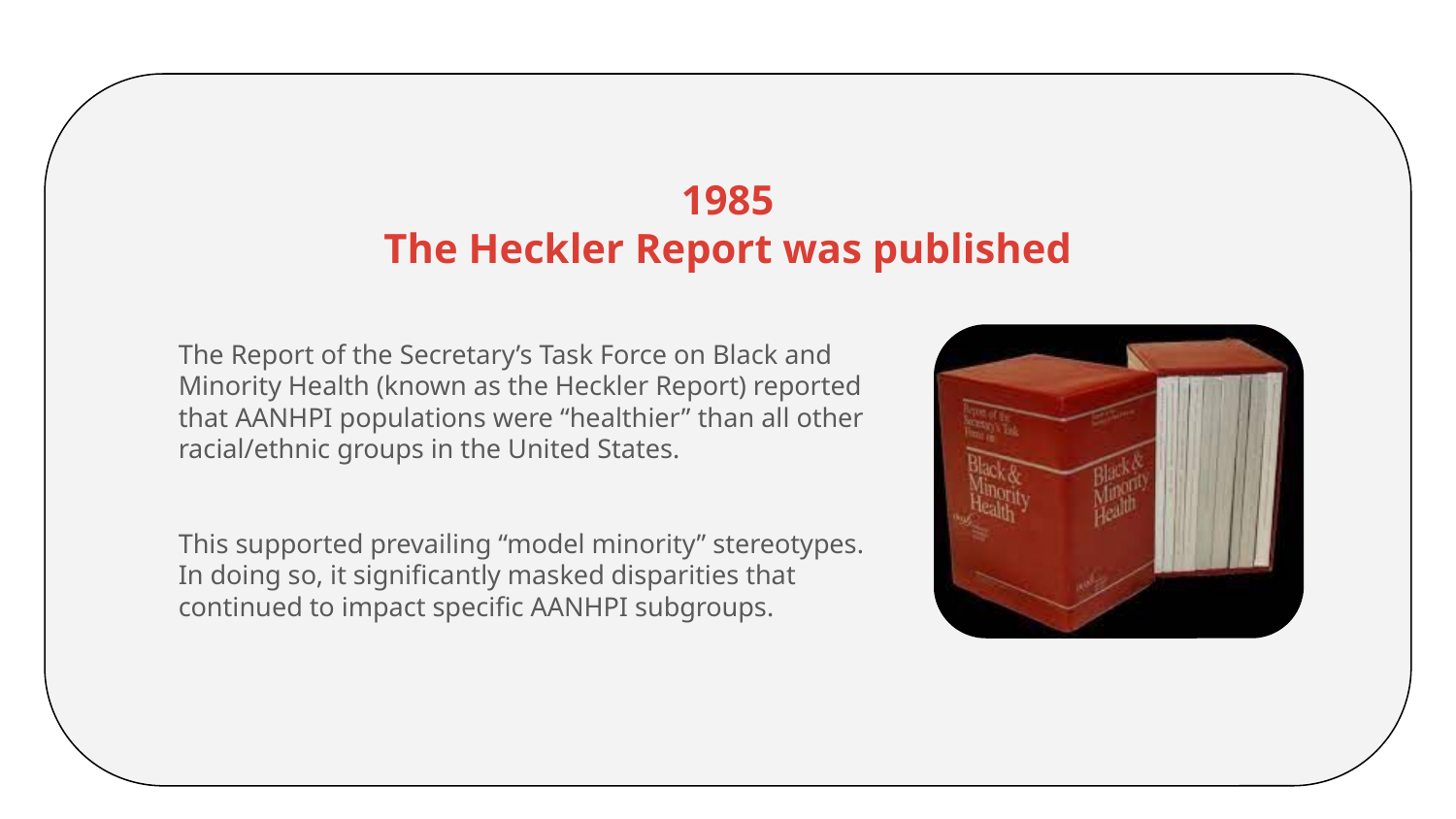

# The Asian Monolith Bias
1985
The Heckler Report was published
Click on each event to learn more!
First additional of a category for people of an Asian Descent in the US Census.
1870
The Report of the Secretary’s Task Force on Black and Minority Health (known as the Heckler Report) reported that AANHPI populations were “healthier” than all other racial/ethnic groups in the United States.
This supported prevailing “model minority” stereotypes. In doing so, it significantly masked disparities that continued to impact specific AANHPI subgroups.
The Asian American Diaspora begins to grow within the US
1970s
The AANHPI Population is the US continues to rise
2000s
1965
The Immigration and Nationality Act of 1965
1985
The Heckler Report was published
2020
The COVID-19 Pandemic highlights the growing AANHPI healthcare disparities

## Slide 13
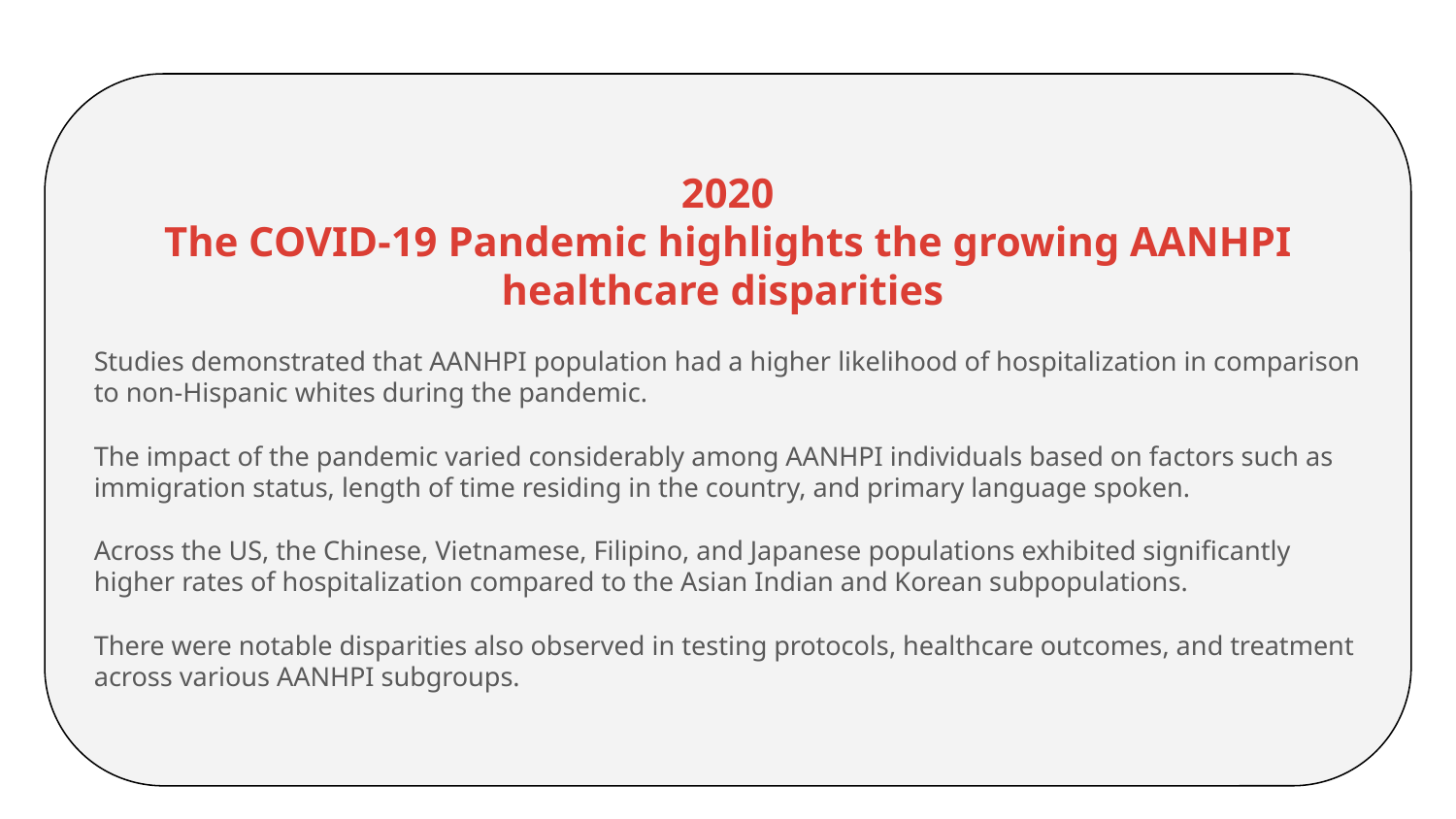

# The Asian Monolith Bias
2020
The COVID-19 Pandemic highlights the growing AANHPI healthcare disparities
Studies demonstrated that AANHPI population had a higher likelihood of hospitalization in comparison to non-Hispanic whites during the pandemic.
The impact of the pandemic varied considerably among AANHPI individuals based on factors such as immigration status, length of time residing in the country, and primary language spoken.
Across the US, the Chinese, Vietnamese, Filipino, and Japanese populations exhibited significantly higher rates of hospitalization compared to the Asian Indian and Korean subpopulations.
There were notable disparities also observed in testing protocols, healthcare outcomes, and treatment across various AANHPI subgroups.
Click on each event to learn more!
First additional of a category for people of an Asian Descent in the US Census.
1870
The Asian American Diaspora begins to grow within the US
1970s
The AANHPI Population is the US continues to rise
2000s
1965
The Immigration and Nationality Act of 1965
1985
The Heckler Report was published
2020
The COVID-19 Pandemic highlights the growing AANHPI healthcare disparities

## Slide 14
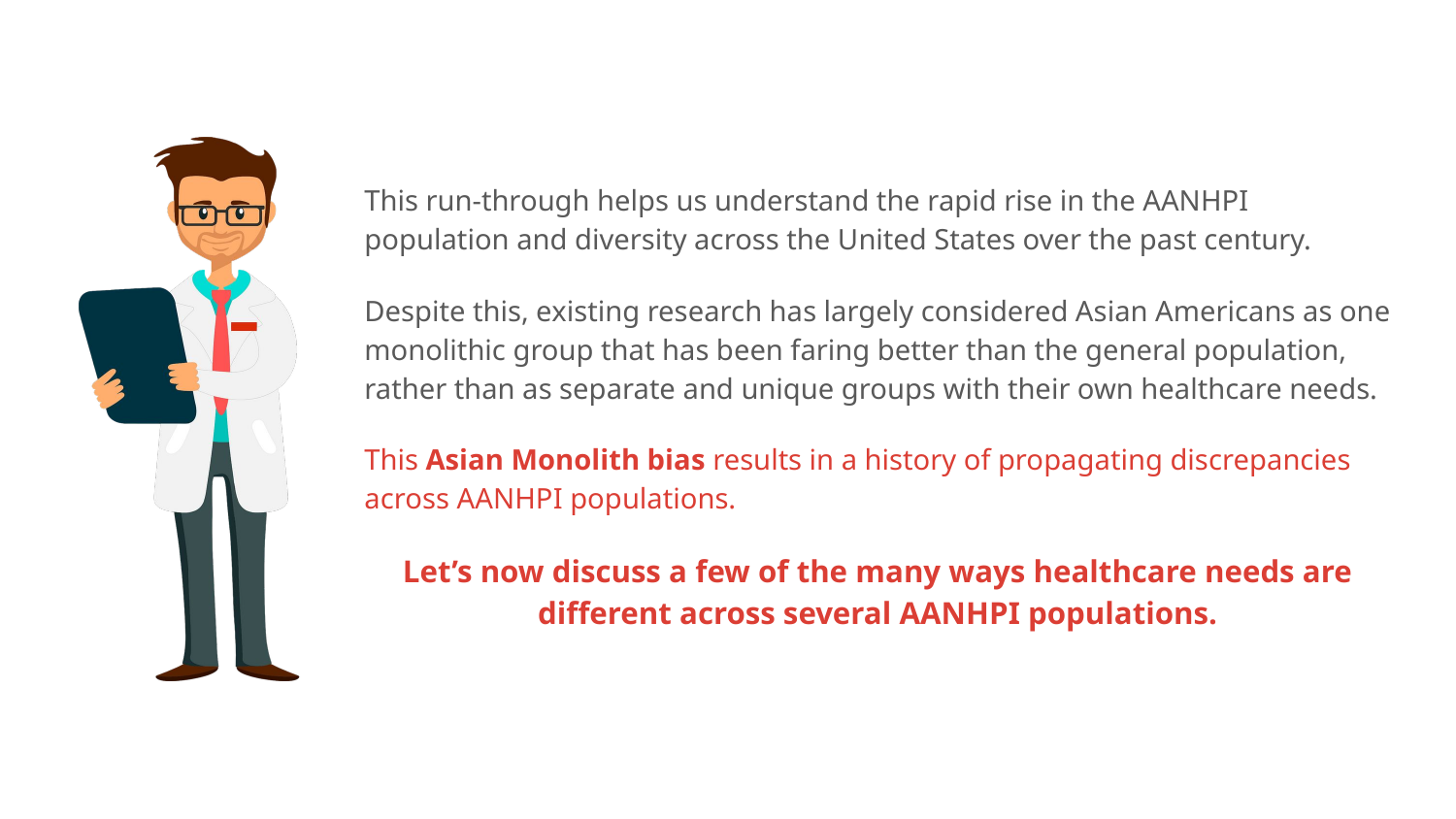

This run-through helps us understand the rapid rise in the AANHPI population and diversity across the United States over the past century.
Despite this, existing research has largely considered Asian Americans as one monolithic group that has been faring better than the general population, rather than as separate and unique groups with their own healthcare needs.
This Asian Monolith bias results in a history of propagating discrepancies across AANHPI populations.
Let’s now discuss a few of the many ways healthcare needs are different across several AANHPI populations.

## Slide 15
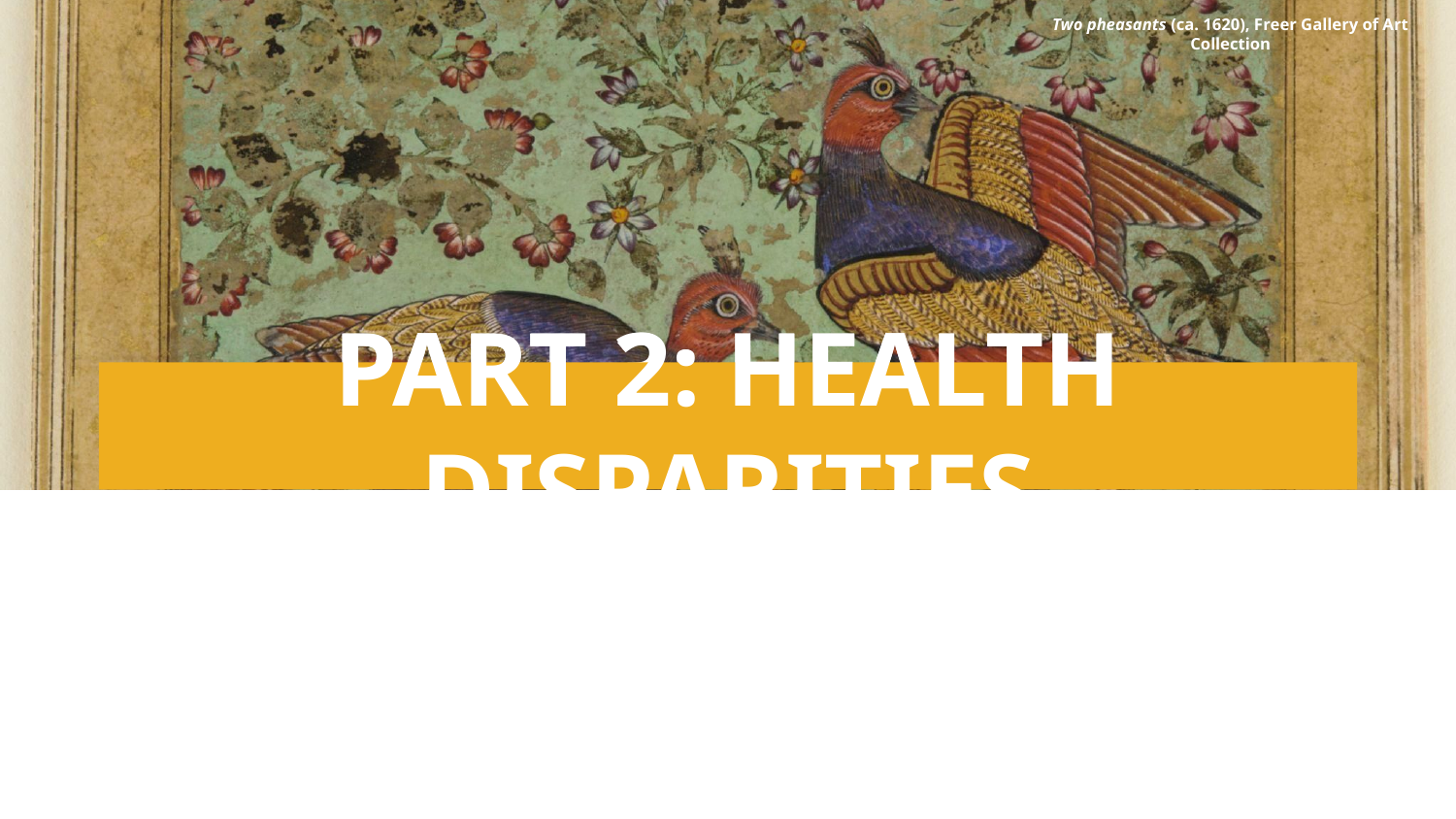

Two pheasants (ca. 1620), Freer Gallery of Art Collection
PART 3: HEALTH DISPARITIES
PART 2: HEALTH DISPARITIES

## Slide 16
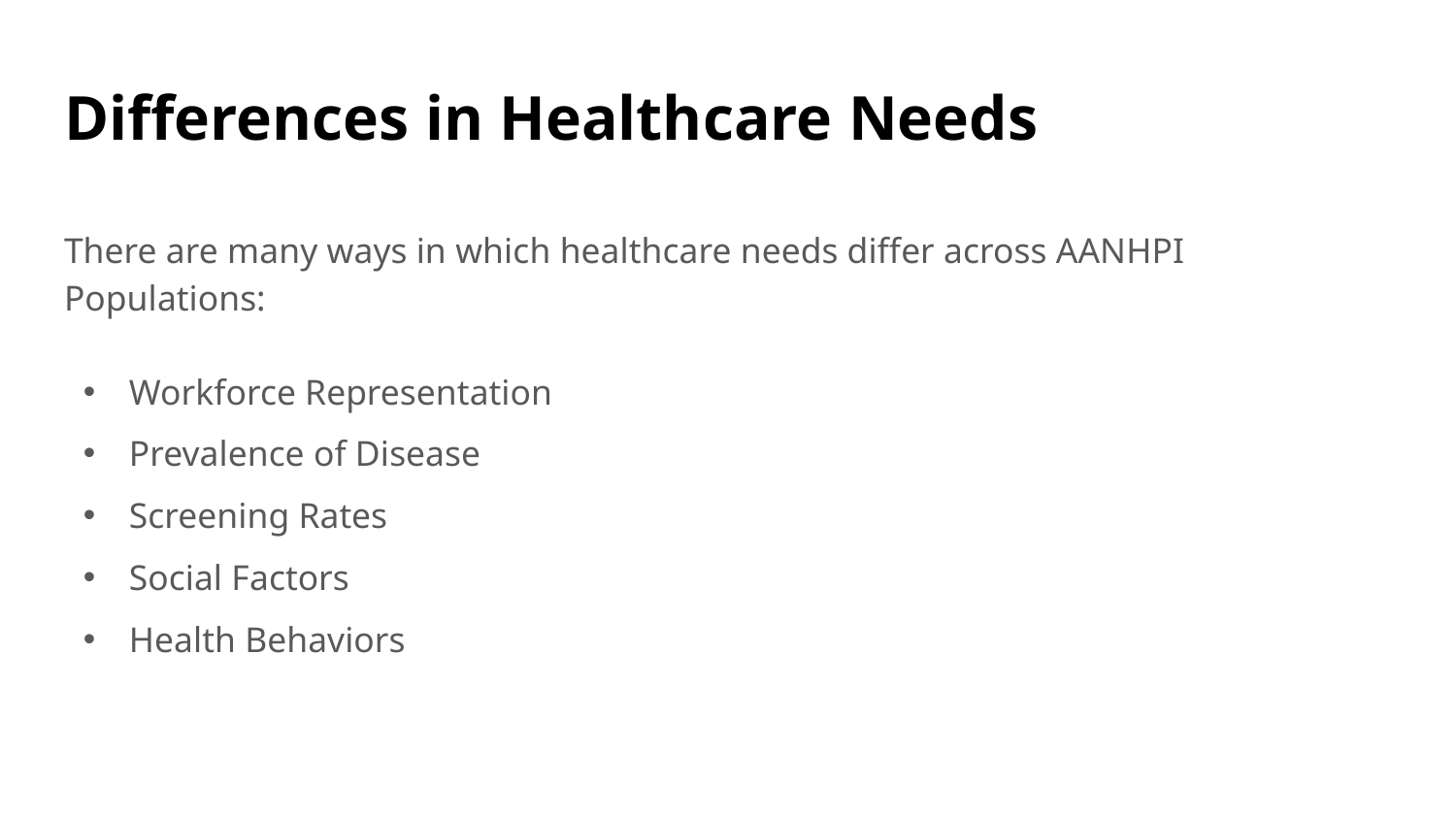

# Differences in Healthcare Needs
There are many ways in which healthcare needs differ across AANHPI Populations:
Workforce Representation
Prevalence of Disease
Screening Rates
Social Factors
Health Behaviors

## Slide 17
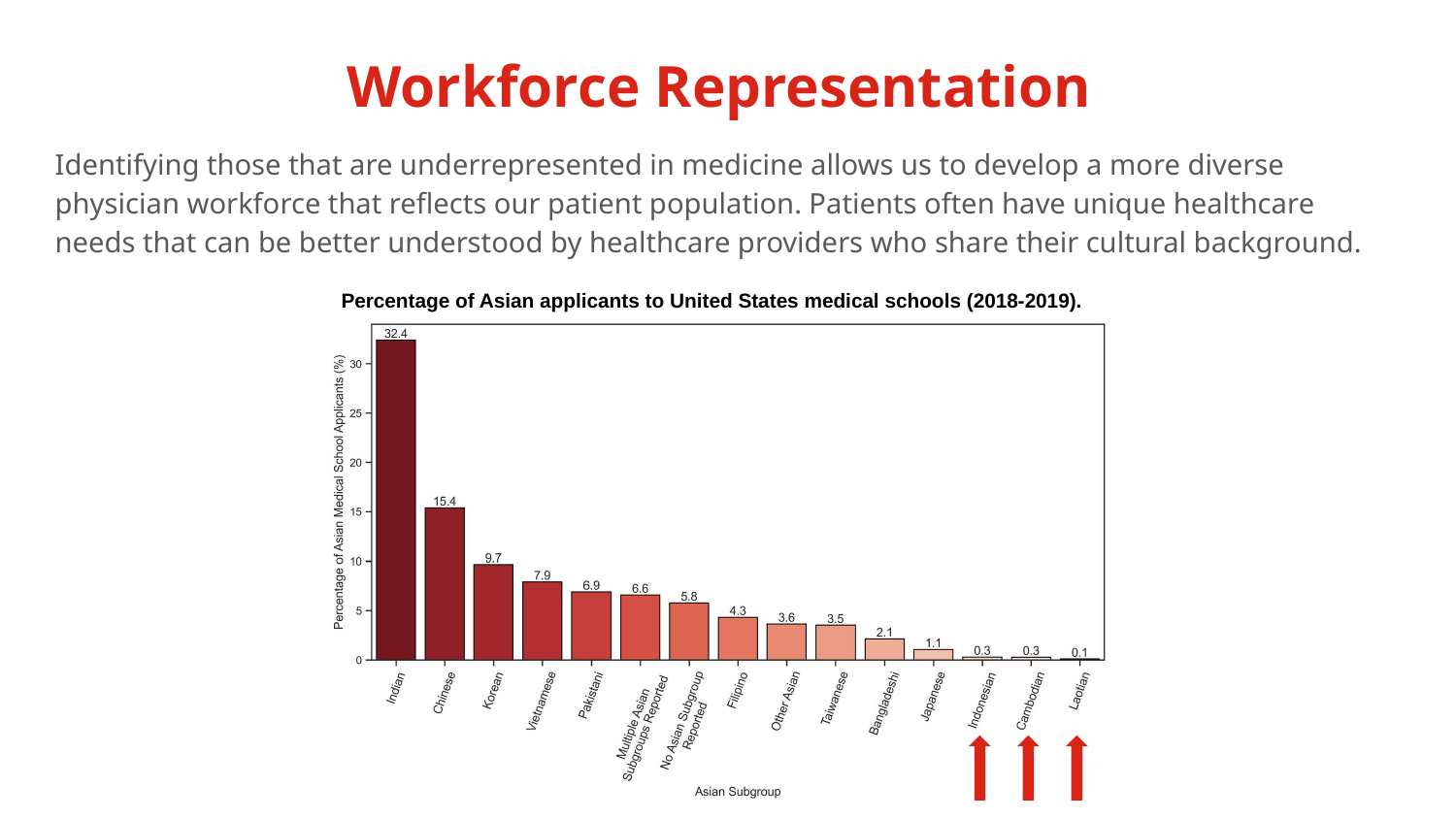

# Workforce Representation
Identifying those that are underrepresented in medicine allows us to develop a more diverse physician workforce that reflects our patient population. Patients often have unique healthcare needs that can be better understood by healthcare providers who share their cultural background.
Percentage of Asian applicants to United States medical schools (2018-2019).

## Slide 18
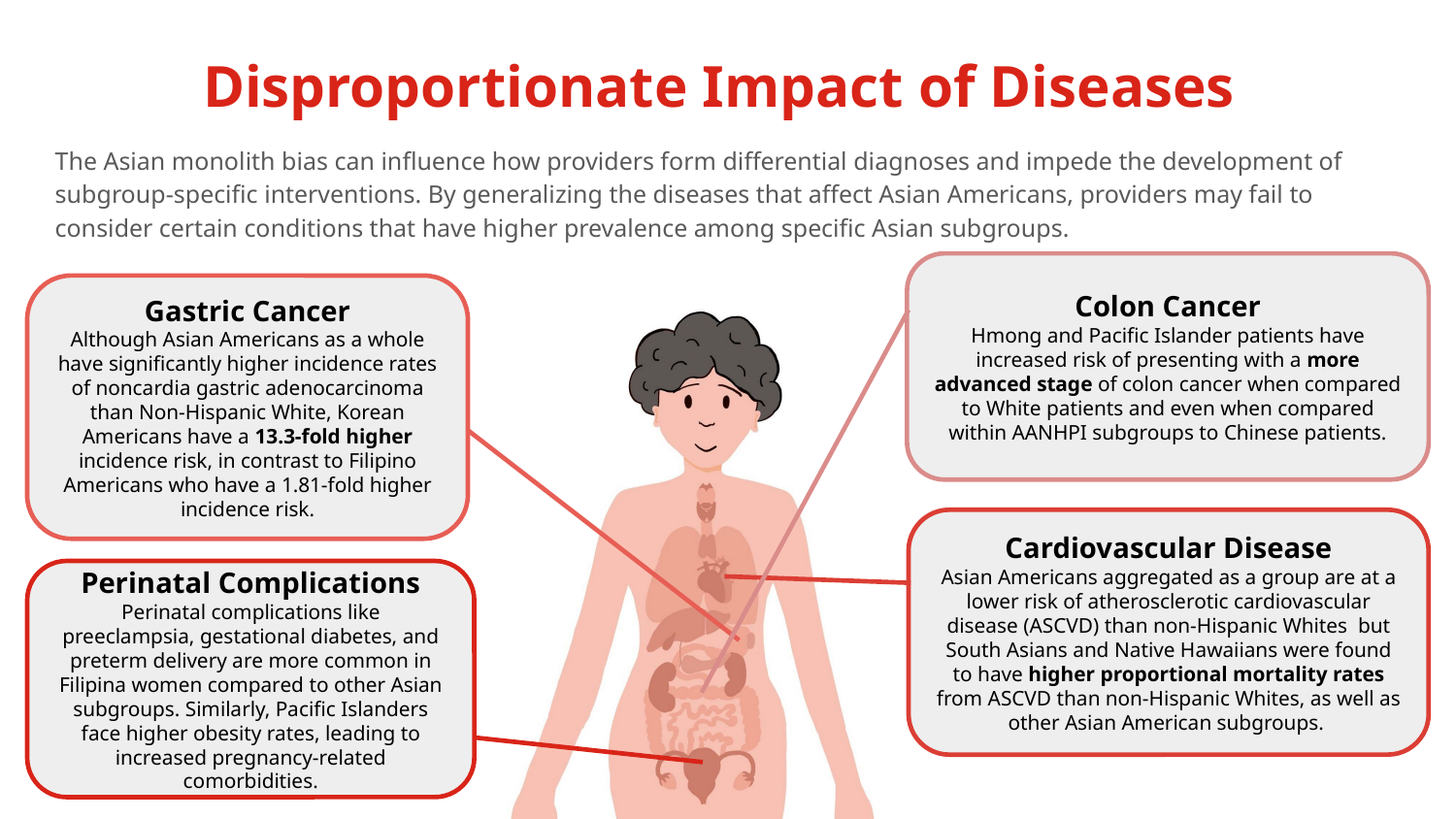

# Disproportionate Impact of Diseases
The Asian monolith bias can influence how providers form differential diagnoses and impede the development of subgroup-specific interventions. By generalizing the diseases that affect Asian Americans, providers may fail to consider certain conditions that have higher prevalence among specific Asian subgroups.
Colon Cancer
Hmong and Pacific Islander patients have increased risk of presenting with a more advanced stage of colon cancer when compared to White patients and even when compared within AANHPI subgroups to Chinese patients.
Gastric Cancer
Although Asian Americans as a whole have significantly higher incidence rates of noncardia gastric adenocarcinoma than Non-Hispanic White, Korean Americans have a 13.3-fold higher incidence risk, in contrast to Filipino Americans who have a 1.81-fold higher incidence risk.
Cardiovascular Disease
Asian Americans aggregated as a group are at a lower risk of atherosclerotic cardiovascular disease (ASCVD) than non-Hispanic Whites but South Asians and Native Hawaiians were found to have higher proportional mortality rates from ASCVD than non-Hispanic Whites, as well as other Asian American subgroups.
Perinatal Complications
Perinatal complications like preeclampsia, gestational diabetes, and preterm delivery are more common in Filipina women compared to other Asian subgroups. Similarly, Pacific Islanders face higher obesity rates, leading to increased pregnancy-related comorbidities.

## Slide 19
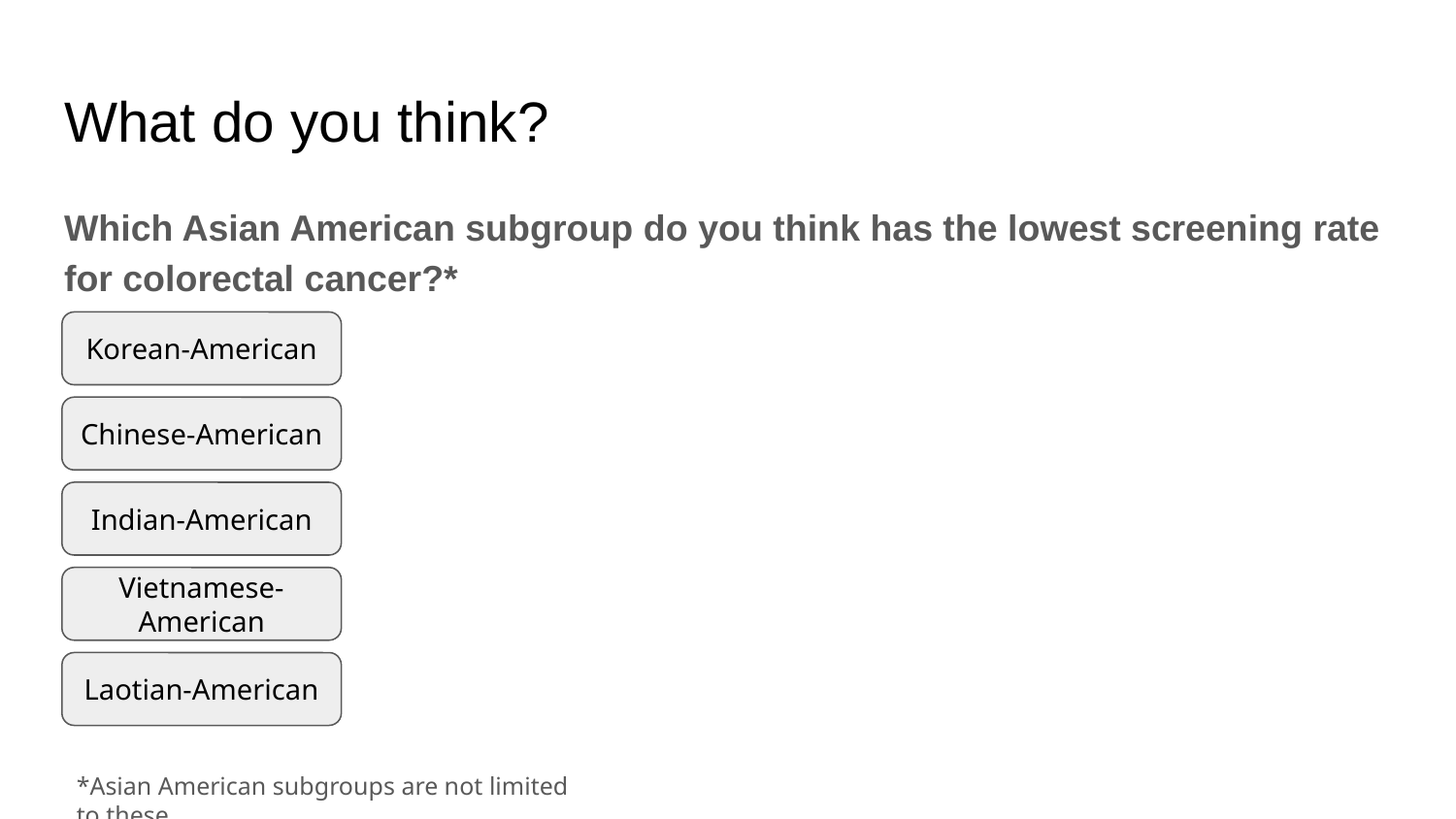

# What do you think?
Which Asian American subgroup do you think has the lowest screening rate for colorectal cancer?*
Korean-American
Chinese-American
Indian-American
Vietnamese-American
Laotian-American
*Asian American subgroups are not limited to these

## Slide 20
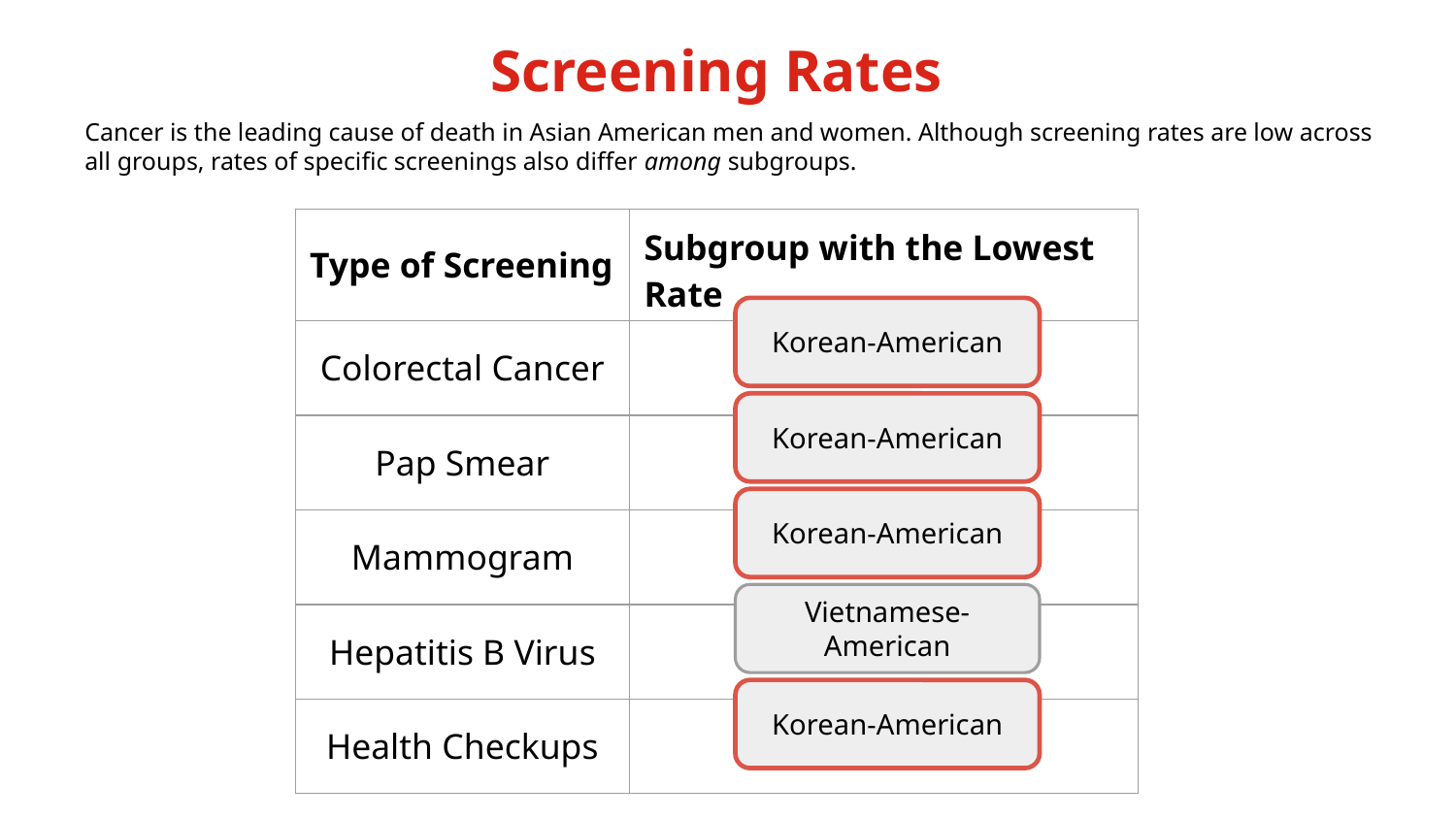

# Screening Rates
Cancer is the leading cause of death in Asian American men and women. Although screening rates are low across all groups, rates of specific screenings also differ among subgroups.
| Type of Screening | Subgroup with the Lowest Rate |
| --- | --- |
| Colorectal Cancer | |
| Pap Smear | |
| Mammogram | |
| Hepatitis B Virus | |
| Health Checkups | |
Korean-American
Korean-American
Korean-American
Vietnamese-American
Korean-American

## Slide 21
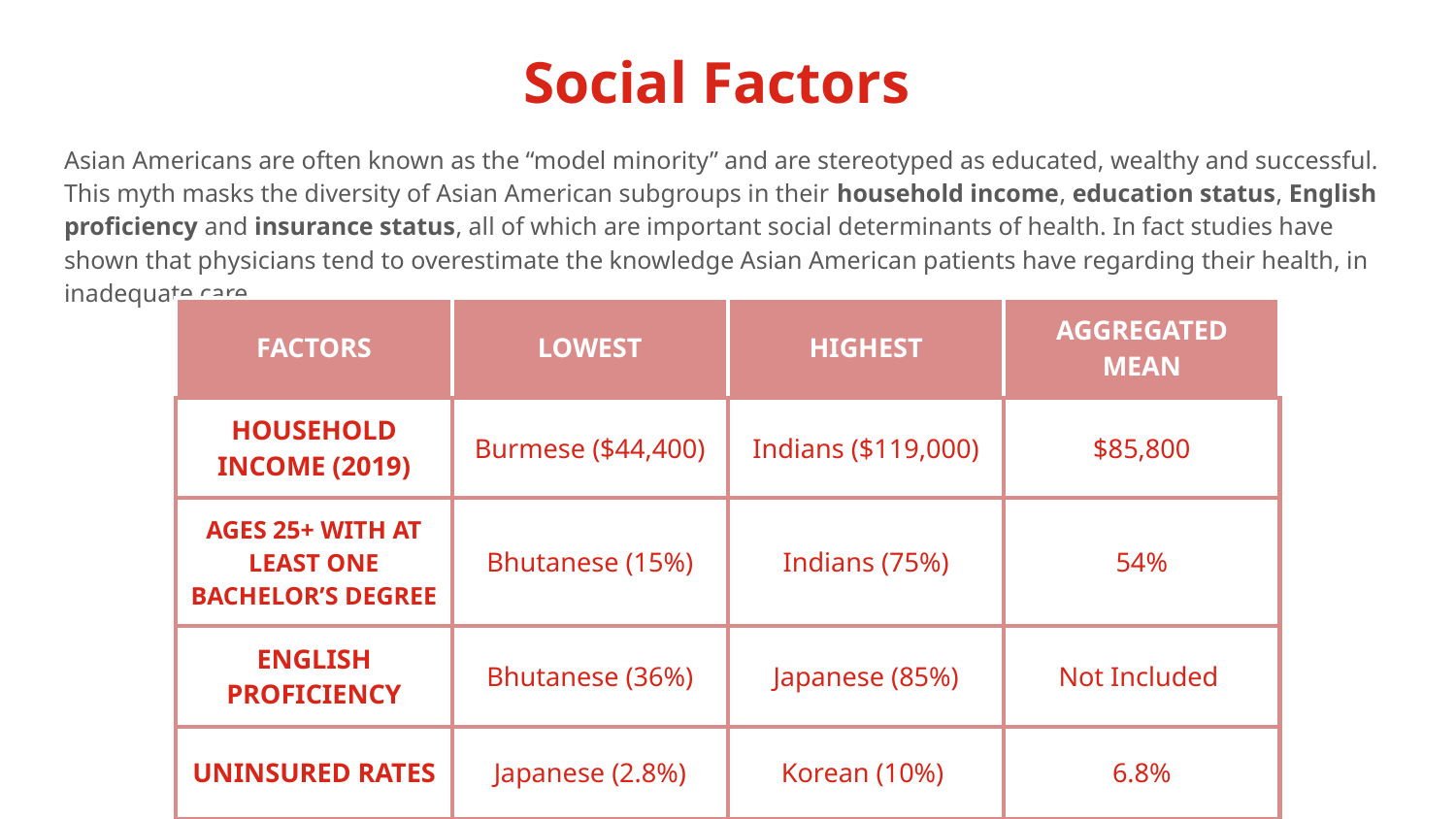

# Social Factors
Asian Americans are often known as the “model minority” and are stereotyped as educated, wealthy and successful. This myth masks the diversity of Asian American subgroups in their household income, education status, English proficiency and insurance status, all of which are important social determinants of health. In fact studies have shown that physicians tend to overestimate the knowledge Asian American patients have regarding their health, in inadequate care.
| FACTORS | LOWEST | HIGHEST | AGGREGATED MEAN |
| --- | --- | --- | --- |
| HOUSEHOLD INCOME (2019) | Burmese ($44,400) | Indians ($119,000) | $85,800 |
| AGES 25+ WITH AT LEAST ONE BACHELOR’S DEGREE | Bhutanese (15%) | Indians (75%) | 54% |
| ENGLISH PROFICIENCY | Bhutanese (36%) | Japanese (85%) | Not Included |
| UNINSURED RATES | Japanese (2.8%) | Korean (10%) | 6.8% |

## Slide 22
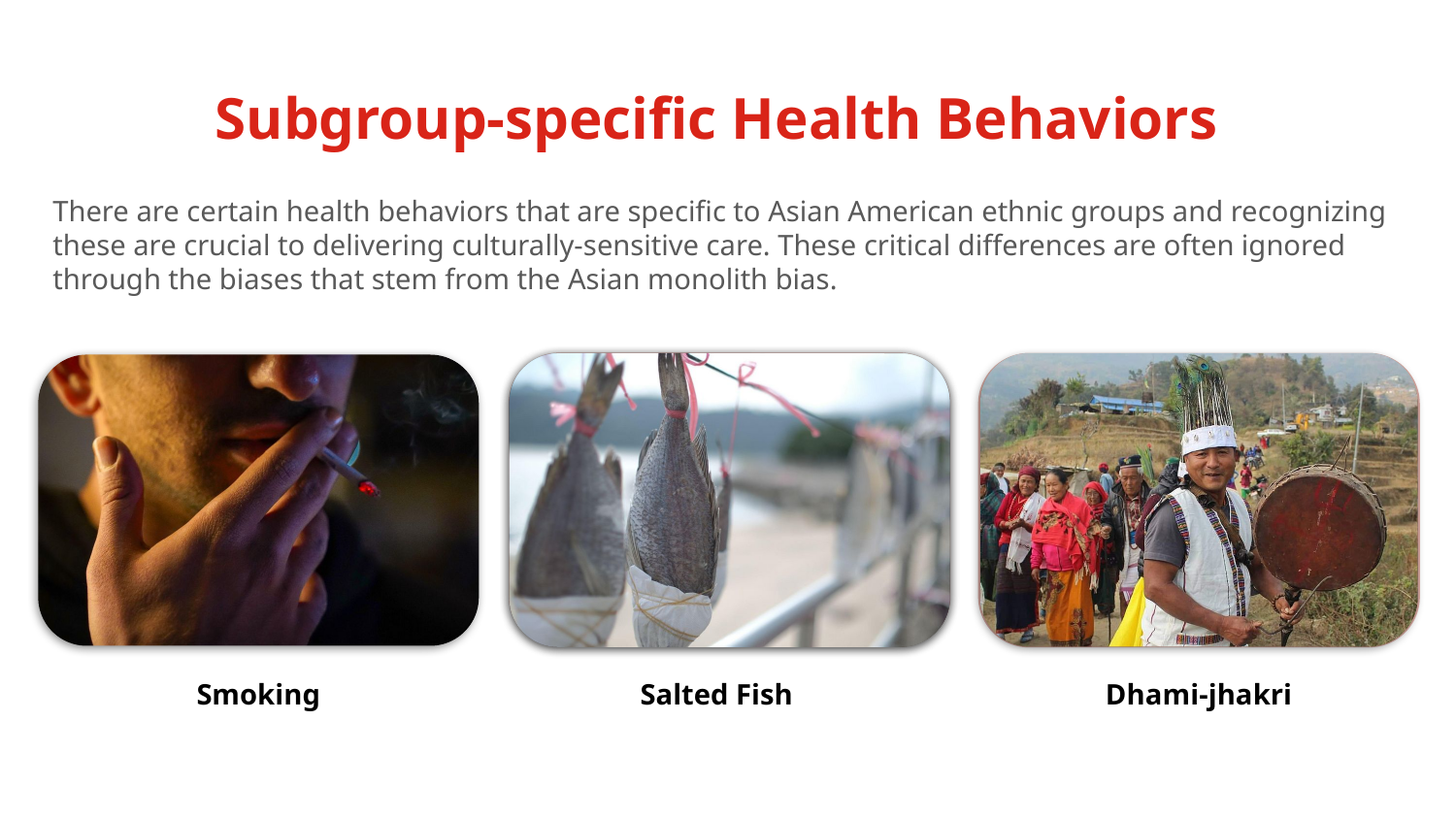

# Subgroup-specific Health Behaviors
There are certain health behaviors that are specific to Asian American ethnic groups and recognizing these are crucial to delivering culturally-sensitive care. These critical differences are often ignored through the biases that stem from the Asian monolith bias.
Chinese-style salted fish is popular among Southern Chinese and Southeastern Asian populations and studies have demonstrated its consumption is associated with increased risk of nasopharyngeal cancer and stomach cancer.
 Providers may omit dietary counseling with Asian American patients due to failure to account for differences in diets.
Traditional healers like dhami-jakhri play a huge role in health care for resettled Bhutanese refugees. Encouragement may be needed for Bhutanese patients to feel more comfortable sharing their traditional treatments and practices with their providers.
Smoking
Salted Fish
Dhami-jhakri

## Slide 23
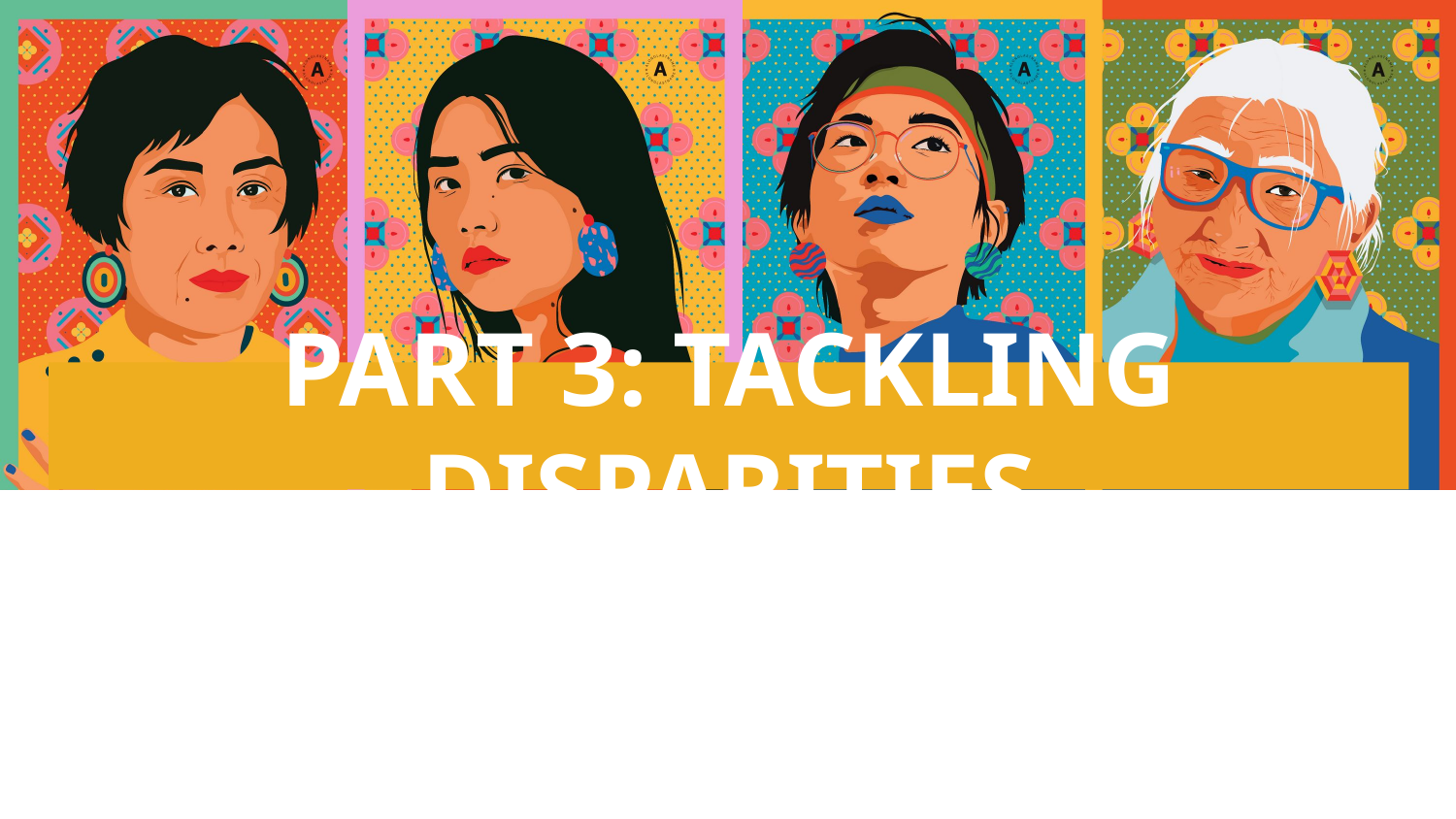

“I Still Believe in Our City”, Amanda Phingbodhipakkiya
PART 3: HEALTH DISPARITIES
PART 3: TACKLING DISPARITIES

## Slide 24
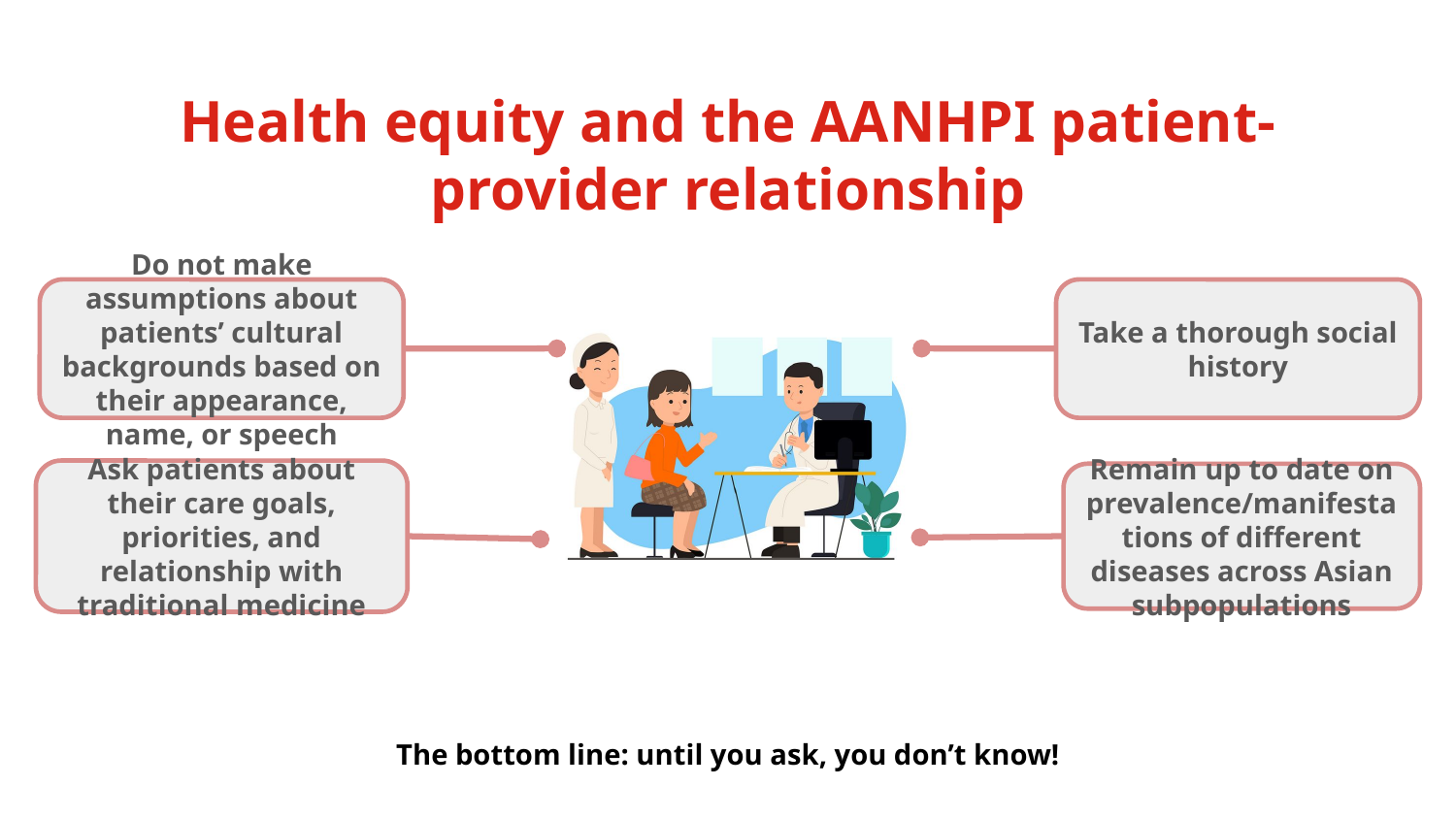

# Health equity and the AANHPI patient-provider relationship
Do not make assumptions about patients’ cultural backgrounds based on their appearance, name, or speech
Take a thorough social history
Ask patients about their care goals, priorities, and relationship with traditional medicine
Remain up to date on prevalence/manifestations of different diseases across Asian subpopulations
The bottom line: until you ask, you don’t know!

## Slide 25
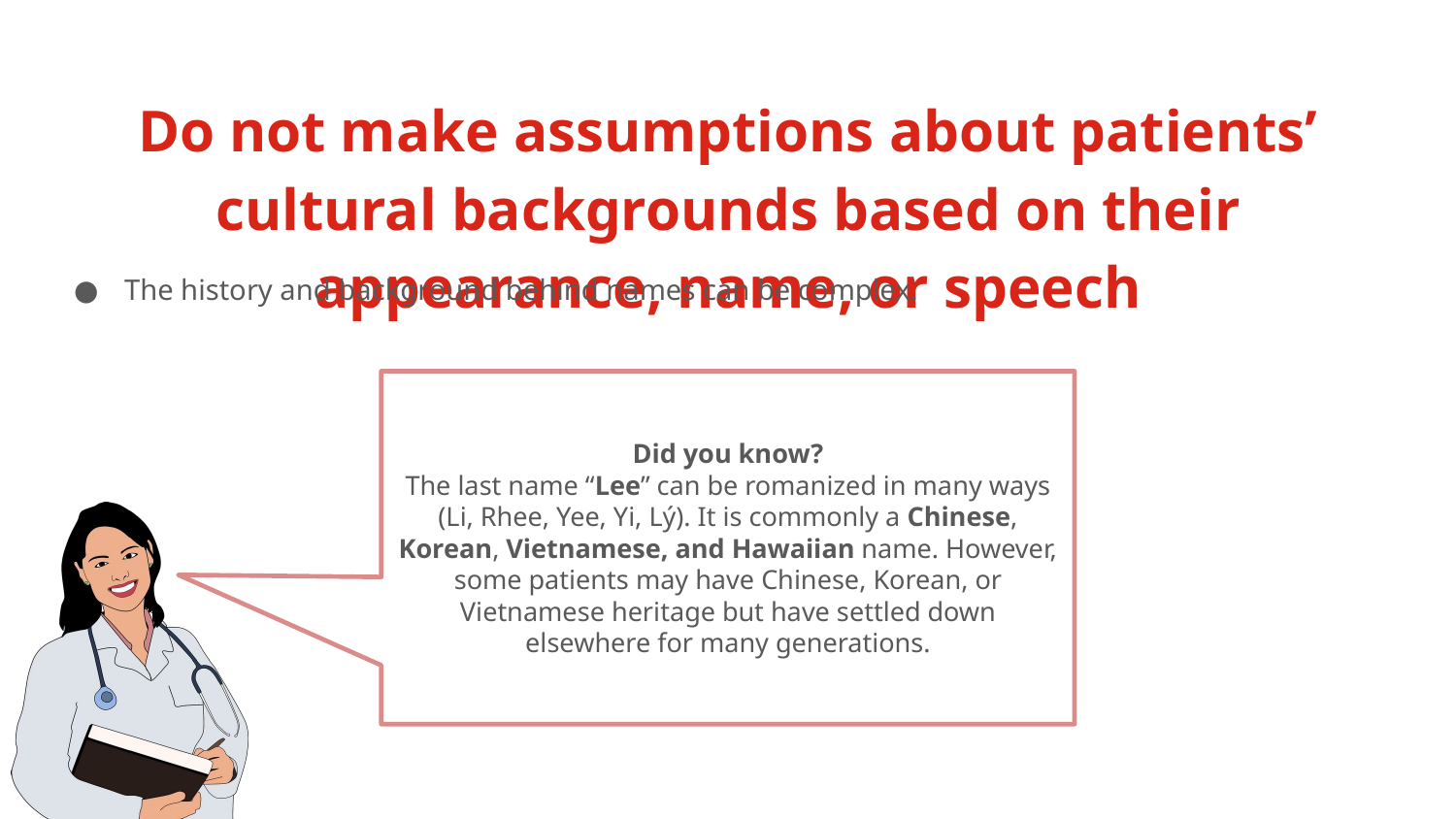

# Do not make assumptions about patients’ cultural backgrounds based on their appearance, name, or speech
The history and background behind names can be complex.
Did you know?
The last name “Lee” can be romanized in many ways (Li, Rhee, Yee, Yi, Lý). It is commonly a Chinese, Korean, Vietnamese, and Hawaiian name. However, some patients may have Chinese, Korean, or Vietnamese heritage but have settled down elsewhere for many generations.

## Slide 26
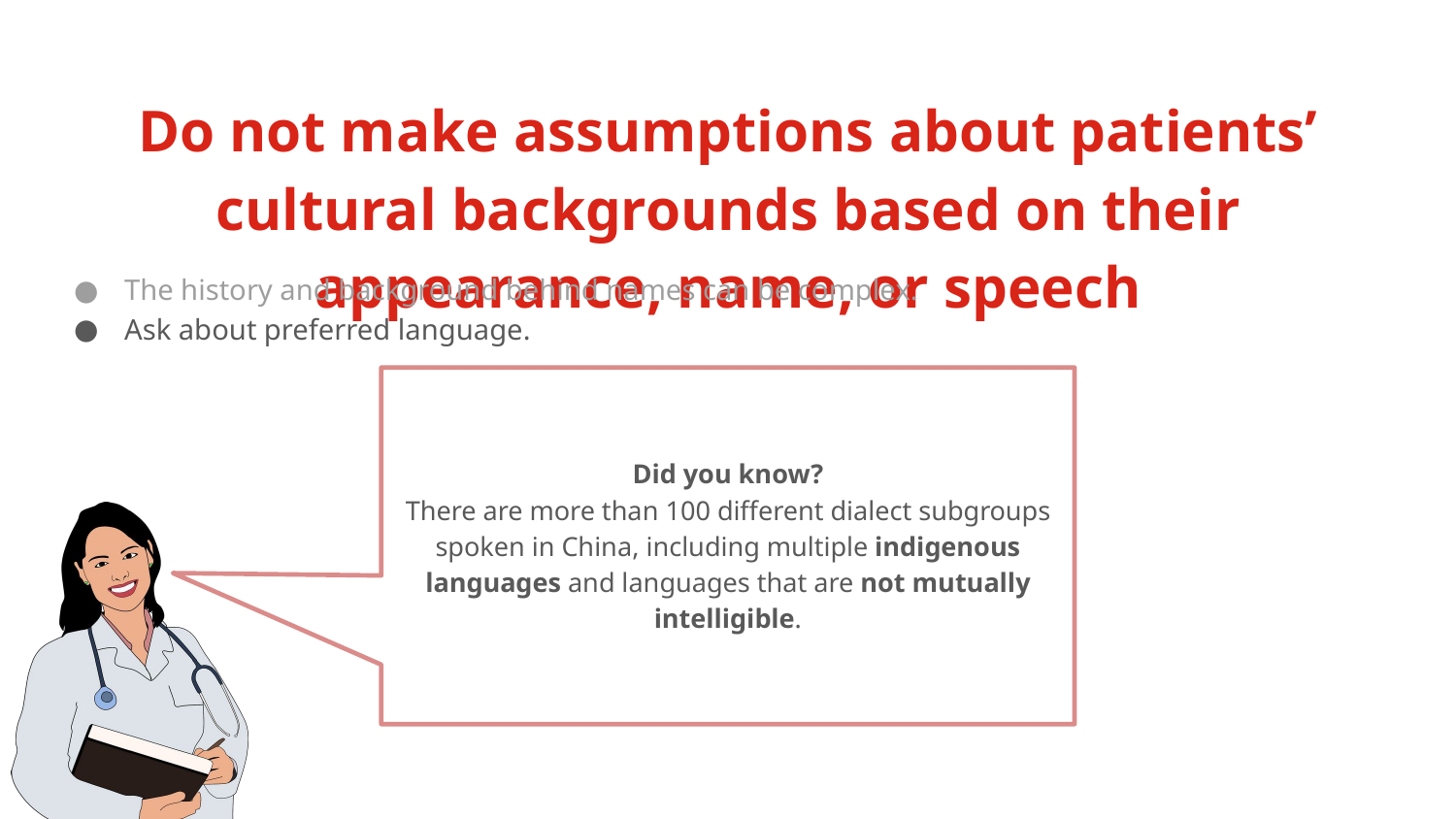

# Do not make assumptions about patients’ cultural backgrounds based on their appearance, name, or speech
The history and background behind names can be complex.
Ask about preferred language.
Did you know?
There are more than 100 different dialect subgroups spoken in China, including multiple indigenous languages and languages that are not mutually intelligible.

## Slide 27
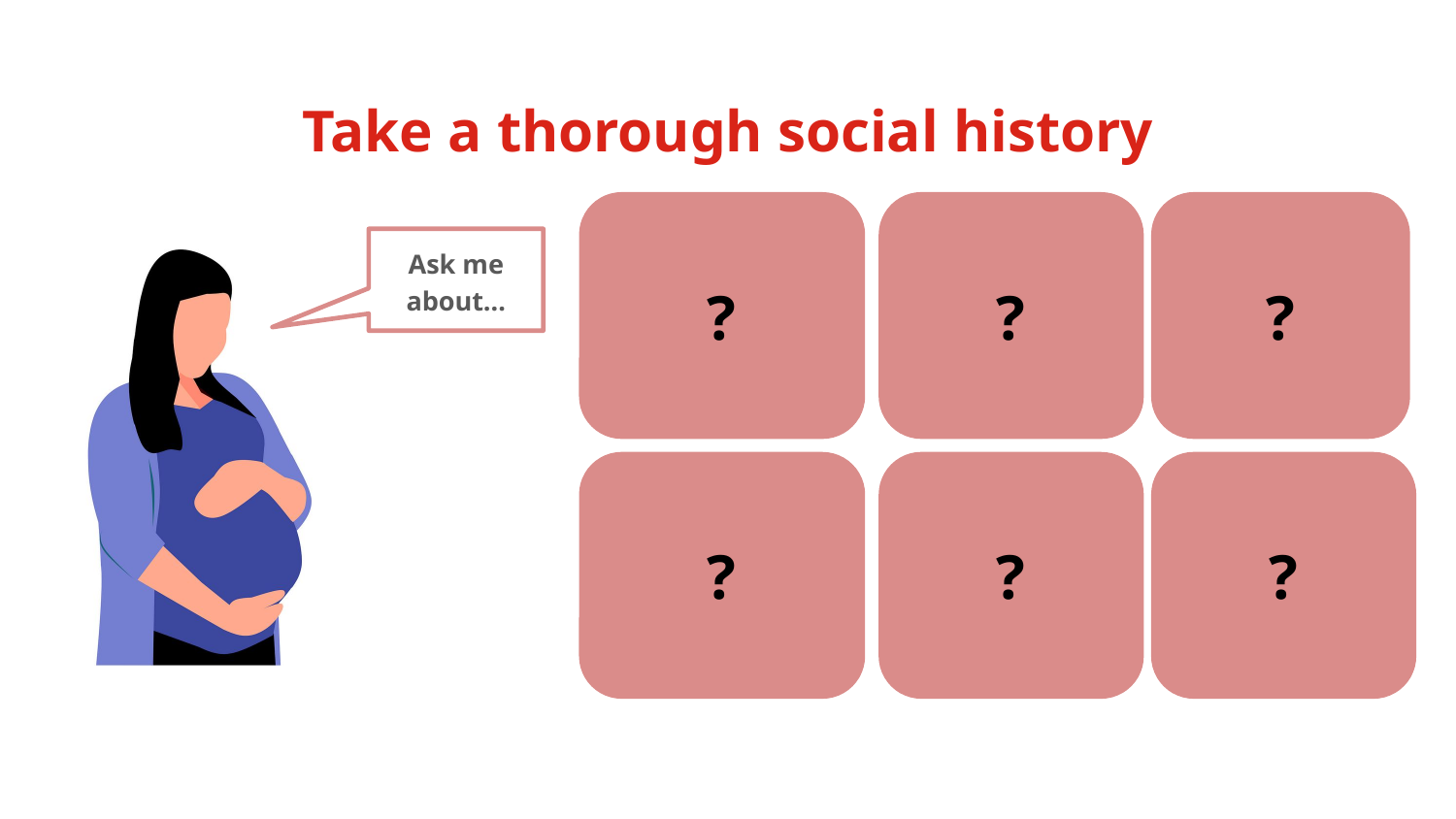

# Take a thorough social history
?
?
?
Ask me about…
?
?
?

## Slide 28
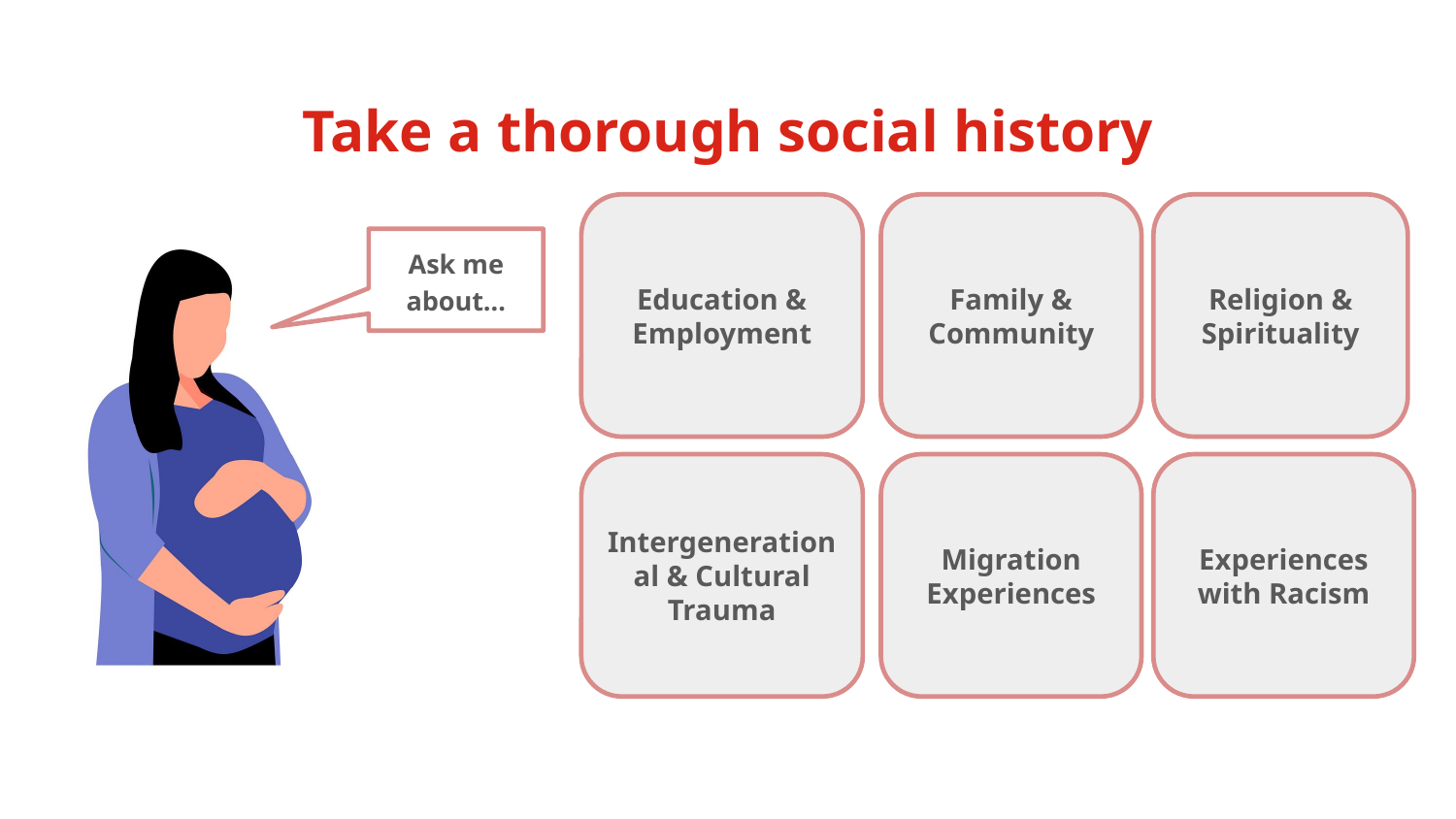

# Take a thorough social history
Family & Community
Religion & Spirituality
Education & Employment
Ask me about…
Intergenerational & Cultural Trauma
Migration Experiences
Experiences with Racism

## Slide 29
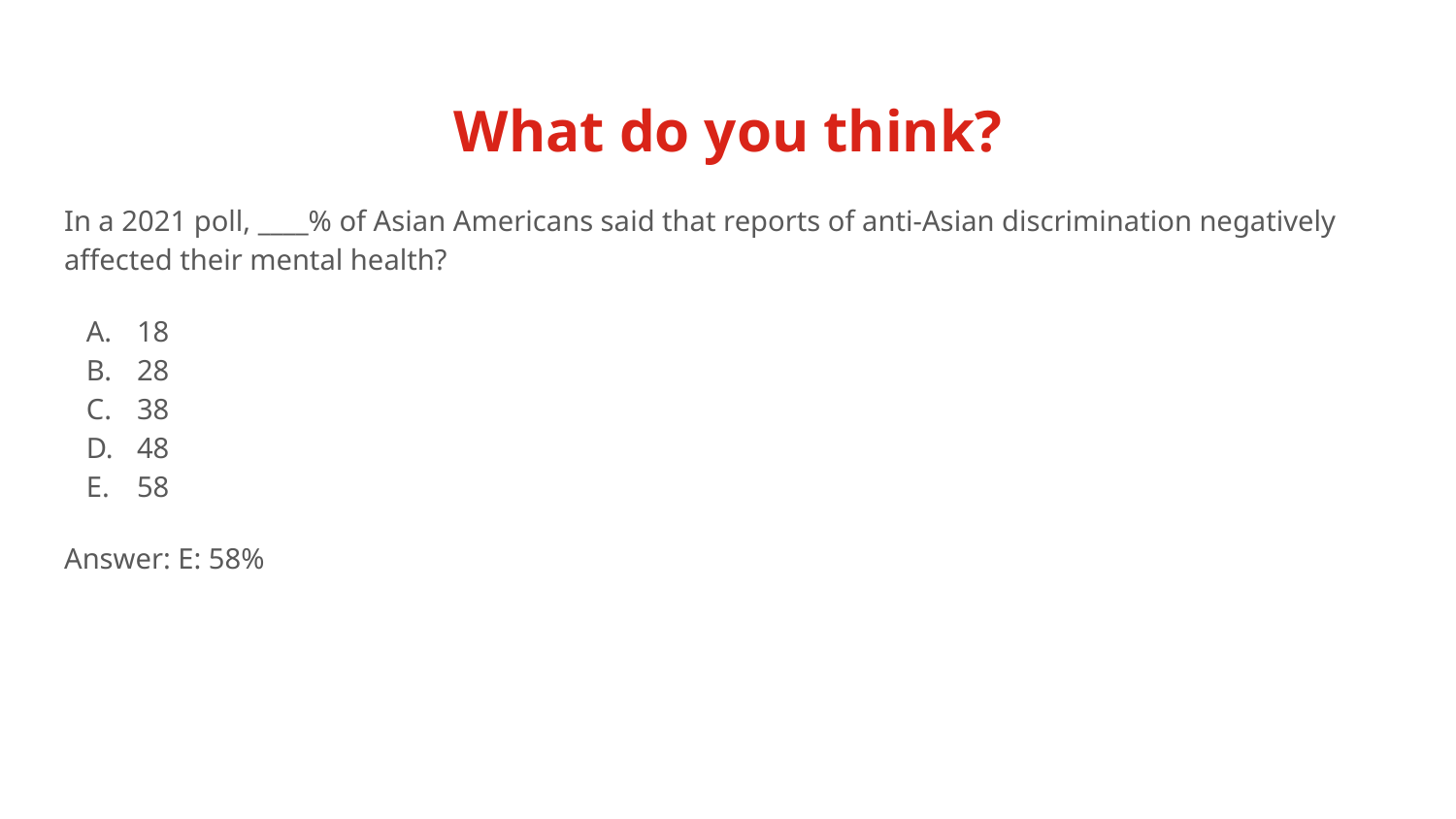

# What do you think?
In a 2021 poll, ____% of Asian Americans said that reports of anti-Asian discrimination negatively affected their mental health?
18
28
38
48
58
Answer: E: 58%

## Slide 30
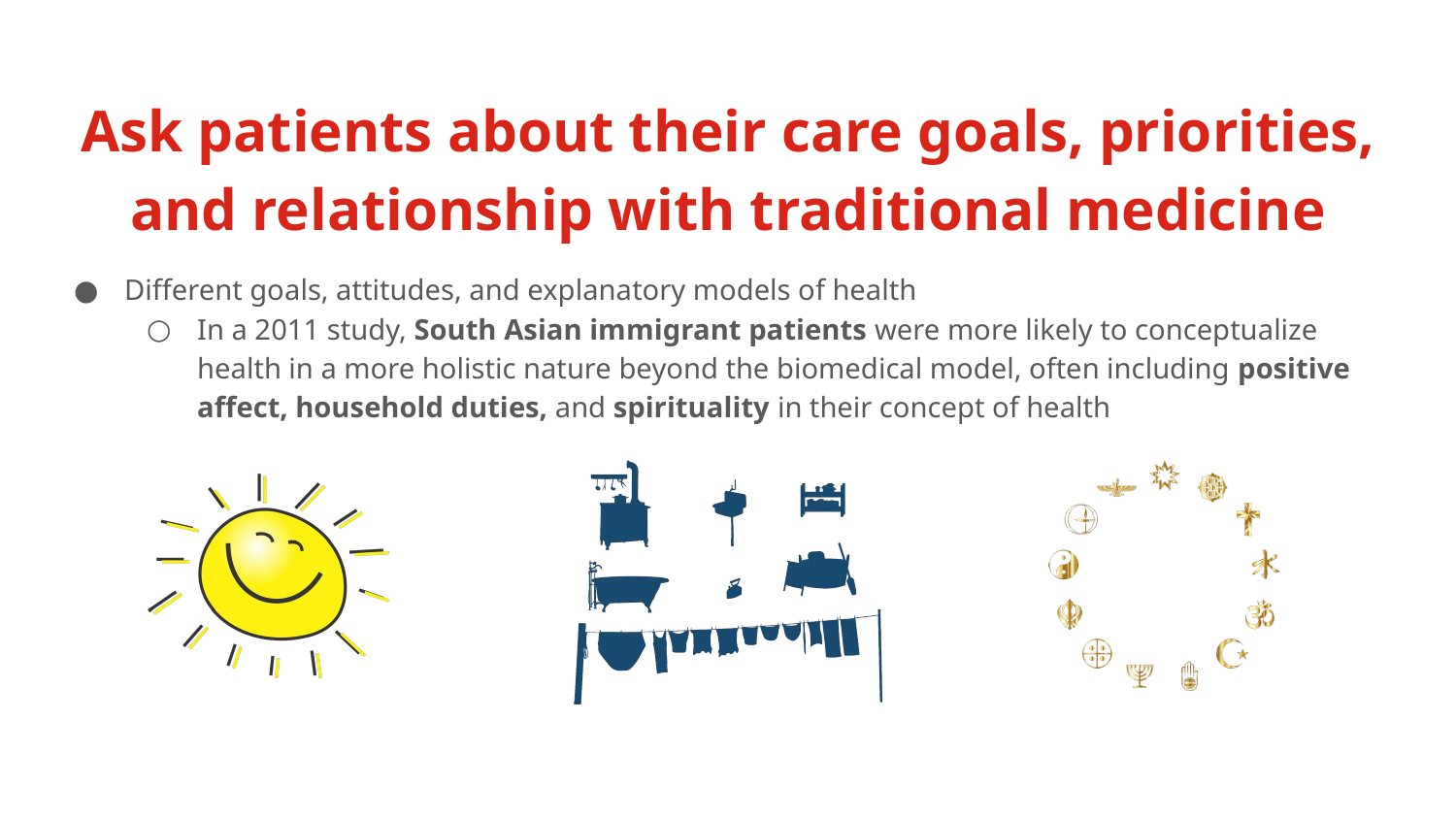

# Ask patients about their care goals, priorities, and relationship with traditional medicine
Different goals, attitudes, and explanatory models of health
In a 2011 study, South Asian immigrant patients were more likely to conceptualize health in a more holistic nature beyond the biomedical model, often including positive affect, household duties, and spirituality in their concept of health

## Slide 31
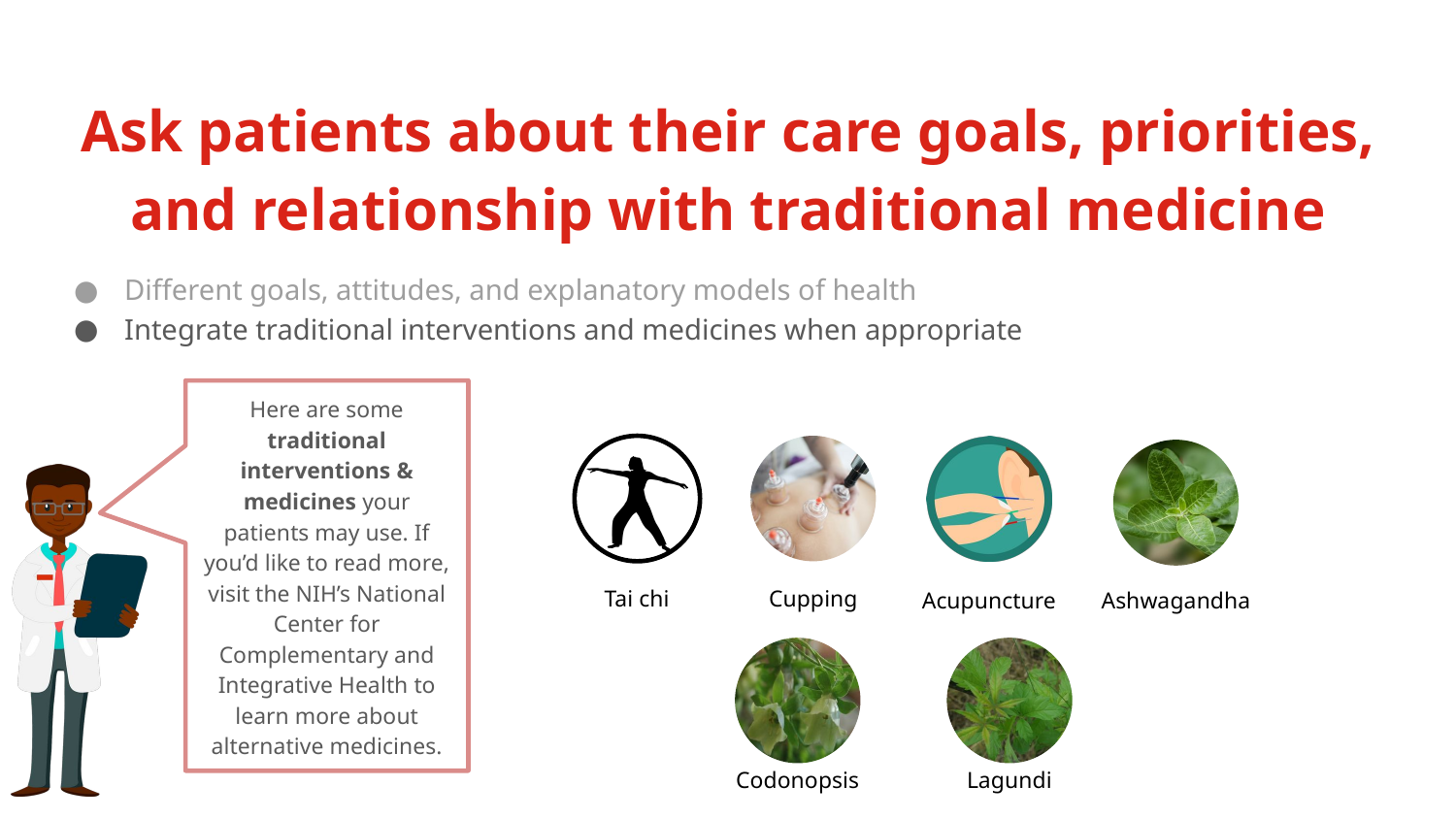

# Ask patients about their care goals, priorities, and relationship with traditional medicine
Different goals, attitudes, and explanatory models of health
Integrate traditional interventions and medicines when appropriate
Here are some traditional interventions & medicines your patients may use. If you’d like to read more, visit the NIH’s National Center for Complementary and Integrative Health to learn more about alternative medicines.
Tai chi
Cupping
Acupuncture
Ashwagandha
Codonopsis
Lagundi

## Slide 32
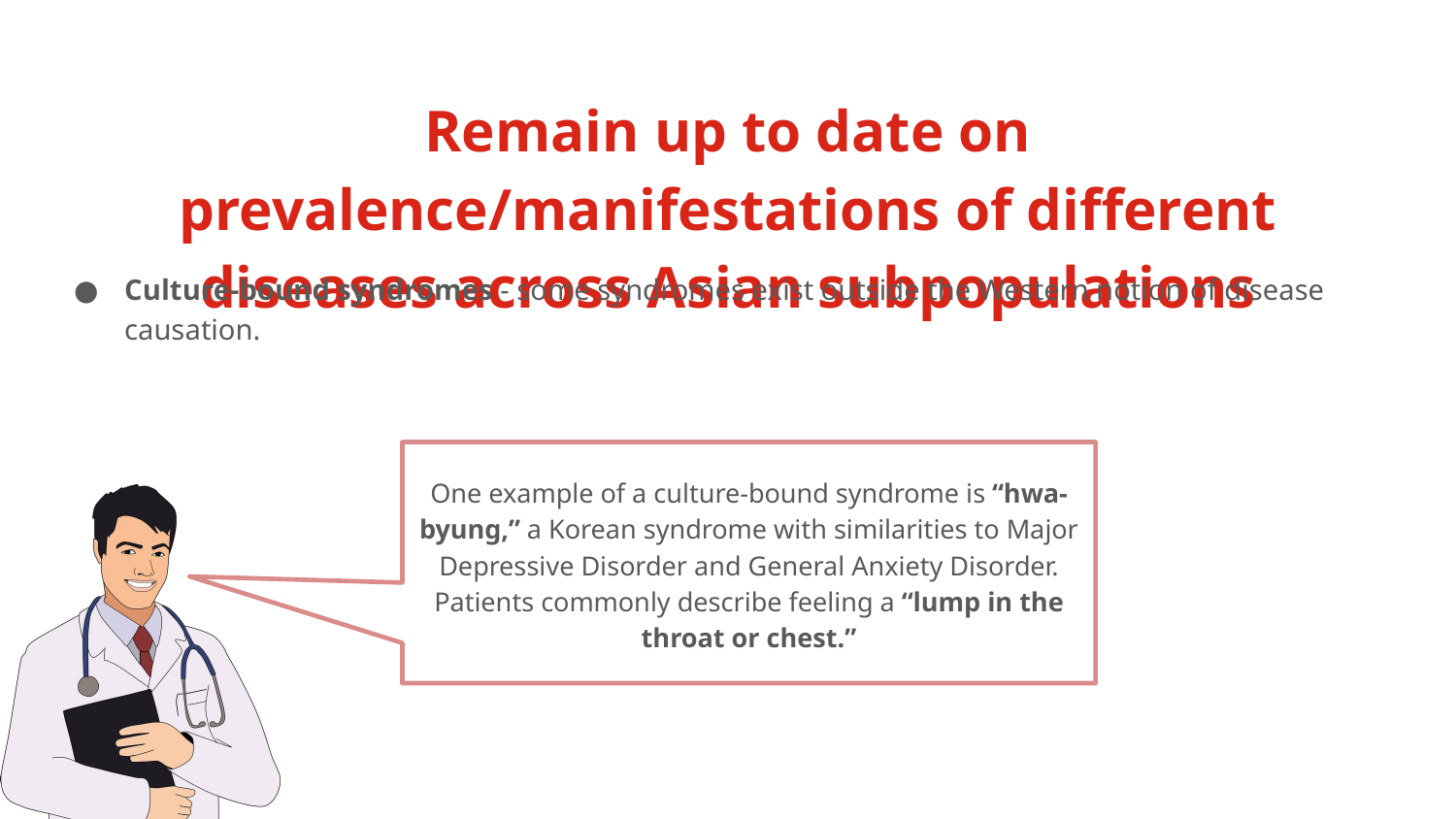

# Remain up to date on prevalence/manifestations of different diseases across Asian subpopulations
Culture-bound syndromes - some syndromes exist outside the Western notion of disease causation.
One example of a culture-bound syndrome is “hwa-byung,” a Korean syndrome with similarities to Major Depressive Disorder and General Anxiety Disorder. Patients commonly describe feeling a “lump in the throat or chest.”

## Slide 33
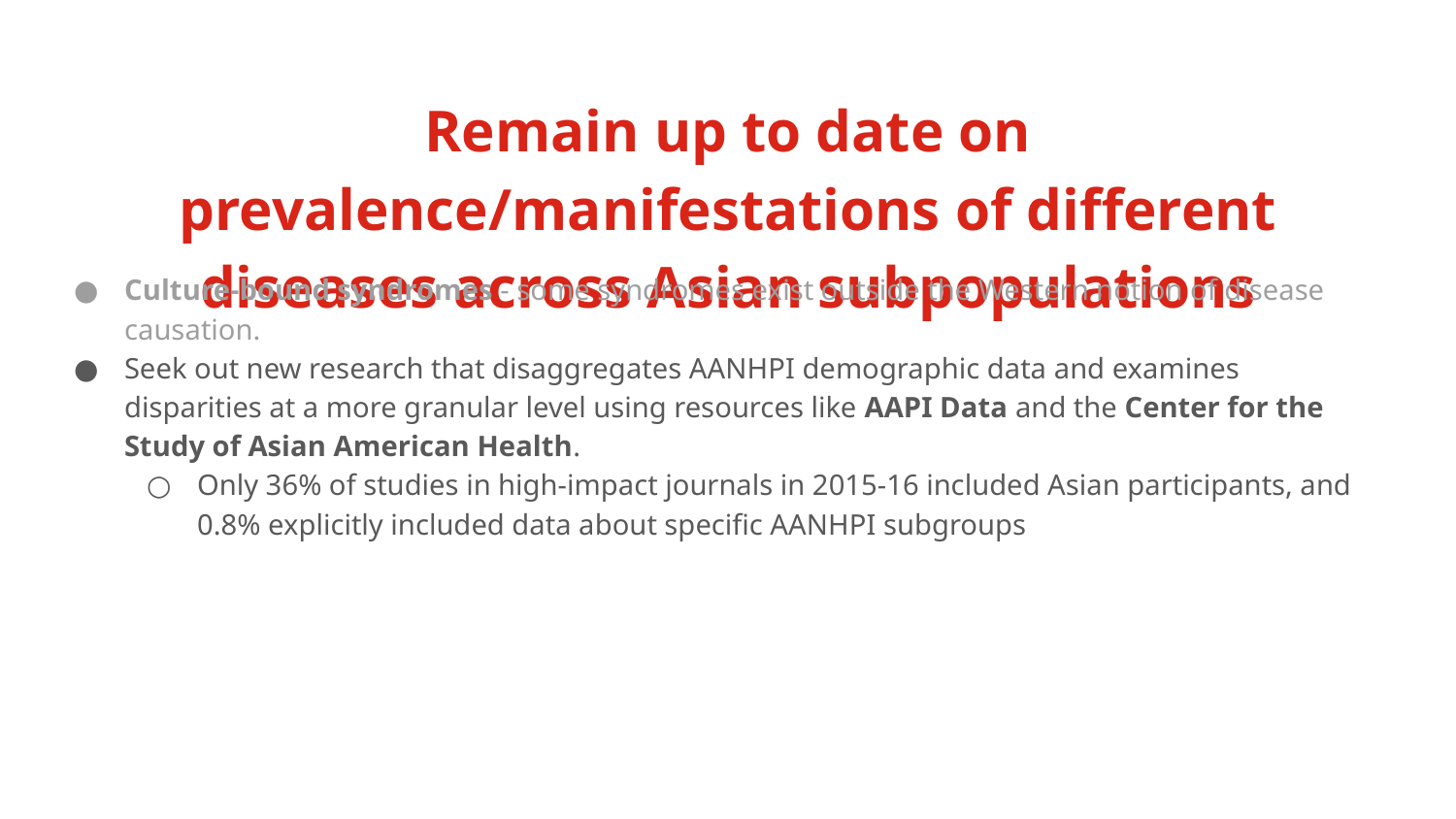

# Remain up to date on prevalence/manifestations of different diseases across Asian subpopulations
Culture-bound syndromes - some syndromes exist outside the Western notion of disease causation.
Seek out new research that disaggregates AANHPI demographic data and examines disparities at a more granular level using resources like AAPI Data and the Center for the Study of Asian American Health.
Only 36% of studies in high-impact journals in 2015-16 included Asian participants, and 0.8% explicitly included data about specific AANHPI subgroups

## Slide 34
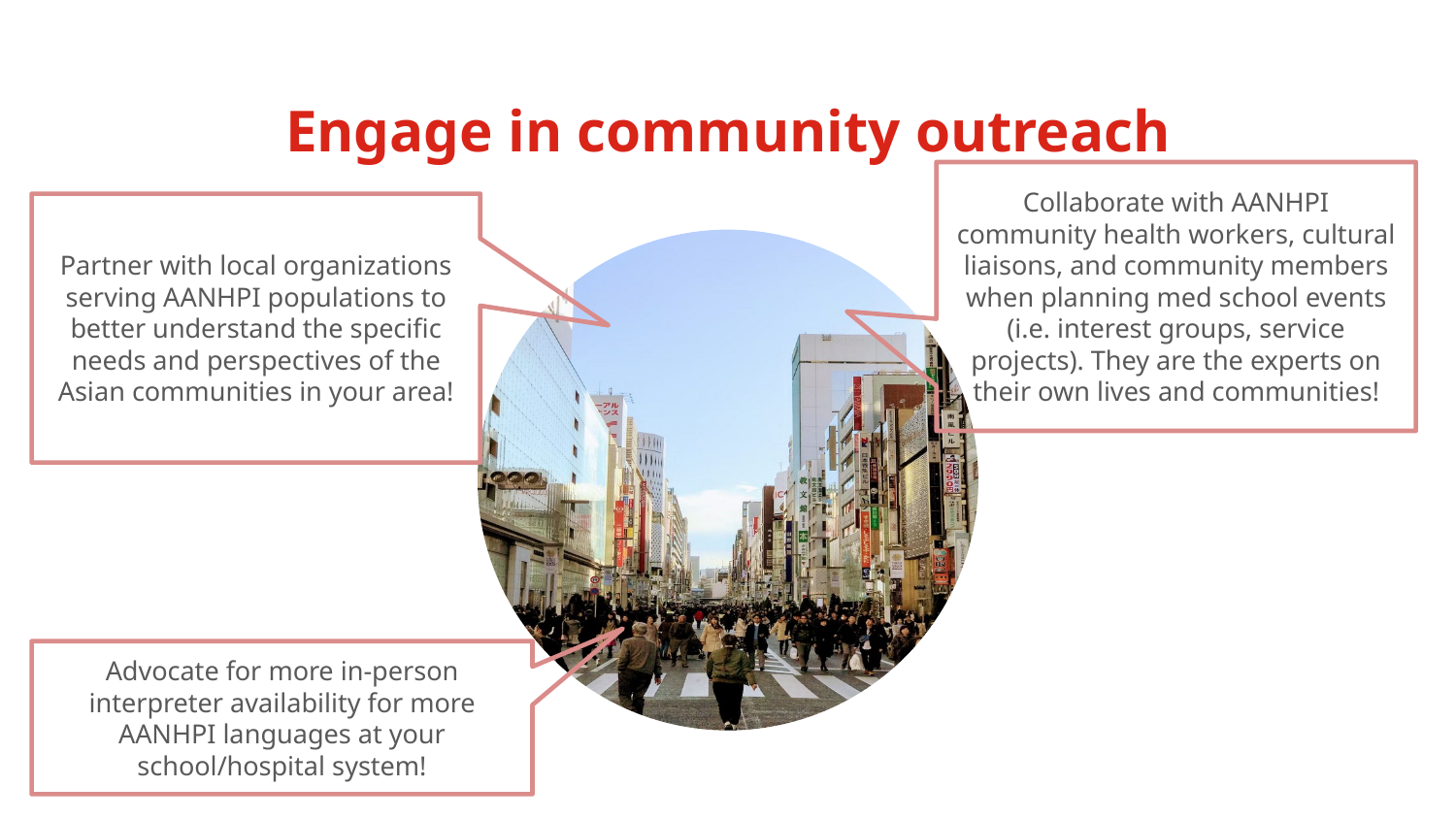

# Engage in community outreach
Collaborate with AANHPI community health workers, cultural liaisons, and community members when planning med school events (i.e. interest groups, service projects). They are the experts on their own lives and communities!
Partner with local organizations serving AANHPI populations to better understand the specific needs and perspectives of the Asian communities in your area!
Advocate for more in-person interpreter availability for more AANHPI languages at your school/hospital system!

## Slide 35
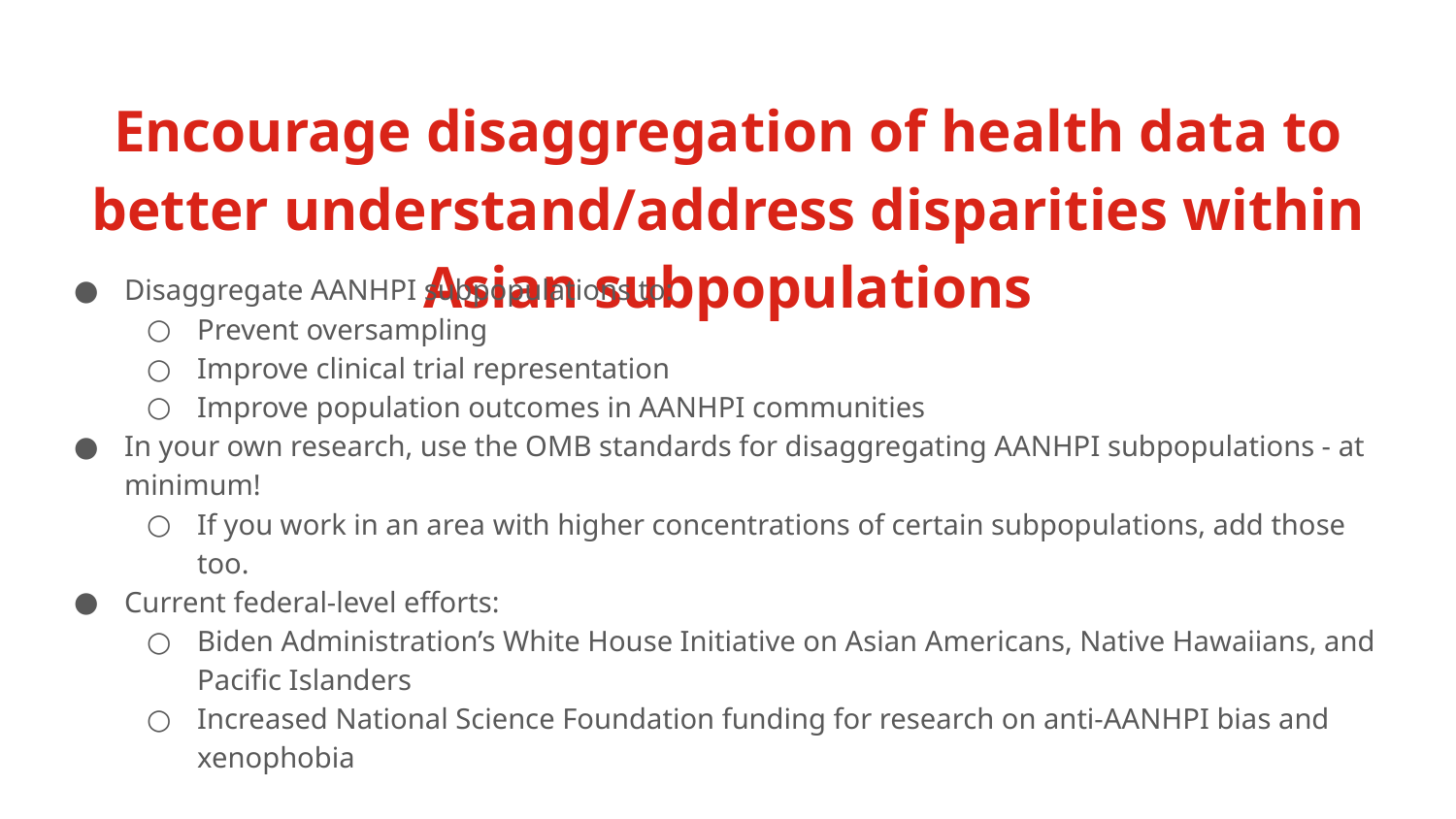

# Encourage disaggregation of health data to better understand/address disparities within Asian subpopulations
Disaggregate AANHPI subpopulations to:
Prevent oversampling
Improve clinical trial representation
Improve population outcomes in AANHPI communities
In your own research, use the OMB standards for disaggregating AANHPI subpopulations - at minimum!
If you work in an area with higher concentrations of certain subpopulations, add those too.
Current federal-level efforts:
Biden Administration’s White House Initiative on Asian Americans, Native Hawaiians, and Pacific Islanders
Increased National Science Foundation funding for research on anti-AANHPI bias and xenophobia

## Slide 36
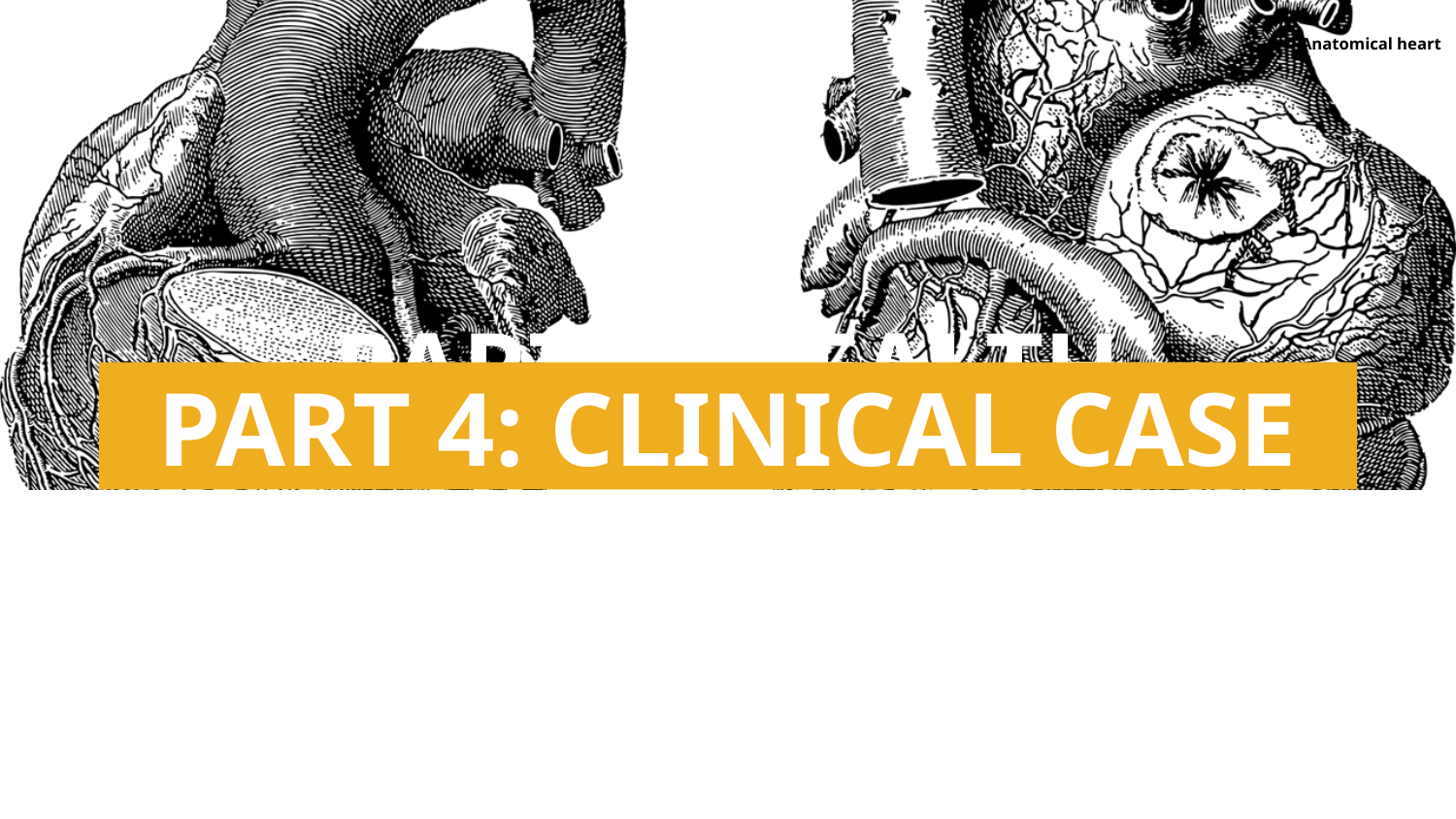

Anatomical heart
PART 3: HEALTH DISPARITIES
PART 4: CLINICAL CASE

## Slide 37
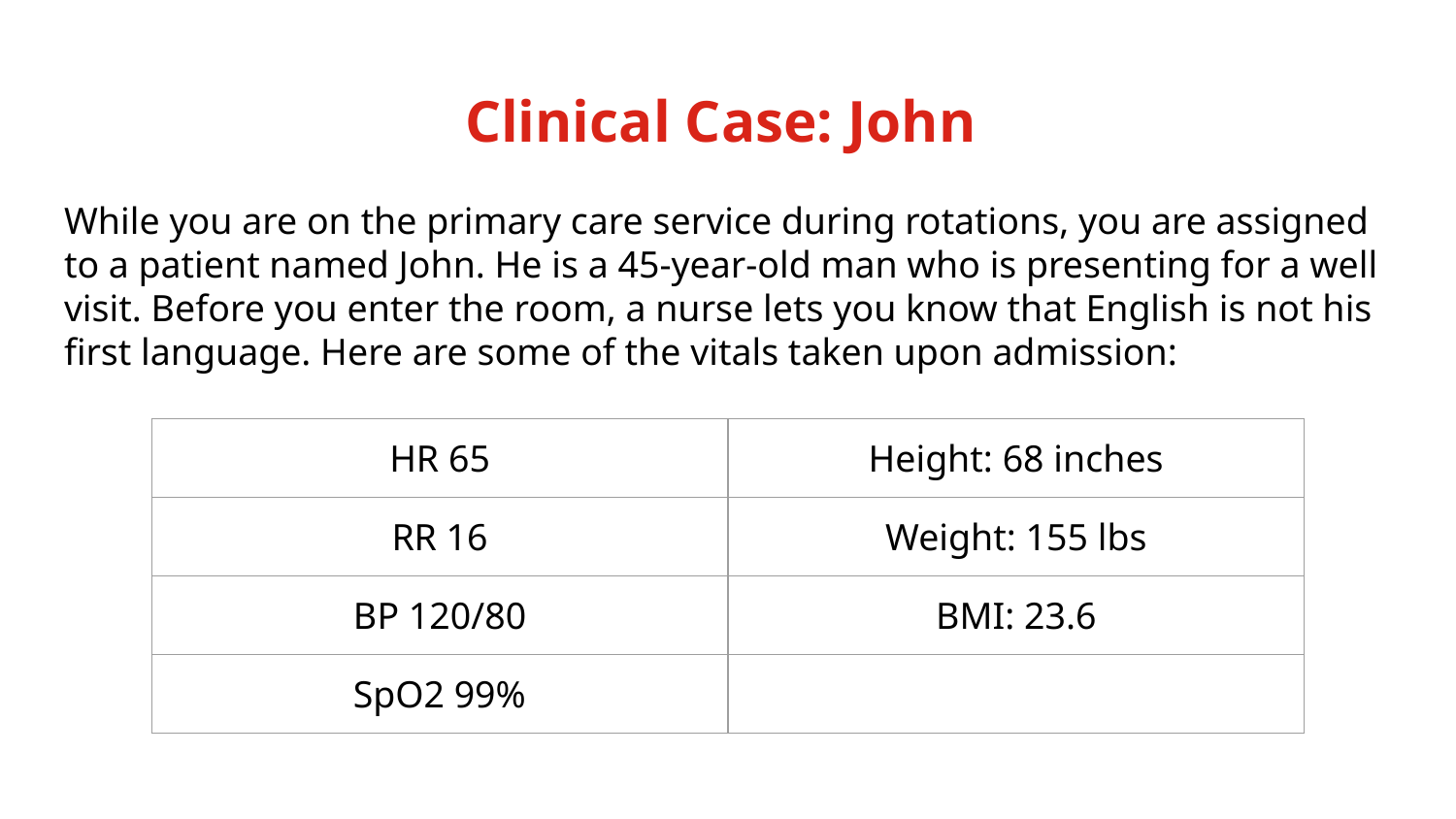

# Clinical Case: John
While you are on the primary care service during rotations, you are assigned to a patient named John. He is a 45-year-old man who is presenting for a well visit. Before you enter the room, a nurse lets you know that English is not his first language. Here are some of the vitals taken upon admission:
| HR 65 | Height: 68 inches |
| --- | --- |
| RR 16 | Weight: 155 lbs |
| BP 120/80 | BMI: 23.6 |
| SpO2 99% | |

## Slide 38
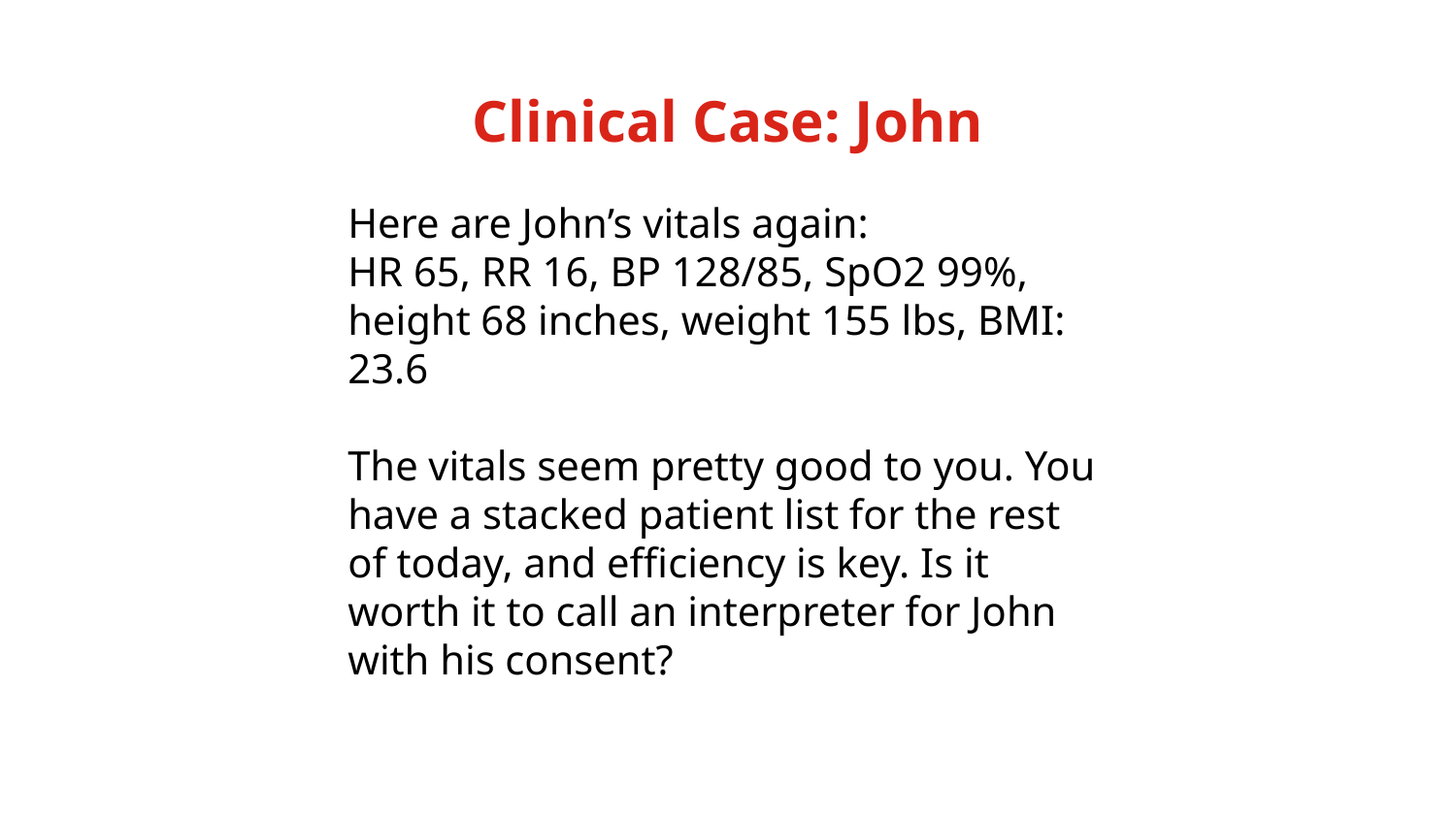

# Clinical Case: John
Here are John’s vitals again:
HR 65, RR 16, BP 128/85, SpO2 99%, height 68 inches, weight 155 lbs, BMI: 23.6
The vitals seem pretty good to you. You have a stacked patient list for the rest of today, and efficiency is key. Is it worth it to call an interpreter for John with his consent?

## Slide 39
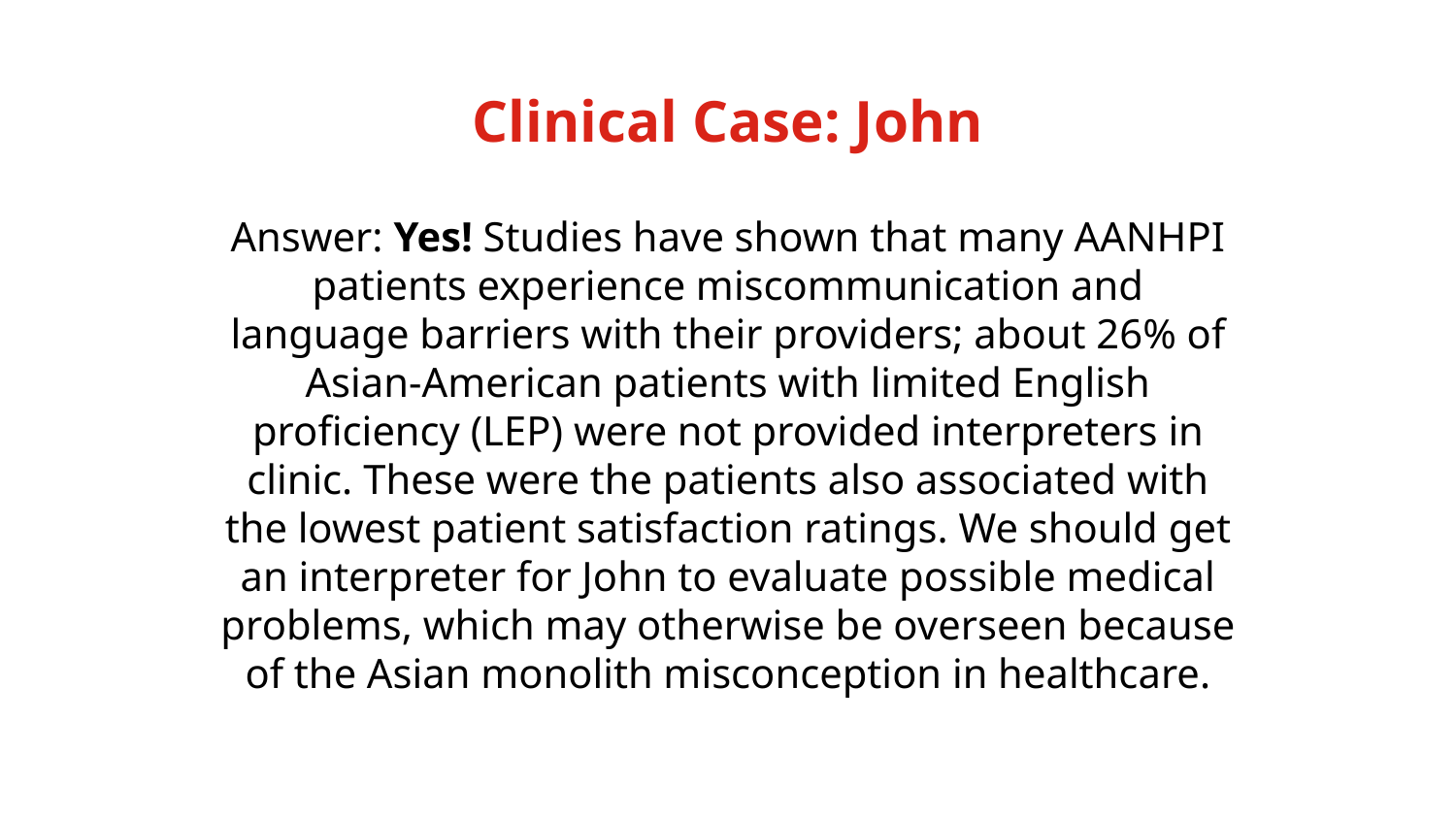

# Clinical Case: John
Answer: Yes! Studies have shown that many AANHPI patients experience miscommunication and language barriers with their providers; about 26% of Asian-American patients with limited English proficiency (LEP) were not provided interpreters in clinic. These were the patients also associated with the lowest patient satisfaction ratings. We should get an interpreter for John to evaluate possible medical problems, which may otherwise be overseen because of the Asian monolith misconception in healthcare.

## Slide 40
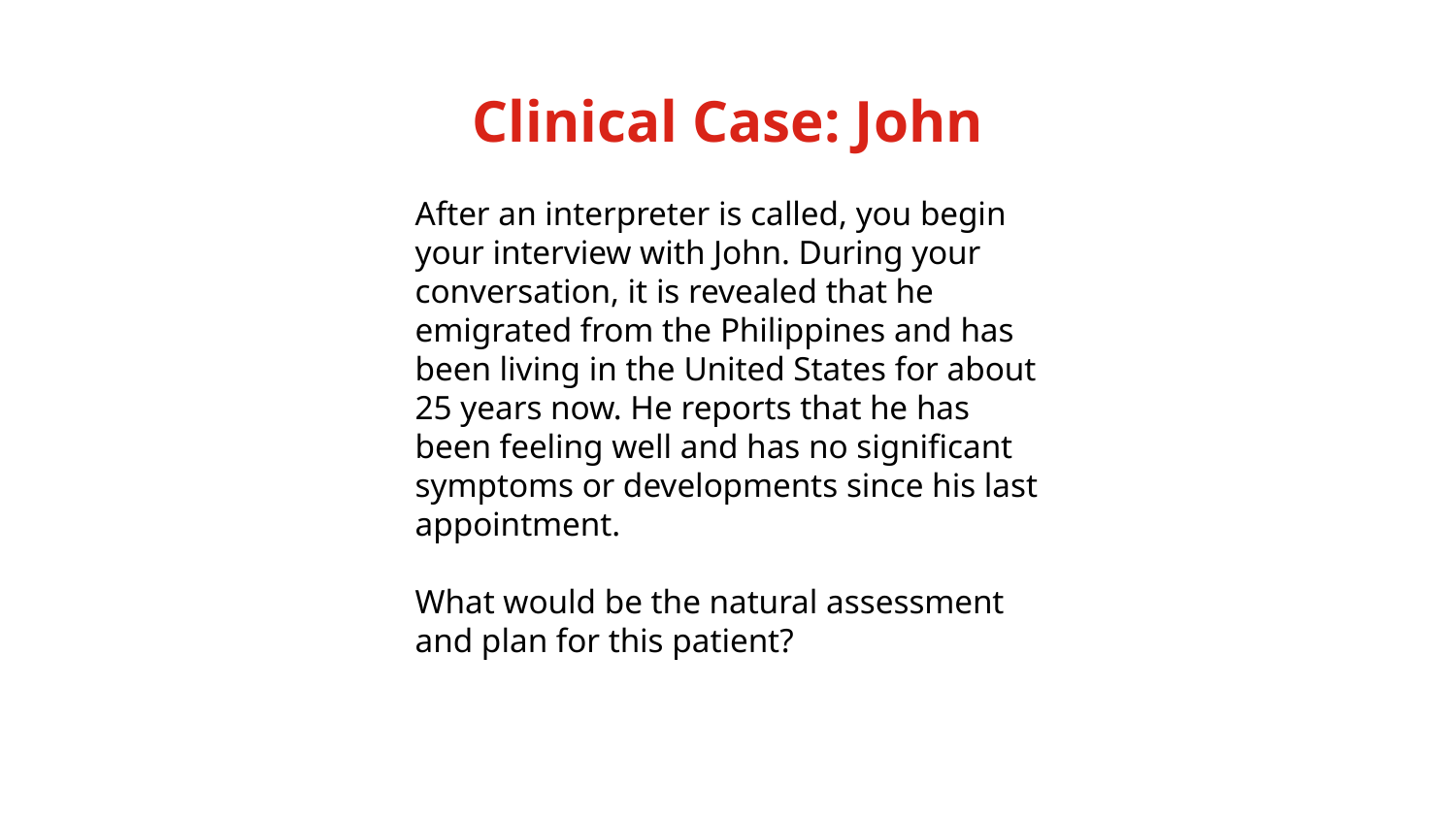

# Clinical Case: John
After an interpreter is called, you begin your interview with John. During your conversation, it is revealed that he emigrated from the Philippines and has been living in the United States for about 25 years now. He reports that he has been feeling well and has no significant symptoms or developments since his last appointment.
What would be the natural assessment and plan for this patient?

## Slide 41
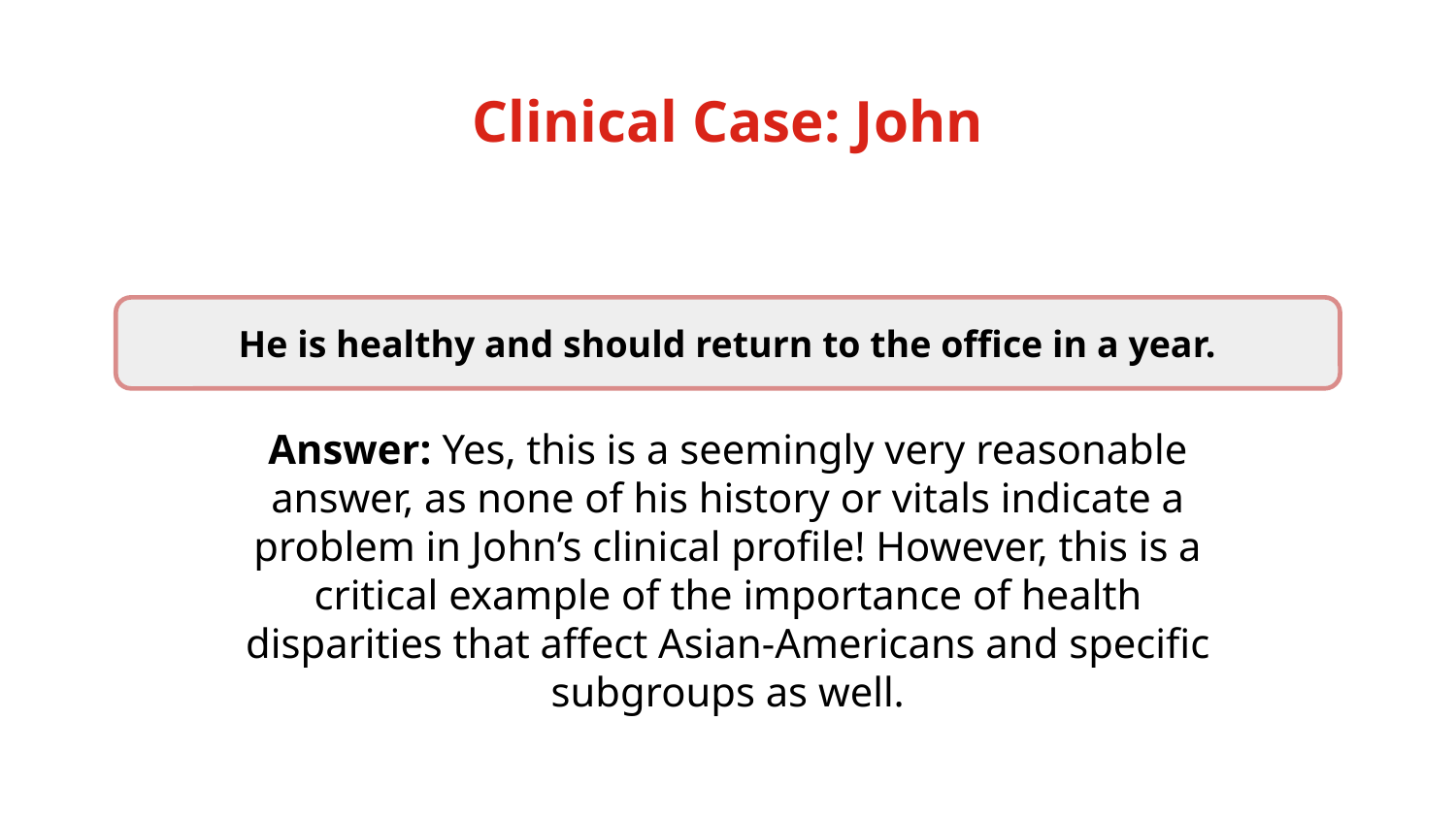

# Clinical Case: John
He is healthy and should return to the office in a year.
Answer: Yes, this is a seemingly very reasonable answer, as none of his history or vitals indicate a problem in John’s clinical profile! However, this is a critical example of the importance of health disparities that affect Asian-Americans and specific subgroups as well.

## Slide 42
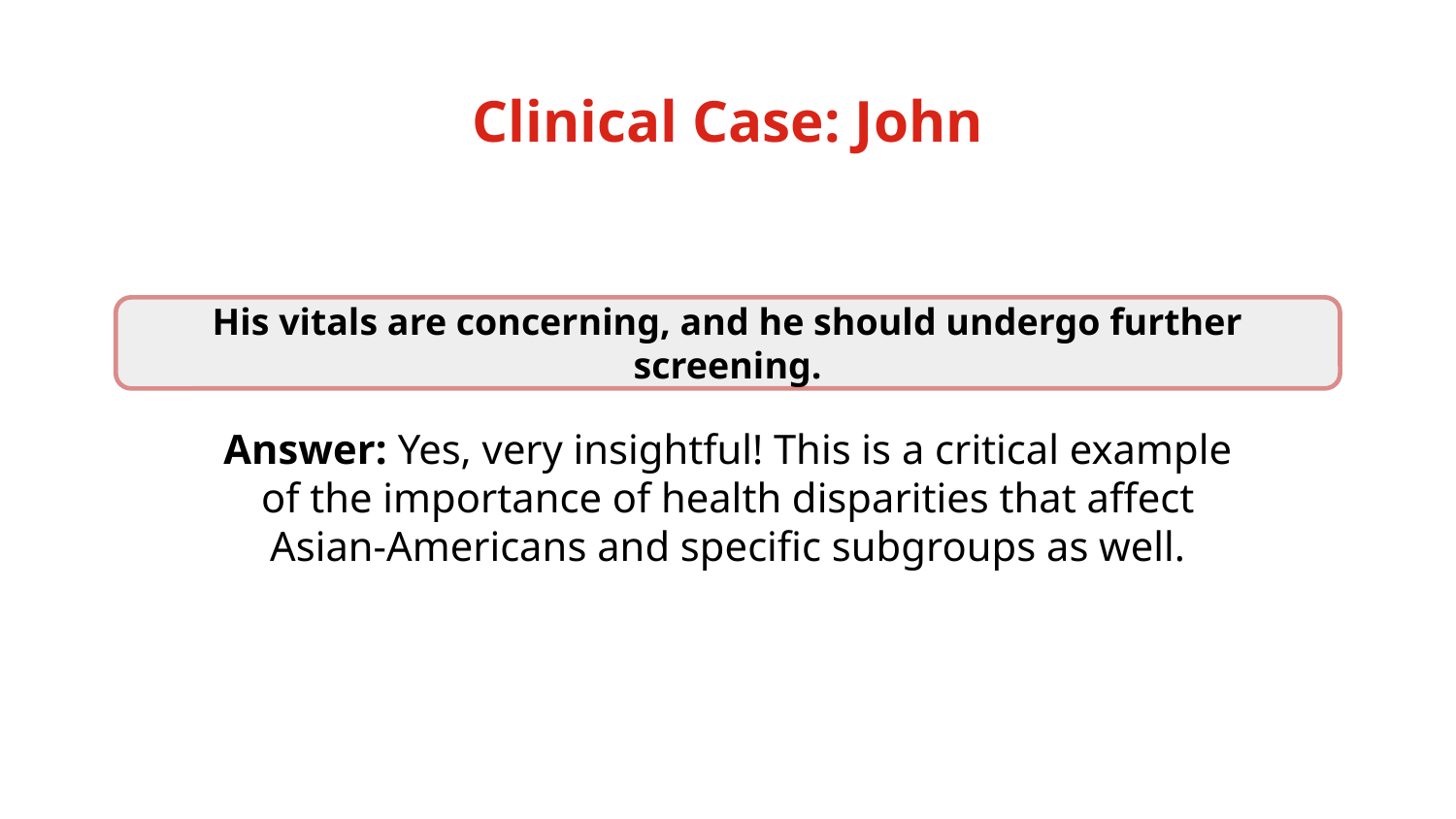

# Clinical Case: John
His vitals are concerning, and he should undergo further screening.
Answer: Yes, very insightful! This is a critical example of the importance of health disparities that affect Asian-Americans and specific subgroups as well.

## Slide 43
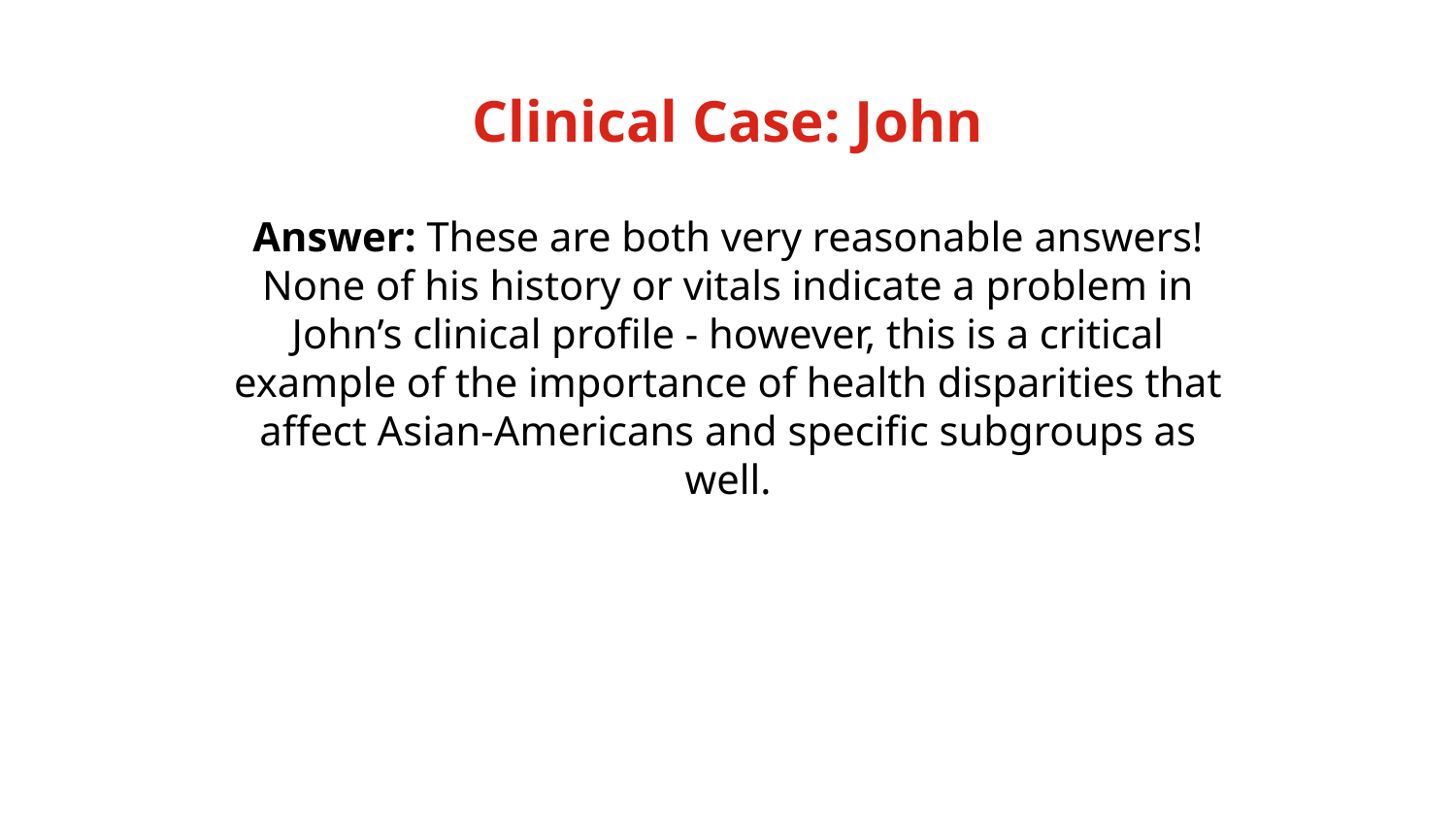

# Clinical Case: John
Answer: These are both very reasonable answers! None of his history or vitals indicate a problem in John’s clinical profile - however, this is a critical example of the importance of health disparities that affect Asian-Americans and specific subgroups as well.

## Slide 44
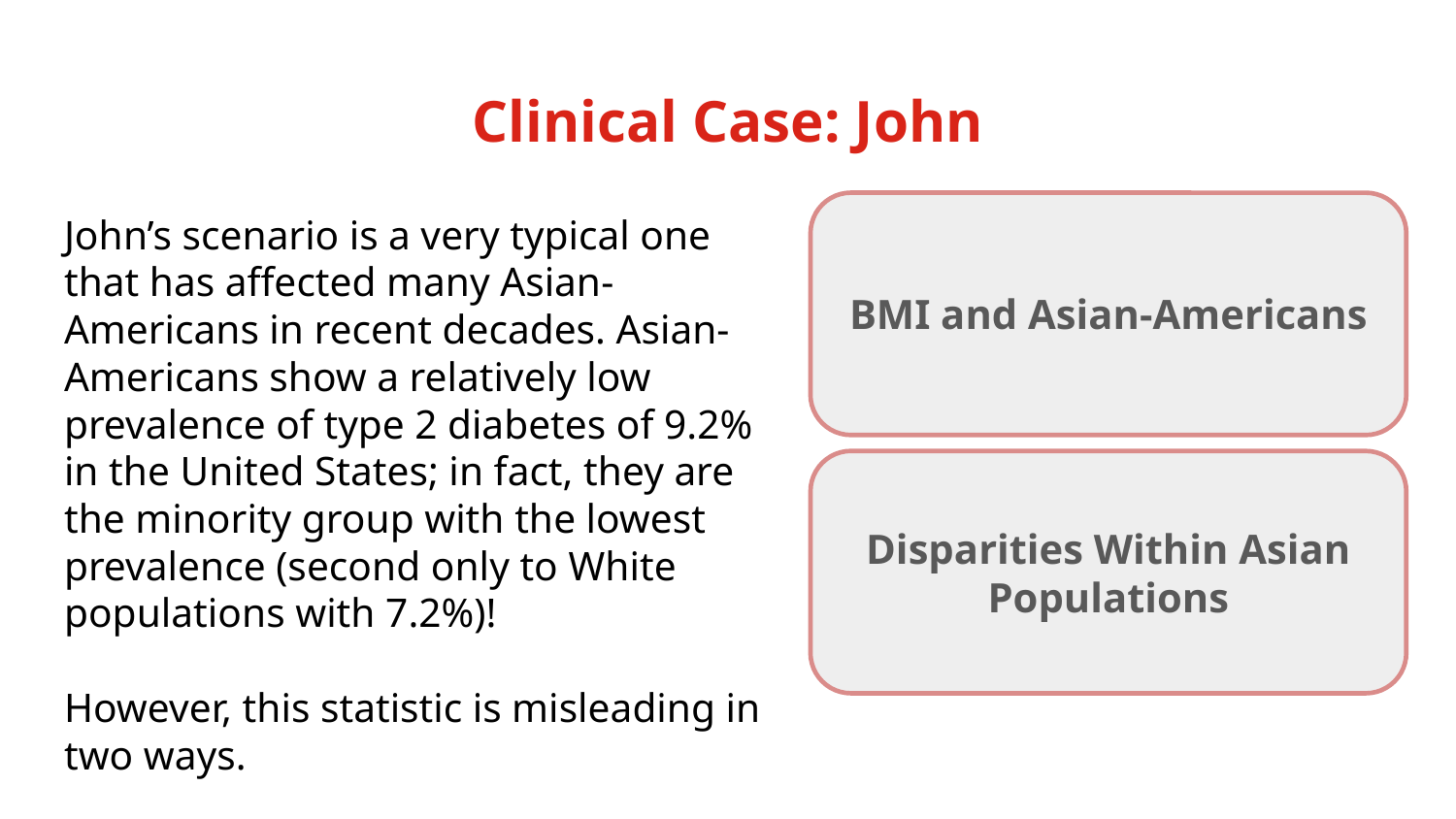

# Clinical Case: John
BMI and Asian-Americans
John’s scenario is a very typical one that has affected many Asian-Americans in recent decades. Asian-Americans show a relatively low prevalence of type 2 diabetes of 9.2% in the United States; in fact, they are the minority group with the lowest prevalence (second only to White populations with 7.2%)!
However, this statistic is misleading in two ways.
Disparities Within Asian Populations

## Slide 45
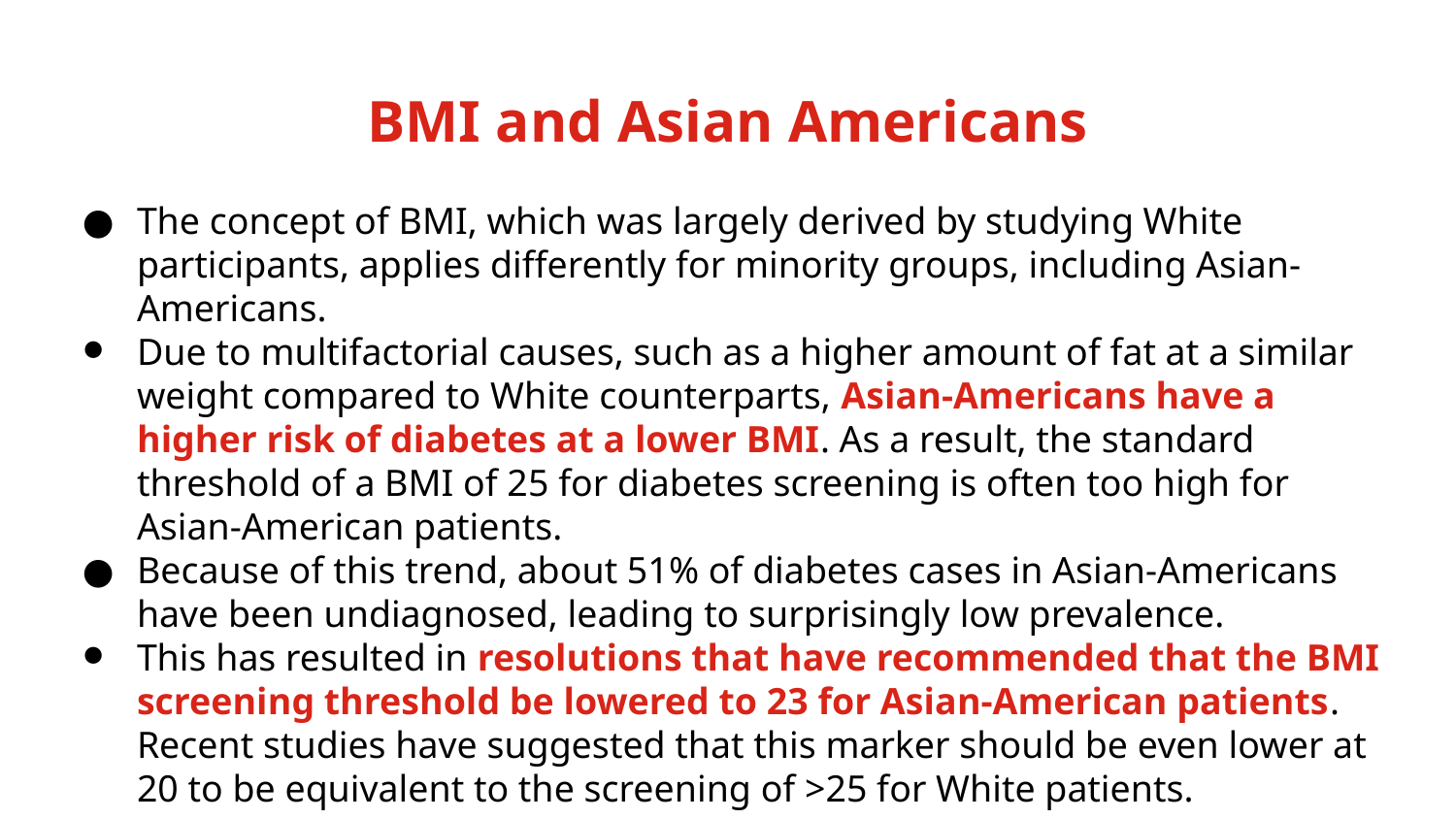

# BMI and Asian Americans
The concept of BMI, which was largely derived by studying White participants, applies differently for minority groups, including Asian-Americans.
Due to multifactorial causes, such as a higher amount of fat at a similar weight compared to White counterparts, Asian-Americans have a higher risk of diabetes at a lower BMI. As a result, the standard threshold of a BMI of 25 for diabetes screening is often too high for Asian-American patients.
Because of this trend, about 51% of diabetes cases in Asian-Americans have been undiagnosed, leading to surprisingly low prevalence.
This has resulted in resolutions that have recommended that the BMI screening threshold be lowered to 23 for Asian-American patients. Recent studies have suggested that this marker should be even lower at 20 to be equivalent to the screening of >25 for White patients.

## Slide 46
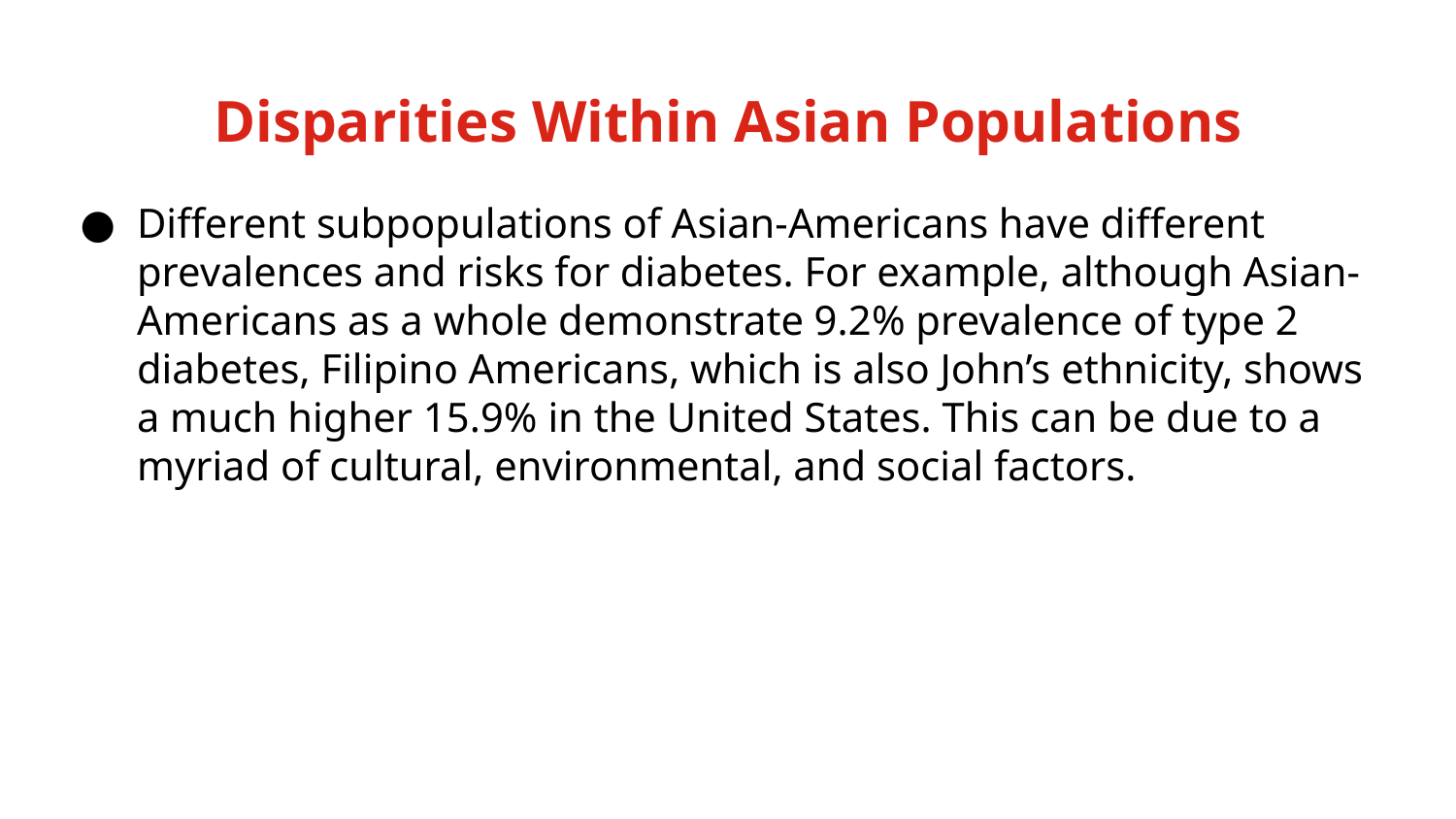

# Disparities Within Asian Populations
Different subpopulations of Asian-Americans have different prevalences and risks for diabetes. For example, although Asian-Americans as a whole demonstrate 9.2% prevalence of type 2 diabetes, Filipino Americans, which is also John’s ethnicity, shows a much higher 15.9% in the United States. This can be due to a myriad of cultural, environmental, and social factors.

## Slide 47
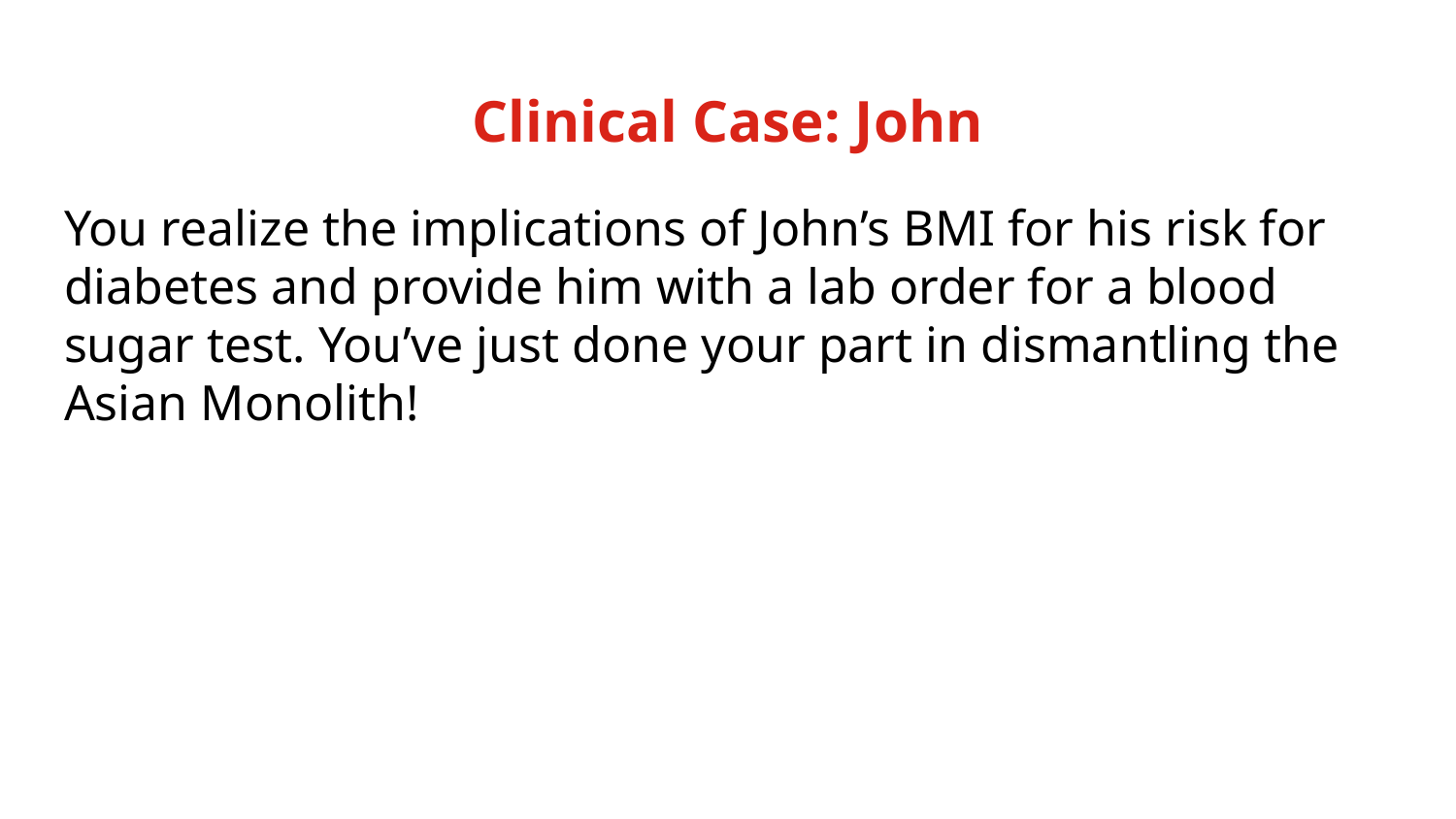

# Clinical Case: John
You realize the implications of John’s BMI for his risk for diabetes and provide him with a lab order for a blood sugar test. You’ve just done your part in dismantling the Asian Monolith!

## Slide 48
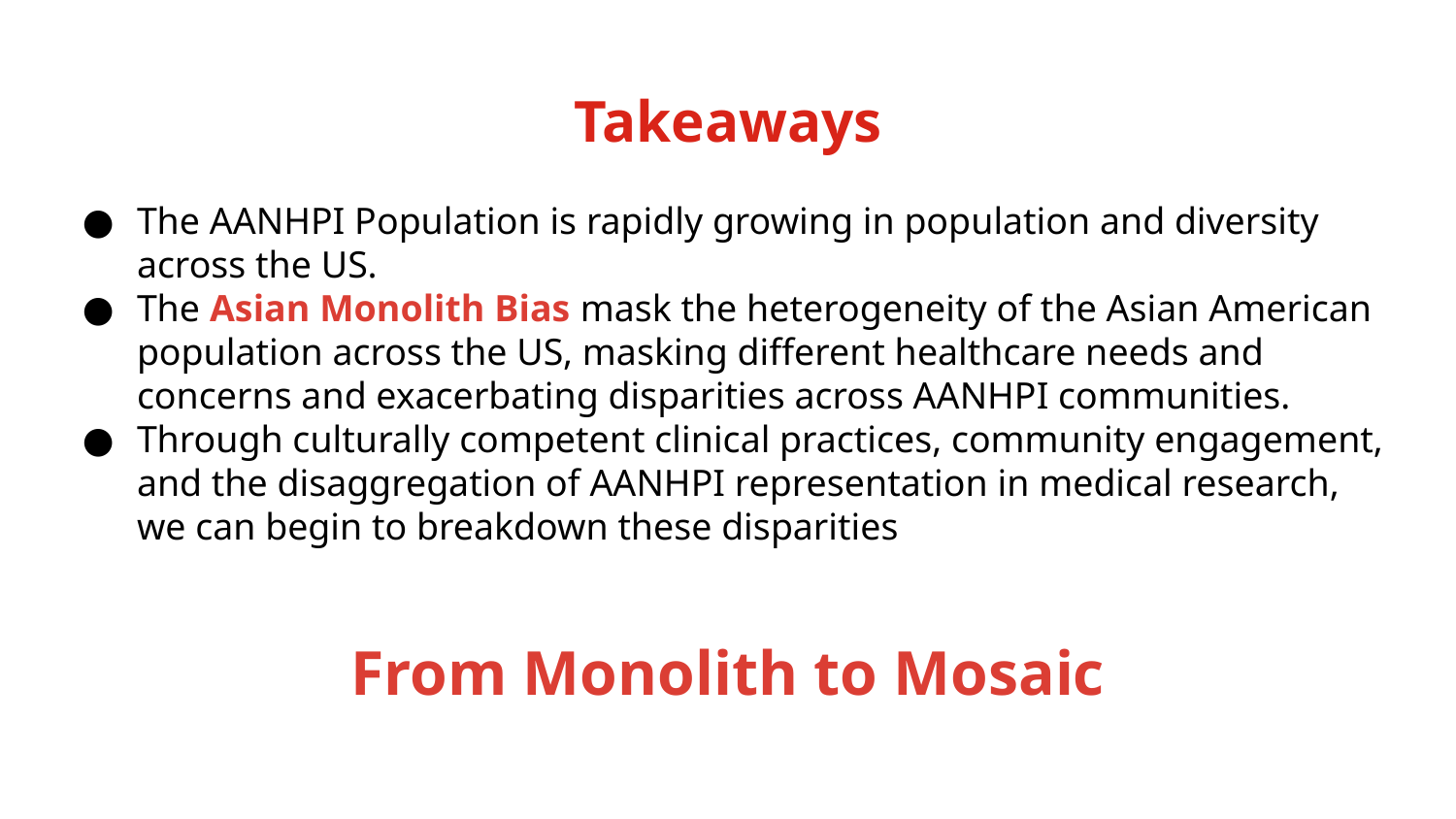

# Takeaways
The AANHPI Population is rapidly growing in population and diversity across the US.
The Asian Monolith Bias mask the heterogeneity of the Asian American population across the US, masking different healthcare needs and concerns and exacerbating disparities across AANHPI communities.
Through culturally competent clinical practices, community engagement, and the disaggregation of AANHPI representation in medical research, we can begin to breakdown these disparities
From Monolith to Mosaic

## Slide 49
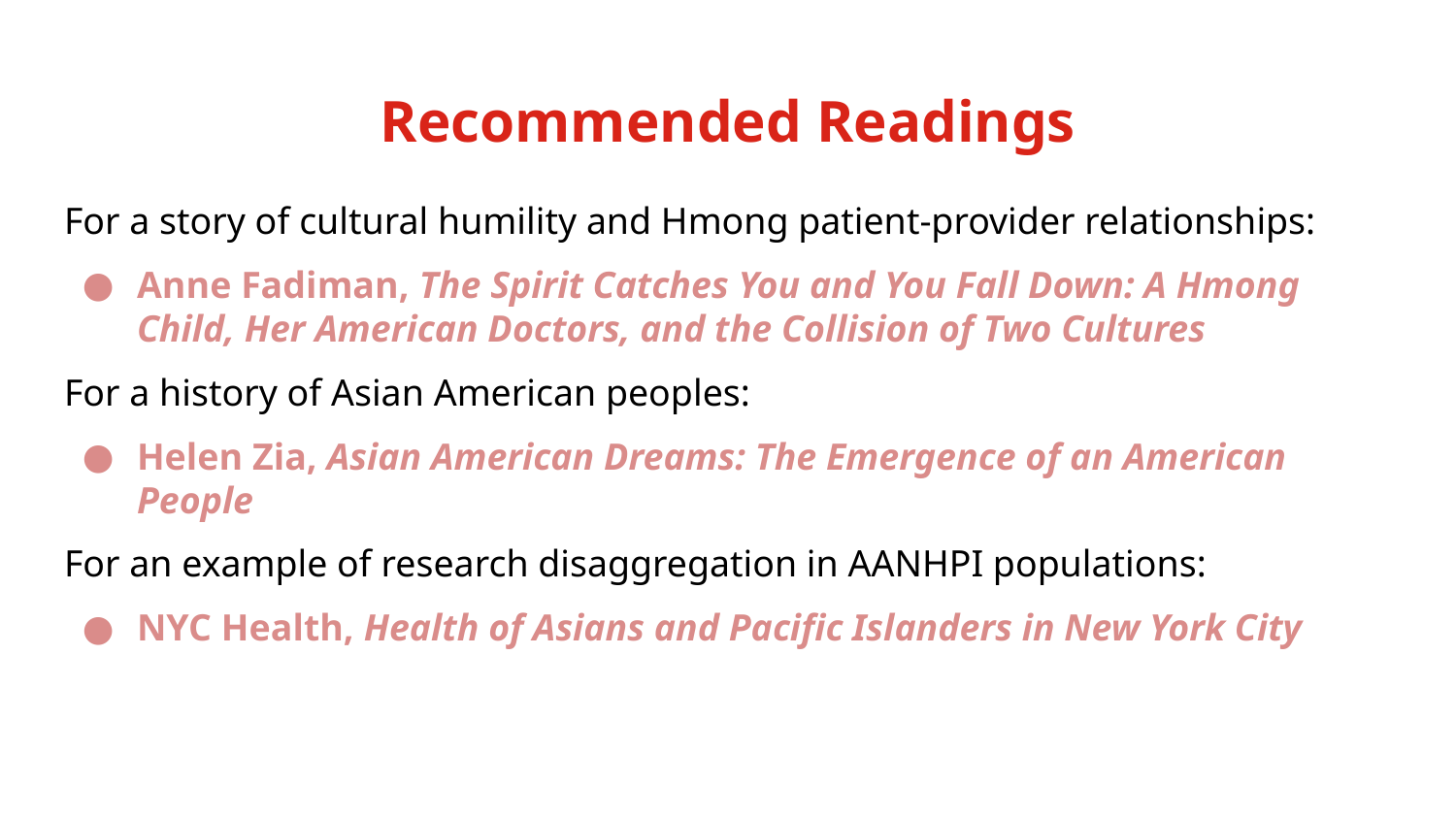

# Recommended Readings
For a story of cultural humility and Hmong patient-provider relationships:
Anne Fadiman, The Spirit Catches You and You Fall Down: A Hmong Child, Her American Doctors, and the Collision of Two Cultures
For a history of Asian American peoples:
Helen Zia, Asian American Dreams: The Emergence of an American People
For an example of research disaggregation in AANHPI populations:
NYC Health, Health of Asians and Pacific Islanders in New York City

## Slide 50
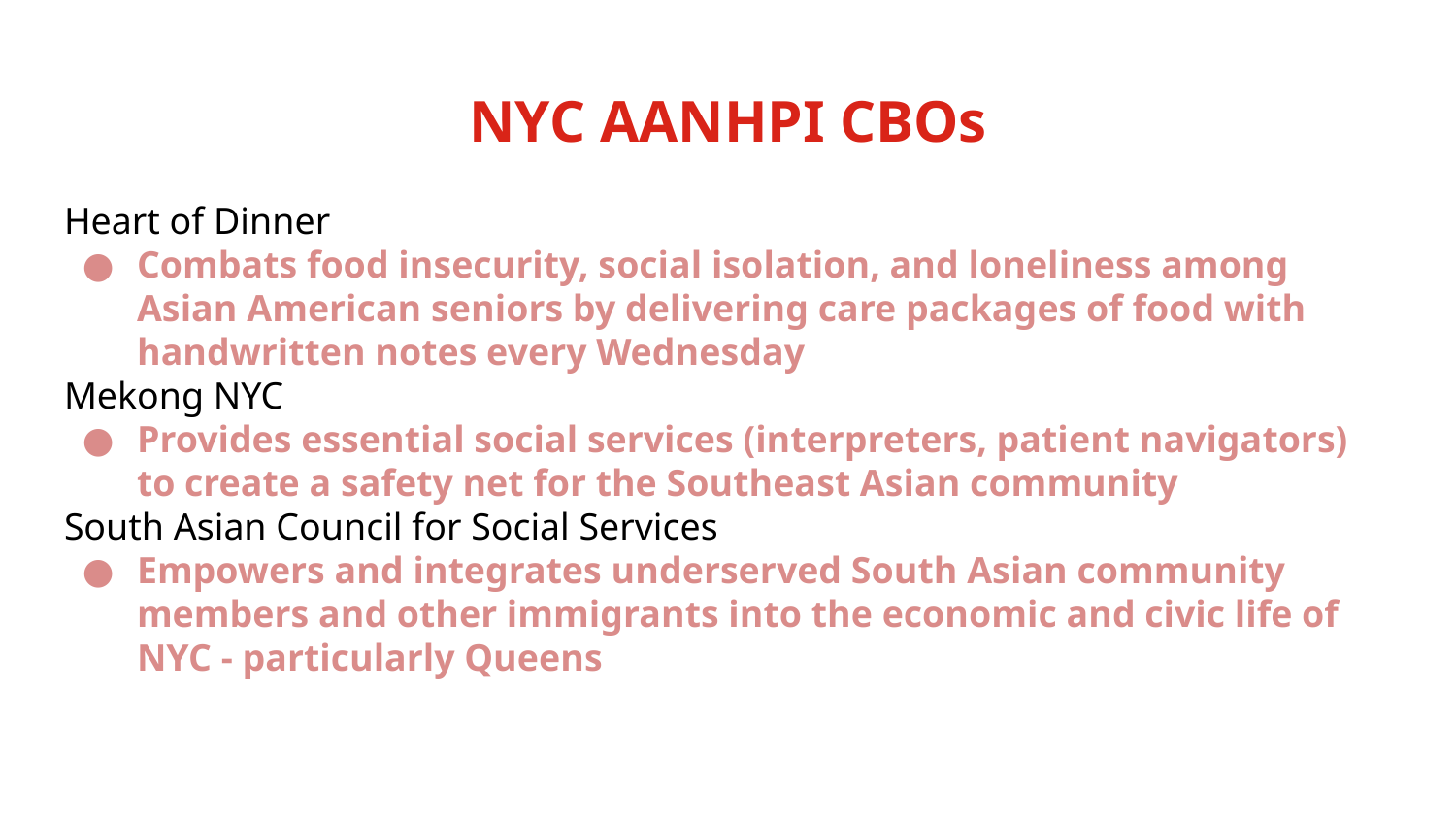

# NYC AANHPI CBOs
Heart of Dinner
Combats food insecurity, social isolation, and loneliness among Asian American seniors by delivering care packages of food with handwritten notes every Wednesday
Mekong NYC
Provides essential social services (interpreters, patient navigators) to create a safety net for the Southeast Asian community
South Asian Council for Social Services
Empowers and integrates underserved South Asian community members and other immigrants into the economic and civic life of NYC - particularly Queens
