# Supplementary material for: Deconstructing the Monolith: An Educational Module for Understanding Disparities Within Asian American, Native Hawaiian, and Pacific Islander Populations
Source: MedEdPORTAL. 2025 Jan 7;21:11480. doi: 10.15766/mep_2374-8265.11480 (PMC11697750; doi:10.15766/mep_2374-8265.11480)
Supplement: Supplementary file 1 — Monolith To Mosaic Presentation.pptxFacilitator Guide.docxPresurvey.docxPostsurvey.docx [file mep_2374-8265.11480-s001.zip › B. Facilitator Guide.docx]

**Deconstructing the Asian Monolith: An Educational Module for Improving Knowledge of the Asian Diaspora**

**Facilitator Guide**

**Overall Goals**

The goal of this module is to educate medical students, residents, and other health professionals on Asian American health inequities as well as strategies to tackle them. This module should be able to successfully engage learners from any background and promote a deeper understanding of the diverse healthcare needs across AANHPI subpopulations.

**Document Instructions**

The flow of the workshop is detailed in a 12-page document. This guide matches each PowerPoint slide (Appendix A) to key points facilitators should mention verbally and provides timing suggestions for optimal interactivity. It is adjusted for both the in-person and virtual versions of the workshop. It also provides prompts to the facilitator for when to pause sharing content for audience response to certain questions. We recommend taking 2 hours to review this guide alongside the PowerPoint presentation, and the Pre/Post-Survey Evaluations.

**Workshop Objectives**

1. Describe the historical context of the “Asian Monolith” stereotype in the United States.
2. Outline 3 ways in which the “Asian Monolith” stereotype negatively impacts health outcomes for diverse Asian communities.
3. Identify strategies to improve healthcare equity for Asian patients and communities.

**Workshop Handouts and Materials**

1. Computer setup with audio capability, and connection to projector
2. Printed pre-module evaluations (for students without internet access before presentation)
3. Printed post-module evaluations (for students without internet access after the presentation)

**Suggested Agenda and Timeline**

The length of this workshop is estimated to take slightly under 1 hour with the suggested timeline as follows.

- 5 min: Pre-workshop evaluation
- 5 min: Introduction (Slides 1-3)
- 10 min: The Asian Monolith Bias (slides 4-13)
- 10 min: Health Disparities (slides 14-21)
- 10 min: Tackling Disparities (slides 22-34)
- 10 min: Clinical Case (35-49)
- 10 min: Question and answer period/Post-workshop evaluation

**Slide Instructions**

*Speakers may wish to update the statistics listed in the presentation, depending on when/where this module is implemented.*

**Slide 1**:

*Facilitators should introduce themselves and explicitly note the incompleteness of the research required to deconstruct the Asian monolith. Facilitators should explain that the presentation will come in 4 parts: an introduction to the Asian Monolith Bias, a discussion of Health Disparities amongst Asian Subgroups, how to approach caring for an Asian patient, and a clinical case during which members of the audience can practice what they’ve learned.*

*We suggest facilitators acknowledge that they are following the content and direction of this peer-reviewed module, and further resources will be shared for outstanding questions that may not be answered here. At this point, facilitators should set the stage for the interchangeable terminology that will be used throughout the presentation.*

*Optionally, facilitators can embed Poll Everywhere (PollEV) questions directly into their PowerPoint using the following instructor guide:* [*https://www.polleverywhere.com/poll-everywhere-instructor-guide.pdf*](https://www.polleverywhere.com/poll-everywhere-instructor-guide.pdf)*. Otherwise, facilitators can simply ask for crowd interaction live, via raising hands or calling out answers.*

**Slide 2:**

*Facilitators should read aloud the learning objectives and remind the audience that a post-module evaluation will be used to quantify the effectiveness of this learning module in addressing the 3 objectives.*

**Slide 3:**

Before we begin, we must define what it means when we state AANHPI. AANHPI stands for Asian-American, Native Hawaiian, and Pacific Islander.

AANHPI participants in the Census identified across 31 self-described groups

Asian Americans are among the fastest-growing ethnic groups in the United States. In the 2020 US Census, 20 million participants identified as AANHPI (6.2% of the US population). This number is projected to reach 36.8 million by 2060.

**Slide 4:**

“Part 1: The Asian Monolith Bias” provides important background information regarding the AANHPI community.

*Before proceeding, facilitators can ask if anyone has heard of the “Asian monolith bias” and, if there are any volunteers, what their understanding is of the term.*

**Slide 5:**

The Asian Monolith Bias homogenizes all AANHPI individuals. It stems from a tendency to view all Asians as a **monolithic entity**, failing to recognize the diversity within the Asian American communities.

This bias often manifests in stereotypes, treating individuals as representatives of their entire ethnic group. It dismisses the unique perspectives, struggles, and achievements of different Asian ethnic groups.

The Asian Monolith Bias causes healthcare disparities because it ignores the health needs that exist among different Asian ethnic groups.

**Slide 6:**

*Facilitators will walk the audience through the timeline as an overview of the legal oppression of Asians, then the following slides will emphasize specific events.*

Origins of the Asian Monolith Bias began in 1870 when we saw the first inclusion of a category for people of Asian Descent in the US Census.

**Slides 7: Interactive**

*Facilitators should now present this slide, asking participants to provide their best guesses.*

*Optionally, PollEV can be embedded into the slide here and used for this question.*

**Slide 8:**

It wasn’t until 1870 that Asians were first acknowledged in the US Census. But inclusion wasn’t comprehensive by any means. This classification was initially labeled as "Chinese." All individuals of Asian Descent fell into this category. Over time, we eventually saw the gradual inclusion of more inclusive ethnic categories for several Asian sub-populations. It was not until 100 years later, in 1970, we saw the mention of Vietnamese on the US Census.

**Slide 9:**

As more Asian Americans began to migrate to the US, driven by labor demands, Anti-Asian sentiments and sinophobia spread across the US, fueled by economic competition, xenophobia, and racial prejudice.

Discriminatory legal actions, including the 1882 Chinese Exclusion Act and the 1924 Immigration Act, were enacted to limit Asian immigration and rights. Around WWII, over 100,000 Japanese Americans were placed in Japanese Internment Camps.

During this period, U.S. military activities, such as the overthrow of the Hawaiian Monarchy, also led to environmental degradation and socioeconomic disparities among Native Hawaiian and Pacific Islander populations.

**Slide 10:**

The Immigration and Nationality Act of 1965 resulted in a rapid rise in Asian immigration to the US.

There was an increase in the migration of graduate students, scientists, engineers, and artists, but primarily from India, China, Japan, and Korea.

Other ethnic subgroups, such as those from Vietnam and Cambodia, typically arrived in the U.S. as refugees due to instability and safety concerns in their respective homelands.

The circumstances under which these groups migrated to America continue to

contribute to disparities today, yet all Asians are thought of as having similar histories.

**Slide 11:**

The Asian American Diaspora continued to grow within the US in the 1970s and the Civil Rights Act granted numerous Asian Americans improved opportunities in terms of housing, education, and employment. Yet, disparities across Asian American communities continue to grow.

**Slide 12:**

Then, In 1985, The Secretary’s Task Force published a report on Black and Minority Health, also known as the Heckler Report.

This stated that Asian American populations were “healthier” than all other racial groups in the United States. In doing so, it significantly masked disparities that continued to impact specific AANHPI subgroups.

**Slide 13:**

Most recently, the COVID-19 Pandemic highlighted the stark healthcare disparities across AANHPI populations.

Studies demonstrated that Asian Americans had a higher likelihood of hospitalization in comparison to non-Hispanic whites during the pandemic.

More importantly, The impact of the pandemic varied considerably among AANHPI individuals based on factors such as immigration status, length of time residing in the country, and primary language spoken.

Across the US, the Chinese, Vietnamese, Filipino, and Japanese populations exhibited significantly higher rates of hospitalization compared to the Asian Indian, and Korean subpopulations.

There were notable disparities also observed in testing protocols, healthcare outcomes, and treatment across various AANHPI subgroups.

**Slide 14:**

This run-through helps us understand the rapid rise in the AANHPI population and diversity across the United States over the past century.

Despite this, existing research has largely considered Asian Americans as one monolithic group that has been faring better than the general population, rather than as separate and unique groups with their own healthcare needs.

This Asian Monolith bias results in a history of propagating discrepancies across AANHPI populations.

Let’s now discuss a few of the many ways healthcare needs are different across several AANHPI populations.

**Slide 15:**

Part 2: Health Disparities. This section will address the several discrepancies in healthcare across AANHPI subpopulations

**Slide 16:**

There are many ways in which healthcare needs differ across AANHPI Populations:

Workforce Representation

Prevalence of Disease

Screening Rates

Social Factors

Health Behaviors

**Slide 17:**

Identifying those who are underrepresented in medicine allows us to develop a more diverse physician workforce that reflects our patient population. Patients often have unique healthcare needs that can be better understood by healthcare providers who share their cultural backgrounds.

Due to the Asian monolith bias, medical school admissions data tend to group Asian American subgroups, concealing the disparities in representation that exist among Southeast Asians, specifically Laotians, Cambodians, and Indonesians. Disaggregating the data and recognizing these disparities can help medical schools develop targeted strategies to recruit and support individuals from underrepresented Southeast Asian communities. By increasing the diversity of our physician workforce, we bring justice and equity to medical education and provide more personalized and culturally informed care to our patients.

**Slide 18:**

The Asian monolith bias can influence how providers form differential diagnoses and impede the development of subgroup-specific interventions. By generalizing the diseases that affect Asian Americans, providers may fail to consider certain conditions that have higher prevalence among specific Asian subgroups.

Gastric Cancer: Although Asian Americans as a whole have significantly higher incidence rates of gastric adenocarcinoma than non-Hispanic whites, Korean Americans have a 13.3-fold higher incidence risk, in contrast to Filipino Americans who have a 1.81-fold higher incidence risk.

Colon Cancer: Hmong and Pacific Islander patients have an increased risk of presenting with a more advanced stage of colon cancer when compared to White patients and even when compared within AANHPI subgroups to Chinese patients.

Perinatal Complications: Perinatal complications like preeclampsia, gestational diabetes, and preterm delivery are more common in Filipina women compared to other Asian subgroups. Similarly, Pacific Islanders face higher obesity rates, leading to increased pregnancy-related comorbidities.

Cardiovascular Disease: Asian Americans aggregated as a group are at a lower risk of atherosclerotic cardiovascular disease (ASCVD) than non-Hispanic Whites but South Asians and Native Hawaiians were found to have higher proportional mortality rates from ASCVD than non-Hispanic Whites, as well as other Asian American subgroups.

**Slide 19: Interactive**

*Facilitators should now present this slide, asking participants to provide their best guesses.*

*Optionally, PollEV can be embedded into the slide here and used for this question.*

**Slide 20:**

Though screening rates are low across all groups, rates of specific screenings also differ among subgroups. Korean Americans have the lowest rates of various types of screenings among Asian subgroups. Disaggregating data allows us to identify barriers that are specific to this population and eliminate them through targeted interventions that aim to increase rates of screening. For instance, a study showed that Korean American women were more likely to obtain cancer information from the Internet and Korean ethnic media. These avenues could therefore be used for health promotion and cancer prevention targeting Korean American women.

**Slide 21:**

Asian Americans are often known as the “model minority” and are stereotyped as educated, wealthy, and successful. This myth masks the diversity of Asian subgroups in their household income, education status, English proficiency, and insurance status, all of which are important social determinants of health.

Studies have shown that physicians tend to overestimate the knowledge Asian American patients have regarding their health, resulting in inadequate care.

Making connections:

Screening rates are the lowest among Korean Americans. This may be due to the high uninsured rates found in this population and interventions to improve access to health insurance may increase screening rates.

**Slide 22:**

Certain health behaviors are especially common within Asian ethnic groups and recognizing these is crucial to delivering culturally-sensitive care. These critical differences are often ignored through the biases that stem from the Asian monolith bias.

Filipinos had the highest current smoking rates among Asian ethnic groups, doubling the smoking rates of Asian Indian and Chinese Americans

Chinese-style salted fish is popular among Southern Chinese and Southeastern Asian populations and studies have demonstrated its consumption is associated with increased risk of nasopharyngeal cancer and stomach cancer. Providers may omit dietary counseling with Asian American patients due to failure to account for differences in diets.

Traditional healers like dhami-jakhri play a huge role in health care for resettled Bhutanese refugees. Encouragement may be needed for Bhutanese patients to feel more comfortable sharing their traditional treatments and practices with their providers.

**Slide 23:**

Part 3: Tackling Disparities.

**Slide 24:**

So how can you improve patient-provider relationships amongst your future Asian American patients?

First, do not make assumptions about patients’ cultural backgrounds based on their appearance, name, or speech

Take a thorough social history. Though this certainly applies to all your future patients, it’s especially important when your patient may have a cultural background that is completely unfamiliar and different from your own.

Also, you should ask patients about their care goals, priorities, and if relevant, relationship with traditional medicine. Do not assume that a patient does or does not ascribe to Western medicine.

Remain up to date on the prevalence/manifestations of different diseases across Asian subpopulations

The bottom line is: until you ask, you do not know

Now we’re going to dive into each one of these tips in more detail over the next few slides

**Slide 25:**

Do not make assumptions about patients’ cultural backgrounds based on their appearance, name, or speech

It can be natural to ascribe a last name to a certain ethnic group based on previous interactions. However, the history and background behind names is often complex.

For example, The last name “Lee” can be romanized in many ways (Li, Rhee, Yee, Yi, Lý). It is commonly a Chinese, Korean, or Vietnamese name. However, some patients may have Chinese, Korean, or Vietnamese heritage but have settled down elsewhere for many generations

**Slide 25:**

Never hesitate to ask about a patient’s preferred language.

For example, there are more than 100 different dialect subgroups spoken in China, including multiple indigenous languages and languages that are not mutually intelligible. Even if a patient speaks some Mandarin, the language they are most comfortable communicating in may be another language or dialect. You may not know unless you ask!

**Slide 27: Interactive**

Next, let's talk about how to take a thorough social history with your future Asian American patients. Can anyone shout out topics that would be important to ask about?

*Facilitators should provide time for participants to provide answers, using prompts when needed such as: “What are some questions you might ask in all your patients?”*

*Optionally, PollEV can be embedded into the slide here and used for this question.*

**Slide 28:**

Those are great ideas. Here are some important topics that could be important to ask in addition to medical history, depending on your specific patient:

Education & Employment - understanding your patient’s health literacy and work environment is very important.

Family & Community - some patients may want family to be more involved in forming a care plan.

Religion & Spirituality - for example, patients who observe Ramadan may need adjustments to their medication schedules.

Intergenerational & Cultural Trauma - examples include Japanese American internment, the India/Pakistan partition, and the Cambodian genocide. These events may still have effects on our patients’ physical and psychiatric health today.

Migration Experiences - new migrants may struggle with acculturation, employment, seeking asylum status, and loneliness or isolation.

Experiences with Racism - for instance, a past experience of racism in a nearby park may make your patient feel unsafe to return to that area, limiting their options for exercise.

**Slide 29: Interactive**

*Facilitators should now present this slide, asking participants to provide their best guesses.*

*Optionally, PollEV can be embedded into the slide here and used for this question.*

**Slide 30:**

The next tip is to ask patients about their care goals, priorities, and if relevant, relationship with traditional medicine.

Each patient will have different goals, attitudes, and explanatory models of health

For instance, In a 2011 study, South Asian immigrant patients often included positive affect, household duties, and spirituality in their concept of health. This can and should affect how you discuss and collaborate in making a care plan for each patient.

**Slide 31:**

If a patient does wish to pursue traditional interventions and medicines, consider integrating them into their care as much as is safe.

Examples of common traditional practices or medicines you may encounter include tai chi, cupping, acupuncture, and supplements such as ashwagandha, codonopsis, and lagundi. Ask your patients about any supplements or home remedies they use and what they use them for. Look up known interactions of any of these supplements with other medications, and consider whether it is possible for the patient to continue using them safely. Even if not, having open conversations with patients and bringing a humble attitude to learning about non-Western medicines can go a long way in building trust and rapport.

**Slide 32:**

The next tip to keep in mind when treating Asian American patients: Remain up to date on the prevalence and manifestations of different diseases across Asian American subpopulations.

Earlier, we talked about different diseases having different prevalence across Asian American communities. In addition to that, there are entities known as “culture-bound syndromes” which are syndromes that are relatively common within certain cultures but not officially acknowledged in Western medicine.

One example is “hwa-byung,” a Korean syndrome with similarities to Major Depressive Disorder and General Anxiety Disorder. Patients commonly describe feeling a “lump in the throat or chest.” In many cultures, psychiatric symptoms may be more likely to present as somatic symptoms.

**Slide 33:**

Luckily, new research is emerging that disaggregates AANHPI demographic data. Resources such as AAPI Data and the Center for the Study of Asian American Health provide up-to-date research that breaks down the Asian American monolith.

This is extremely important work - in 2015-16, only 36% of studies in high-impact journals included Asian participants, and 0.8% explicitly included data about specific AANHPI subgroups

**Slide 34:**

As future doctors, it is also important to be aware of and ideally to work alongside community centers for Asian patients to improve healthcare on a systems level.

We encourage you to consider this in your event planning and advocacy undertakings to better understand the specific needs and perspectives of the Asian communities in your area. For example, advocate for better in-person interpreter availability for a wider range of languages within your hospital system.

**Slide 35:**

Finally, doctors can play an active role in disaggregation of health data to better understand/address disparities within Asian American subpopulations

Disaggregating AANHPI subpopulations will eventually:

Prevent oversampling

Improve clinical trial representation

Improve population outcomes in AANHPI communities

In your own research, use the OMB standards for disaggregating AANHPI subpopulations - at minimum!

If you work in an area with higher concentrations of certain subpopulations, add those too.

Current federal-level efforts:

Biden Administration’s White House Initiative on Asian Americans, Native Hawaiians, and Pacific Islanders

Increased National Science Foundation funding for research on anti-AANHPI bias and xenophobia

With that, we’ll move on to applying some of these tips to a clinical encounter.

**Slide 36:**

Now we’ll discuss a clinical case where you all get to act as the physician to an Asian patient. We’ll ask you several closed-ended questions to keep the story moving, but there will also be opportunities to share ideas more generally. So don’t be afraid to speak your mind!

**Slide 37:**

While you are on the primary care service during rotations, you are assigned to a patient named John dela Cruz. He is a 45-year-old man who is presenting for a well-visit. Before you enter the room, a nurse lets you know that English is not his first language. Here are some of the vitals taken upon admission:

Heart Rate 65 bpm

Respiratory rate: 16

Height: 68 inches

Weight: 155 lbs

Blood Pressure: 120/80

Body Mass IndexI: 23.6

Oxygen level: 99%

**Slide 38:**

With a reminder of the vitals at the top, we can see that John’s condition is not life-threatening at this point. So should we call an interpreter with his consent?

*Facilitators should provide time for participants to provide answers.*

*Optionally, PollEV can be embedded into the slide here and used for this question.*

**Slide 39:**

Yes!

Studies have found that about 26% of Asian-American patients with limited English proficiency (LEP) were not provided interpreters in clinics. These were the patients also associated with the lowest patient satisfaction ratings.

We should get an interpreter for John to evaluate possible medical problems, which may otherwise be overseen if a physician fell for the Asian monolith misconception. For instance, a different physician might think that because John is Asian (and has an anglicized first name) he has better health and English proficiency than in reality.

**Slide 40:**

After an interpreter is called, you begin your interview with John. During your conversation, it is revealed that he has been living in the United States for about 25 years now. He reports that he has been feeling well and has no significant symptoms or developments since his last appointment.

What would be the natural assessment and plan for this patient?

*Facilitators should now present this slide, asking participants to provide their best guesses.*

*Optionally, PollEV can be embedded into the slide here and used for this question.*

**Slide 41/42:**

To be fair, it is reasonable to send John home just based on his vitals. However, if we did send him home, we might have missed a key health disparity that affects Asian Americans.

**Slide 43:**

In fact, a different physician may have looked at John and his BMI and concluded that everything was normal, especially since Asians have the lowest prevalence of type 2 diabetes.

**Slide 44:**

Supposedly, Asian Americans show a relatively low prevalence of type 2 diabetes at 9.2% in the United States; in fact, they are the minority group with the lowest prevalence.

However, this statistic is misleading in two ways.

**Slide 45:**

However, what is BMI? It was first derived from studying White patients - so not as generalizable as most might think.

It turns out that Asian Americans actually have a higher risk of diabetes at a lower BMI. So whereas the standard BMI for diabetes screening is 25, this cut-off is too high, leaving 51% of diabetes cases in Asian-Americans undiagnosed.

Therefore, resolutions have recommended that the BMI screening threshold be lowered to 23 for Asian-American patients. Some studies show it should be 20 to correlate with the screening of 25+ for White patients.

**Slide 46:**

There are diabetes disparities amongst different Asian subgroups as well. Filipino-Americans (John’s nationality) have an average diabetes rate of 15.9%!

**Slide 47:**

Luckily, you realized that John’s BMI puts him at risk and ordered him a blood sugar test. Well done, you’ve just done your part in dismantling the Asian Monolith!

**Slide 48:**

We hope you can take away a few lessons from this module.

First, the AANHPI Population is growing rapidly in size and diversity.

The Asian Monolith Bias masks the heterogeneity of the Asian American population

Hopefully through culturally competent clinical practices, community engagement, and the disaggregation of AANHPI representation in medical research, we can begin to break these disparities and move from **monolith to mosaic**

**Slide 49:**

Here are some recommended readings we have for you if you are interested!

**Slide 50:**

And here are some AANHPI CBOs in New York City that you may be interested in partnering with or checking out!
