# Supplementary material for: Deconstructing the Monolith: An Educational Module for Understanding Disparities Within Asian American, Native Hawaiian, and Pacific Islander Populations
Source: MedEdPORTAL. 2025 Jan 7;21:11480. doi: 10.15766/mep_2374-8265.11480 (PMC11697750; doi:10.15766/mep_2374-8265.11480)
Supplement: Supplementary file 1 — Monolith To Mosaic Presentation.pptxFacilitator Guide.docxPresurvey.docxPostsurvey.docx [file mep_2374-8265.11480-s001.zip › _Educational Summary Report_11480.pdf]

MedEdPORTAL® The AAMC Journal of Teaching and Learning Resources

Original Publication

Open Access

Deconstructing the Monolith: An Educational Module for Understanding Disparities Within Asian American, Native Hawaiian, and Pacific Islander Populations

Karan D. Luthria*, Dylan K. Kim, Samantha Xing, Mina Yuan, Jerry Zhou, MS, Catherine A. Shu, MD†, Usha S. Krishnan, MD†

*Corresponding author: kdl2130@cumc.columbia.edu

†Co-senior author

Abstract

Introduction: Asian American, Native Hawaiian, and Pacific Islander (AANHPI) people represent one of the largest and most rapidly growing groups in the United States and are often aggregated as a homogeneous, rather than diverse, population in medical research and education. Currently, few educational interventions focus on the disaggregation of AANHPI patient populations and the improvement of knowledge about health disparities that affect AANHPI patients. **Methods:** We developed, implemented, and facilitated a workshop for medical students to address AANHPI health disparities, adaptable for in-person and online formats. The 1-hour session involved a preworkshop evaluation; a PowerPoint presentation outlining the history of the Asian monolith bias, health disparities within AANHPI subgroups, and strategies for health care professionals and trainees to engage effectively with these communities; and a postworkshop evaluation. Pre- and postworkshop evaluations assessed participants' confidence and understanding of AANHPI health disparities. Additionally, the postworkshop evaluation gathered feedback on the presentation. **Results:** Pre- and postworkshop evaluations revealed that this workshop, attended by 42 diverse participants over two sessions, significantly improved participants' understanding of the Asian monolith bias and AANHPI health care disparities ($p < .05$). Whether attending virtually or in person, participants reported notable improvements in their self-evaluated confidence in treating AANHPI patients. **Conclusion:** The AANHPI patient population comprises a myriad of different cultures, historical contexts, and health needs. We present an educational module that is associated with significant improvement of knowledge about health disparities specific to this population, informing further efforts in cultural competence within medical education.

Keywords

Asian American, Native Hawaiian, Pacific Islander, Community-Based Medicine, Cultural Competence, Diversity, Equity, Inclusion, Health Equity, Population Health, Social Determinants of Health, Language-Appropriate Health Care

Educational Objectives

By the end of this module, the learner will be able to:

1. Describe the historical context of the Asian monolith stereotype in the United States.
2. Outline three ways in which the Asian monolith stereotype negatively impacts health outcomes for diverse Asian communities.
3. Identify strategies to improve health care equity for Asian patients and communities.

Citation:
Luthria KD, Kim DK, Xing S, et al. Deconstructing the monolith: an educational module for understanding disparities within Asian American, Native Hawaiian, and Pacific Islander populations. *MedEdPORTAL*. 2025;21:11480.
https://doi.org/10.15766/mep_2374-8265.11480

Introduction

The Asian American, Native Hawaiian, and Pacific Islander (AANHPI) community is one of the largest and most diverse populations in the United States, comprising 20.6 million individuals from 31 distinct self-identified groups.[1] As the fastest-growing racial group in the US, the AANHPI community's diversity is matched by significant economic contrasts, exhibiting the highest income inequality among racial groups in the US as of 2018.[2]

Copyright © 2025 Luthria et al. This is an open-access publication distributed under the terms of the Creative Commons Attribution license.

The AANHPI population often faces implicit bias that presents them as a homogeneous, rather than diverse, racial group—a concept defined as the Asian monolith bias.[3] This bias not only overlooks the rich cultural and historical differences between Asian communities but also results in significant implications in American culture, medical literature, and medical education. Culturally, AANHPI communities have collectively been depicted as the so-called model minority, a group that has achieved social mobility due to favorable norms and traits. While seemingly positive, this generalization has harmful effects by providing a basis to criticize other minority groups and overlooking the disparities within the AANHPI population itself. In the medical literature, AANHPI populations are often aggregated into a single group. For example, while 36% of studies in high-impact journals in 2015-2016 included Asian participants, only 0.8% explicitly incorporated data on specific AANHPI subgroups.[4] Consequently, medical literature often overlooks the health needs of specific subpopulations, impacting our understanding of health care utilization, disease prevalence, and patient-provider interactions.[5-8] This lack of focus on AANHPI heterogeneity is also present in medical education. Medical students of AANHPI identity are often classified as almost overrepresented despite many AANHPI subgroups being underrepresented in medicine.[9] In a qualitative study, medical students expressed the need for disaggregation of AANHPI populations in curricula and for educational materials on culturally competent health care delivery for AANHPI patients.[10] In an analysis of over 600 lectures within a single institution, Park and colleagues detected several instances of misidentification and aggregation of AANHPI identities, as well as the complete omission of AANHPI patient populations from lecture content.[11]

Numerous challenges arise from the Asian monolith generalization, yet there is a lack of support for research dedicated to the AANHPI population. Between 1992 and 2018, only 0.17% of the total budget of the National Institutes of Health was directed to projects that focused on the AANHPI population.[12] *MedEdPORTAL,* which offers educational resources on culturally competent care, racism, and microaggressions, has no publications that focus on needs of the Asian diaspora.[13-15] Research demonstrates that culturally tailored interactions in the AANHPI patient population result in improved outcomes, making medical education that provides awareness of such skills paramount to health equity.[16-18] The purpose of this module is to improve knowledge of health disparities that affect AANHPI communities, as well as to provide culturally competent strategies to ensure equitable care for AANHPI patients.

Methods

Educational Approach

The design, implementation, and evaluation of this module adhered to the six-step Kern model for curriculum development.[19] For step 1, problem identification and general needs assessment, we conducted a literature review and discussed our findings with physicians and faculty mentors. For step 2, targeted needs assessment, we reviewed literature on the representation of AANHPI health issues within medical school curricula, as described above. For step 3, goals and objectives, we formulated a list of learning objectives with feedback from coauthors and external mentors. For step 4, educational strategies, we included a didactic PowerPoint presentation containing interactive questions and a case illustrating the clinical manifestation of AANHPI health disparities. For step 5, implementation, the 1-hour workshop was administered in fall 2023 and made available in two formats: (1) an in-person format for medical students at the authors' home institution and (2) a virtual format via Zoom for medical students nationwide. For step 6, evaluation and feedback, participants were provided with pre- and postworkshop surveys to assess their understanding and critique the workshop's design and content. Surveys were anonymized and administered through an institutionally approved Qualtrics platform. Questions included assessment of knowledge of module content, preparedness in meeting learning objectives, and confidence in understanding of AANHPI health disparities and patient care.

Facilitators and Target Audience

In both the virtual and in-person implementations, this module was administered by a team of five medical students interested in working with AANHPI patients. Facilitators reviewed the slide deck, pre- and postpresentation evaluation forms, and facilitator's guide prior to presenting the module. Designed for physicians-in-training, the workshop was accessible to medical students from any demographic and did not require a specific knowledge base for participation, making it inclusive for all levels of medical education. The content focused on enriching the curriculum of those in their preclinical years of medical school. However, the module also contained elements suitable for medical students in their third and fourth years, applying knowledge from their clinical rotations. Each facilitator's familiarity with every segment of the module ensured flexibility and shared responsibility in case of any absences.

Workshop Materials

Review of the appendix materials took approximately 2 hours. While following along with the PowerPoint slide deck

Copyright © 2025 Luthria et al. This is an open-access publication distributed under the terms of the Creative Commons Attribution license.

(Appendix A), all facilitators read over the facilitator's guide (Appendix B) and determined how to divide the presentation appropriately. Facilitators were encouraged to complete a practice session. In order for participants to complete the preworkshop and postworkshop evaluation, facilitators could embed QR codes into the provided PowerPoint or print out physical copies.

The total workshop duration, including evaluation time and a preliminary period for attendee briefing, was approximately 60 minutes. Provided with audiovisual equipment to display slides and communicate with the audience, the appendices contained the remaining resources necessary to implement the workshop successfully:

- Appendix A: PowerPoint presentation: The content of the workshop was provided in a 50-slide PowerPoint presentation, which outlined the objectives of the workshop and featured a brief description and history of the Asian monolith bias, various health disparities among AANHPI subgroups and how to tackle them, and a clinical case displaying a physician's or medical student's role in dismantling the Asian monolith bias.
- Appendix B: facilitator's guide: The flow and content of the workshop were detailed in a 12-page document. This guide matched each PowerPoint slide to key points facilitators needed to mention verbally and provided timing suggestions for optimal interactivity. It could be used for both the in-person and Zoom versions of the workshop.
- Appendix C: preworkshop evaluation form: This was a 15-question evaluation distributed to participants before the workshop to assess prior knowledge about Asian monolith bias and to collect their demographic information. Participants were also asked to rate their confidence and knowledge about AANHPI health disparities as well as their medical school's curricular emphasis on these topics.
- Appendix D: postworkshop evaluation form: This was a 14-question evaluation distributed to participants after the workshop. This evaluation contained the same multiple-choice and Likert-type questions as the preworkshop evaluation form, with additional questions eliciting participant feedback about the quality of the workshop.

Workshop Timeline and Implementation
Utilizing the PowerPoint (Appendix A) and instructions from the facilitator's guide (Appendix B), the workshop began with an introduction from the facilitator team, followed by a discussion of the learning objectives. Participants were encouraged to fill out the preworkshop evaluation form (Appendix C) during this time, with QR codes embedded in the PowerPoint and physical copies of the survey provided.

The first 10-minute segment of the module introduced the Asian monolith bias and its historical context. The next two 10-minute segments were dedicated to discussion of specific examples of health disparities in the AANHPI population and strategies to improve health equity through clinical practice, research, and advocacy. The final 10-minute segment of the module presented a clinical case through audience response role-playing scenarios, demonstrating further manifestations of the Asian monolith bias. Interactive activities for the audience via real-time polls were provided throughout the module. The module concluded with key takeaways and ways to engage in local community efforts to improve AANHPI health care equity. Participants were provided with a postworkshop survey (Appendix D) to evaluate the module.

Institutional Review Board Approval
The evaluation design was approved by the Columbia University Institutional Review Board (protocol #AAAU7951).

Workshop Adjustments
Iterative rounds of feedback for this workshop prior to initial administration were provided by faculty members with expertise in medical education and working with AANHPI patients. Additional adjustments to the module were incorporated from participants. We included community-based organizations actively working within AANHPI communities to connect participants with groups tackling health disparities. We also adjusted the clinical case presentations to better reflect nuances of health care delivery. Additional data highlighting disparities specifically affecting Pacific Islander populations were emphasized, offering a broader perspective on the challenges faced by groups within the AANHPI umbrella. Finally, to set a clear context for our discussions, we revised the module to begin with a description of the AANHPI community within the US.

Workshop Analysis
Comparison between pre- and postworkshop knowledge-based questions was conducted using two-sample one-tailed z tests for differences in proportions. Questions based on participants' level of preparedness in completing the given learning objectives were converted to a 4-point Likert-type scale (1 = *not at all prepared,* 4 = *very prepared*). We also evaluated participants' agreement with certain statements on a 5-point Likert-type scale (1 = *strongly disagree,* 5 = *strongly agree*). Differences in preparedness and agreements between pre- and postworkshop

Copyright © 2025 Luthria et al. This is an open-access publication distributed under the terms of the Creative Commons Attribution license.

evaluations were calculated using a two-sample two-tailed *t* test. A *p* value of $<.05$ was considered statistically significant in this evaluation.

Results

Of the 54 attendees across both sessions, 42 medical students completed both the preworkshop and postworkshop surveys: 36 (86%) from the in-person session and six (14%) from the virtual session. Participants were from multiple medical institutions across four states and all 4 years of medical school, with a majority ($n = 39$, $>90\%$) self-identified as first- or second-year students (Table 1).

Results from the multiple-choice questions to assess participants' knowledge of AANHPI health care disparities showed a statistically significant improvement in correct responses to four of five questions after the workshop, with consistent increases across both virtual and in-person formats and for both AANHPI and non-AANHPI participants (Table 2).

Pre- and postworkshop surveys also asked about participants' preparedness regarding the assigned learning objectives. For each objective, there was a significant increase in preparedness after the module, with mean ratings on a Likert-type 4-point scale improving from 2.0, 1.9, and 1.8 to 3.3, 3.3, and 3.2, respectively (Table 3). Participants were also evaluated for their perceived confidence in treating AANHPI patients and their awareness of AANHPI diversity. Notably, while a majority of AANHPI participants initially felt knowledgeable about AANHPI diversity, with mean ratings of 3.6 on a Likert-type 5-point scale, this increased significantly to 4.1 following the workshop. This change was more pronounced among non-AANHPI participants, where self-perceived knowledge rose from a mean of 2.6 to 4.3 (Table 4). These trends remained consistent regardless of whether the workshop was conducted in person or virtually.

Table 1. Characteristics of Workshop Participants ($n = 42$)

Characteristic	No. (%)
Race/ethnicity	
Asian Indian	9 (21)
Black or African	3 (7)
Chinese	14 (33)
Filipino/a	2 (5)
Japanese	2 (5)
Korean	2 (5)
Latino/a	3 (7)
Samoan	1 (2)
Taiwanese	2 (5)
Vietnamese	2 (5)
White	10 (24)
Year in medical school	
1	20 (48)
2	19 (45)
3	2 (5)
4	1 (2)
State of residence	
Connecticut	2 (5)
Massachusetts	1 (2)
New York	38 (91)
Wisconsin	1 (2)

Qualitative feedback regarding the module's strengths focused on (1) the organization of module content, (2) the use of specific examples and a clinical case, and (3) audience engagement with interactive components. Selected examples of this feedback are summarized below.

1. Organization of module content:
   - "It was very organized and had clear ways that we as med students can help tackle the Asian monolith."
   - "Breaks down the data in easy to understand comparisons and has actionable items for med students."
2. Use of specific examples and a clinical case:
   - "Good overview of AAPI health disparities with concrete examples."
   - "Really great overview on the Asian monolith, great specific examples."
3. Audience engagement with interactive components:
   - "The questions throughout make the module very engaging."
   - "Liked the interactive aspects with [Poll Everywhere] and the case study."

Participants also commented on improvements for the module, as provided below. Many of these comments revolved around limited exploration of disparities impacting certain AANHPI communities, the addition of real patient stories, and providing key takeaway points for students.

- "There wasn't really mention of the NHPI part, it's a common theme for AANHPI workshops to be more AA(nhpi) so it would've been nice to better acknowledge the NHPI part."
- "Not much Native Hawaiian/Pacific Islander data included.... could be nice to suggest how this can be incorporated in preclinical."
- "Could be nice to have a handout with the key info and takeaways for attendees to take."
- "Have some more specific examples like patient stories and experiences to highlight some of the issues."

Finally, we evaluated students' perceptions regarding the coverage of AANHPI communities in their school curriculum.

Copyright © 2025 Luthria et al. This is an open-access publication distributed under the terms of the Creative Commons Attribution license.

Table 2. Percentage of Correct Answers to Pre- and Postmodule Knowledge Questions

Knowledge Questions and Answers	All Participants (*n* = 42)			In-person (*n* = 36)			Virtual (*n* = 6)			AANHPI (*n* = 27)			Non-AANHPI (*n* = 15)		
	Pre (%)	Post (%)	*p*	Pre (%)	Post (%)	*p*	Pre (%)	Post (%)	*p*	Pre (%)	Post (%)	*p*	Pre (%)	Post (%)	*p*
Q: Which movement in health care research describes the pushback against averaging the data of all Asians into one category? A: Disaggregation	76	91	.04	78	92	.05	67	83	.25	83	90	.22	67	93	.03
Q: Which of the following AANHPI subgroups represented the smallest percentage of US medical school applicants in 2018? A: Laotian	43	91	<.01	44	92	<.01	33	83	.02	45	90	<.01	33	87	<.01
Q: Which of the following AANHPI subgroups has the lowest rates of mammogram screening? A: Korean	12	93	<.01	8	97	<.01	33	67	.11	7	93	<.01	20	93	<.01
Q: Which of the following AANHPI subgroups has the highest smoking rates? A: Filipino	26	79	<.01	28	83	<.01	17	50	.10	28	76	<.01	27	87	<.01
Q: What are "culture-bound syndromes" in the context of health care? A: Syndromes that are relatively common within certain cultures but not officially acknowledged in Western medicine.	86	95	.07	86	97	.04	83	83	.50	86	100	.02	87	87	.50

Abbreviation: AANHPI, Asian American, Native Hawaiian, and Pacific Islander.

Following the module, most students either disagreed or strongly disagreed with the statement that their medical school did a good job of teaching the different health care needs of AANHPI subpopulations, with an average score of 2.6 on a 5-point Likert-type scale (Table 4). Qualitative feedback supported this finding. Comments like "I did not know much of the information here. Definitely a very important topic that all medical students should be aware of" and "Thank you for shedding light on this especially since we never covered it in classes" highlighted the educational gap. Participant feedback for this module also included the sentiment that this content "should be required" by medical schools.

Discussion

As in other fields of medicine, medical education has often misrepresented AANHPI populations, overlooking their diversity

Table 3. Participant Self-Reported Learning Objective Preparedness Pre- and Postmodule

Learning Objective[a]	Premodule *M* (95% CI)	Postmodule *M* (95% CI)	*M* Difference	*p*
Describing the historical context of the Asian monolith stereotype in the United States				
All participants	2.0 (1.7-2.2)	3.3 (3.1-3.5)	1.4	<.01
Virtual	2.2 (0.8-3.6)	3.3 (2.5-4.2)	1.2	.01
In person	1.9 (1.7-2.2)	3.3 (3.1-3.5)	1.4	<.01
AANHPI	2.1 (1.8-2.4)	3.4 (3.2-3.6)	1.3	<.01
Non-AANHPI	1.7 (1.3-2.1)	3.2 (2.8-3.6)	1.5	<.01
Outlining three ways in which the Asian monolith stereotype negatively impacts health outcomes for diverse Asian communities				
All participants	1.9 (1.6-2.2)	3.3 (3.2-3.5)	1.4	<.01
Virtual	1.8 (0.6-3.1)	3.5 (2.9-4.1)	1.7	<.01
In person	1.9 (1.6-2.2)	3.3 (3.1-3.5)	1.4	<.01
AANHPI	2.0 (1.7-2.4)	3.4 (3.1-3.6)	1.3	<.01
Non-AANHPI	1.6 (1.2-2.0)	3.2 (3.0-3.4)	1.6	<.01
Identifying strategies to improve health care equity for Asian patients and communities				
All participants	1.8 (1.5-2.1)	3.2 (3.0-3.3)	1.4	<.01
Virtual	1.7 (0.4-2.9)	3.3 (2.8-3.9)	1.7	<.01
In person	1.8 (1.6-2.1)	3.1 (2.9-3.3)	1.3	<.01
AANHPI	1.9 (1.5-2.2)	3.2 (3.0-3.4)	1.3	<.01
Non-AANHPI	1.7 (1.3-2.0)	3.1 (2.7-3.4)	1.4	<.01

Abbreviations: AANHPA, Asian American, Native Hawaiian, and Pacific Islander; CI, confidence interval.
[a]Rated on a 4-point scale (1 = *not at all prepared*, 4 = *very prepared*).

Copyright © 2025 Luthria et al. This is an open-access publication distributed under the terms of the Creative Commons Attribution license.

Table 4. Participant Self-Reported Agreement Levels Pre- and Postmodule

Evaluation Statement[a]	Premodule *M* (95% CI)	Postmodule *M* (95% CI)	*M* Difference	*p*
I am confident in my ability to take care of AANPHI patients.				
All participants	3.5 (3.2-3.7)	4.0 (3.8-4.2)	0.5	<.01
Virtual	3.5 (2.4-4.6)	3.8 (3.0-4.6)	0.3	.17
In person	3.5 (3.2-3.7)	4.1 (3.9-4.2)	0.6	<.01
AANHPI	3.6 (3.3-3.8)	4.1 (3.9-4.3)	0.5	<.01
Non-AANHPI	3.3 (3.0-3.7)	3.9 (3.6-4.3)	0.6	<.01
I am knowledgeable about the diversity of the AANHPI patients.				
All participants	3.3 (3.0-3.6)	4.2 (4.0-4.4)	0.9	<.01
Virtual	3.2 (1.9-4.4)	4.2 (3.4-5.0)	1.0	.01
In person	3.3 (3.0-3.6)	4.2 (4.0-4.4)	0.9	<.01
AANHPI	3.6 (3.2-3.9)	4.1 (3.9-4.3)	0.6	<.01
Non-AANHPI	2.6 (2.2-3.0)	4.3 (4.1-4.6)	1.7	<.01
My medical school does a good job at teaching the different health care needs for AANHPI subpopulations.				
All participants	2.1 (1.8-2.4)	2.6 (2.2-3.0)	0.5	<.01
Virtual	2.0 (0.9-3.1)	1.8 (1.0-2.6)	−0.2	.36
In person	2.1 (1.8-2.5)	2.7 (2.3-3.1)	0.6	<.01
AANHPI	2.1 (1.7-2.4)	2.5 (2.0-3.0)	0.4	<.01
Non-AANHPI	2.1 (1.5-2.6)	2.6 (1.9-3.3)	0.5	<.01

Abbreviations: AANHPI, Asian American, Native Hawaiian, and Pacific Islanders; CI, confidence interval.
[a]Rated on a 5-point scale (1 = *strongly disagree,* 5 = *strongly agree*).

by portraying them as a homogeneous group.[10] Hence, medical students have expressed concerns that current curricula fail to address the nuances of culturally competent health care for AANHPI patients.[9] Our module, specifically designed to bridge these gaps, demonstrates effectiveness in enhancing US medical students' understanding of health disparities and the widespread misrepresentation of AANHPI communities in health care research. This initiative represents a novel effort in medical education to elucidate the historical context of the Asian monolith bias and address health care disparities affecting AANHPI communities.

Utilizing interactive methods such as polling, audience engagement, and visual aids, our hour-long module significantly increased students' self-reported knowledge and awareness of the Asian monolith bias and various health care disparities affecting AANHPI populations. The module set clear objectives: to elucidate the historical backdrop of the Asian monolith stereotype, to identify at least three ways this stereotype adversely affects health outcomes in Asian communities, and to propose strategies for enhancing health care equity. Our qualitative assessments indicated substantial self-reported knowledge gains among medical students in these areas. Additionally, by following the six-step Kern model,[19] we continuously refined the module to enhance its delivery in subsequent iterations.

Participant and faculty feedback highlighted several strengths of this presentation, including module organization, inclusion of specific examples and clinical scenarios, and use of audience polling and interactivity. The module's structure, starting with the historical context of the Asian monolith bias, moving to current health disparities, and ending with intervention methods, was well received. Throughout, the inclusion of specific examples and clinical scenarios with audience polling effectively maintained participant interest.

Participant feedback also mentioned several areas for improvement. One suggestion was adapting the module to specific audiences. Examples include offering case studies discussing AANHPI disparities present in specific geographic areas, such as New York City, thereby tailoring the content to local medical students. Participants also recommended incorporating actionable best practices for future medical practitioners and students entering clerkship rotations in order to apply this learning in real-world settings. Additionally, there was a desire for more in-depth coverage of specific AANHPI communities. The module underwent an iterative revision process to include these topics within its 1-hour time frame.

Both the virtual and in-person workshop sessions showed significant improvements among participants in preparedness, knowledge, and confidence regarding addressing AANHPI health disparities. We did note significantly lower attendance numbers during the virtual workshop session, possibly due to scheduling conflicts or digital fatigue. Performance did not vary based on audience background, as both AANHPI and non-AANHPI participants showed improvement in their awareness of AANHPI diversity, indicating the module's broad applicability.

A facilitator's guide is provided so that individuals from any background can present this module. The guide permits facilitator

Copyright © 2025 Luthria et al. This is an open-access publication distributed under the terms of the Creative Commons Attribution license.

discretion in selecting effective methods to engage audiences across different settings. This workshop can be used as a stand-alone resource or be integrated into a broader curriculum addressing racial and ethnic health disparities. Looking ahead, the national Asian Pacific American Medical Student Association can also assist facilitators in disseminating the workshop across institutions.

This module has several limitations. First, the 1-hour length restricts the depth of discussion on AANHPI health disparities, preventing coverage of key issues affecting specific AANHPI communities. However, the workshop provides a solid foundation for educating students about these disparities, offering historical context, specific examples, and recommendations to improve health equity for AANHPI patients. Second, participant assessments were limited to immediate pre- and postworkshop evaluations that could not measure long-term knowledge retention. These evaluations also focused on self-reported learning and confidence, which did not directly measure whether the workshop's specific knowledge-based objectives were met. Additionally, the workshop was primarily delivered to preclinical students from one medical institution. To comprehensively evaluate the module, it should be assessed across diverse educational settings and its impact on clinical care measured. Lastly, our evaluations did not gauge attendees' familiarity and interest in working with AANHPI patients, which could influence engagement and learning outcomes.

These limitations set the stage for next steps to improve the representation of AANHPI populations in medical education. The module could be adapted in additional formats (such as asynchronous modules and small-group discussions) to accommodate different learning settings and facilitate evaluation of long-term knowledge retention. Future work could broaden the audience to include medical residents and attendings, as education on cultural humility is a continuous journey. Lastly, similar medical education initiatives could investigate health disparities in other patient populations within the AANHPI community.

In summary, this work addresses the misrepresentation of AANHPI communities in medical education. With an iterative development process, we have created an hour-long module containing several interactive elements that enhance medical students' understanding of the Asian monolith bias and its ongoing impact on patient outcomes. The module has proved effective in both virtual and in-person settings and is accessible to a wide range of facilitators. It communicates historical context, prevalence of health disparities, and strategies for improving health outcomes for AANHPI communities. We believe that this work is a crucial addition to US medical education curricula, offering an educational intervention to improve health equity.

Appendices

A. Monolith To Mosaic Presentation.pptx

B. Facilitator Guide.docx

C. Presurvey.docx

D. Postsurvey.docx

All appendices are peer reviewed as integral parts of the Original Publication.

Karan D. Luthria: Third-Year MD-PhD Student, Columbia University Vagelos College of Physicians and Surgeons; ORCID: https://orcid.org/0000-0002-4392-9675

Dylan K. Kim: Third-Year Medical Student, Columbia University Vagelos College of Physicians and Surgeons; ORCID: https://orcid.org/0009-0001-7516-7142

Samantha Xing: Third-Year Medical Student, Columbia University Vagelos College of Physicians and Surgeons; ORCID: https://orcid.org/0000-0003-3525-9621

Mina Yuan: Third-Year Medical Student, Columbia University Vagelos College of Physicians and Surgeons

Jerry Zhou, MS: Third-Year Medical Student, Columbia University Vagelos College of Physicians and Surgeons

Catherine A. Shu, MD: Associate Professor, Department of Medicine, Columbia University Irving Medical Center; ORCID: https://orcid.org/0000-0003-0765-7665

Usha S. Krishnan, MD: Professor, Department of Pediatrics, Columbia University Irving Medical Center

Acknowledgments
The authors would like to thank Dr. John Paul Sánchez and Mrs. Kaitlyn Pommells from the Building the Next Generation of Academic Physicians (BNGAP)-National Board of Medical Examiners (NBME) Academic Writing Fellowship for their exceptional guidance and mentorship; Dr. Melissa Palma, Clinical Assistant Professor of Family Medicine at Brown University, for her valuable feedback; and Dr. Monica Lypson and Dr. Rosa Lee, Deans at Columbia University's Vagelos College of Physicians and Surgeons, for providing institutional support to carry out this project.

Disclosures
All identifiable persons in this resource have granted their permission.

Funding/Support
This educational module was implemented with financial support from the Building the Next Generation of Academic Physicians (BNGAP)-

Copyright © 2025 Luthria et al. This is an open-access publication distributed under the terms of the Creative Commons Attribution license.

National Board of Medical Examiners (NBME) Academic Writing Fellowship, the national Asian Pacific American Medical Student Association's Community Outreach Grant, and the Columbia University Vagelos College of Physicians and Surgeons Office of Student Diversity, Inclusion, and Belonging. Karan D. Luthria is supported by Training Grant T32GM145440 from the Foundation for the National Institutes of Health.

Ethical Approval

The Columbia University Institutional Review Board approved this project.

References

1. Monte LM, Shin HB. 20.6 million people in the U.S. identify as Asian, Native Hawaiian or Pacific Islander. United States Census Bureau. 2022. Accessed December 16, 2024. https://www.census.gov/library/stories/2022/05/aanhpi-population-diverse-geographically-dispersed.html
2. Budiman A, Ruiz NG. Asian Americans are the fastest-growing racial or ethnic group in the U.S. Pew Research Center. 2021. Accessed December 16, 2024. https://www.pewresearch.org/short-reads/2021/04/09/asian-americans-are-the-fastest-growing-racial-or-ethnic-group-in-the-u-s/
3. Đoàn LN, Chau MM, Ahmed N, Cao J, Chan SWC, Yi SS. Turning the health equity lens to diversity in Asian American health profiles. *Annu Rev Public Health.* 2024;45:169-193. https://doi.org/10.1146/annurev-publhealth-060222-023852
4. Nguyen HT, Zheng A, Gugel A, Kistin CJ. Asians and Asian subgroups are underrepresented in medical research studies published in high-impact generalist journals. *J Immigr Minor Health*. 2021;23(3):646-649. https://doi.org/10.1007/s10903-021-01142-6
5. Yu SM, Huang ZJ, Singh GK. Health status and health services access and utilization among Chinese, Filipino, Japanese, Korean, South Asian, and Vietnamese children in California. *Am J Public Health*. 2010;100(5):823-830. https://doi.org/10.2105/AJPH.2009.168948
6. Torre LA, Sauer AMG, Jr Chen MS, Kagawa-Singer M, Jemal A, Siegel RL. Cancer statistics for Asian Americans, Native Hawaiians, and Pacific Islanders, 2016: converging incidence in males and females. *CA Cancer J Clin.* 2016;66(3):182-202. https://doi.org/10.3322/caac.21335
7. Ngo-Metzger Q, Sorkin DH, Phillips RS. Healthcare experiences of limited English-proficient Asian American patients: a cross-sectional mail survey. *Patient.* 2009;2(2):113-120. https://doi.org/10.2165/01312067-200902020-00007
8. Liu CZ, Wang E, Nguyen D, Sun MD, Jumreornvong O. The model minority myth, data aggregation, and the role of medical schools in combating anti-Asian sentiment. *Acad Med.* 2022;97(6): 797-803. https://doi.org/10.1097/ACM.0000000000004639
9. Ko M, Ton H. The not underrepresented minorities: Asian Americans, diversity, and admissions. *Acad Med*. 2020;95(2): 184-189. https://doi.org/10.1097/ACM.0000000000003019
10. Le TK, Vongsachang H, Pang S, et al. US medical student perspectives on Asian American patient inclusion in medical education: a qualitative study. *BMC Med Educ*. 2022;22:482. https://doi.org/10.1186/s12909-022-03550-0
11. Park PSU, Algur E, Narayan S, Song WB, Kearney MD, Aysola J. Representation of Asian American populations in medical school curricula. *JAMA Netw Open*. 2022;5(9):e2233080. https://doi.org/10.1001/jamanetworkopen.2022.33080
12. Đoàn LN, Takata Y, Sakuma KK, Irvin VL. Trends in clinical research including Asian American, Native Hawaiian, and Pacific Islander participants funded by the US National Institutes of Health, 1992 to 2018. *JAMA Netw Open*. 2019;2(7):e197432. https://doi.org/10.1001/jamanetworkopen.2019.7432
13. Allwardt D. Culturally competent health care practice with older adults. *MedEdPORTAL*. 2006:2;254. https://doi.org/10.15766/mep_2374-8265.254
14. DallaPiazza M, Padilla-Register M, Dwarakanath M, Obamedo E, Hill J, Soto-Greene ML. Exploring racism and health: an intensive interactive session for medical students. *MedEdPORTAL*. 2018; 14:10783. https://doi.org/10.15766/mep_2374-8265.10783
15. Acholonu RG, Cook TE, Roswell RO, Greene RE. Interrupting microaggressions in health care settings: a guide for teaching medical students. *MedEdPORTAL*. 2020;16:10969. https://doi.org/10.15766/mep_2374-8265.10969
16. Lei F, Zheng Y, Lee E. Interventions for cancer screening among Chinese Americans: a systematic review and meta-analysis. *PLoS One.* 2022;17(3):e0265201. https://doi.org/10.1371/journal.pone.0265201
17. Alfred M, Ubogaya K, Chen X, Wint D, Worral PS. Effectiveness of culturally focused interventions in increasing the satisfaction of hospitalized Asian patients: a systematic review. *JBI Database System Rev Implement Rep*. 2016;14(8):219-256. https://doi.org/10.11124/JBISRIR-2016-003048
18. Rivera AM, Zhang Z, Kim A, Ahuja N, Lee HY, Hahm HC. Mechanisms of action in AWARE: a culturally informed intervention for 1.5- and 2nd-generation Asian American women. *Am J Orthopsychiatry.* 2019;89(4):475-481. https://doi.org/10.1037/ort0000391
19. Thomas PA, Kern DE, Hughes MT, Chen BY, eds. *Curriculum Development for Medical Education: A Six-Step Approach.* 3rd ed. Johns Hopkins University Press; 2016.

Received: January 9, 2024
Accepted: September 25, 2024
Published: January 7, 2025

Copyright © 2025 Luthria et al. This is an open-access publication distributed under the terms of the Creative Commons Attribution license.
